# Supplementary material for: Neutralization of acyl coenzyme A binding protein for the experimental prevention and treatment of hepatocellular carcinoma
Source: Cell Rep Med. 2025 Jul 7;6(7):102232. doi: 10.1016/j.xcrm.2025.102232 (PMC12281431; doi:10.1016/j.xcrm.2025.102232)
Supplement: Document S1. Figures S1–S15 and Table S1 [file mmc1.pdf]

Cell Reports Medicine, Volume *6*

Supplemental information

Neutralization of acyl coenzyme A binding protein for the experimental prevention and treatment of hepatocellular carcinoma

Sijing Li, Omar Motiño, Flavia Lambertucci, Jonathan Pol, Hui Chen, Long Pan, Sylvère Durand, Federica Rossin, Claudia Campani, Lucie Poupel, Christophe Klein, Léa Montégut, María Pérez-Lanzón, Gerasimos Anagnostopoulos, Uxia Nogueira-Recalde, Alexandra Cerone, Fanny Aprahamian, Yanbing Dong, Manuela Lizarralde-Guerrero, Enfu Xue, Peng Liu, Liwei Zhao, Hui Pan, Vincent Carbonnier, Sylvie Lachkar, Ester Gloria Saavedra Díaz, Li Sun, Chantal Desdouets, Sabine Colnot, Oliver Kepp, Isabelle Martins, Laurence Zitvogel, Mauro Piacentini, Jean-Charles Nault, Maria Chiara Maiuri, Jessica Zucman-Rossi, and Guido Kroemer

Supplemental Figures

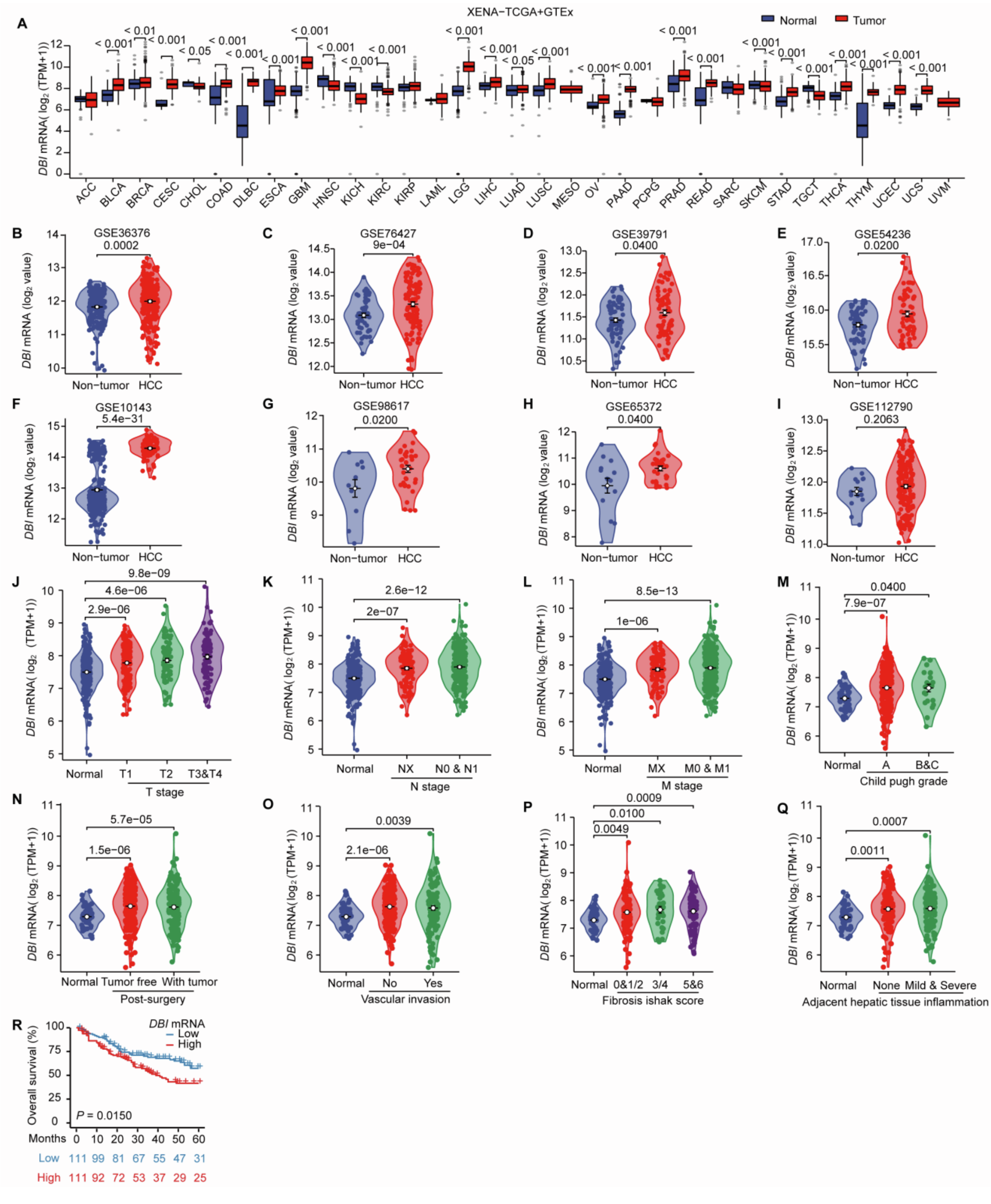

Figure S1 (related to Figure 1). Differential *DBI* mRNA expression in TCGA database. (A) Pan-cancer *DBI* mRNA expression profiles in GEO and TCGA databanks (n = 3-1113/group). (B-I) *DBI* mRNA is significantly upregulated in HCC tissues versus non-tumor tissues in various GEO datasets, including GSE36376 (n = 193-240/group), GSE76427 (n = 52-115/group), GSE39791 (n = 70/group), GSE54236 (n = 64/group), GSE10143 (n = 81-306/group), GSE98617 (n = 11-35/group), GSE65372 (n = 14-33/group), and GSE112790 (n = 15-176/group). (J-M) Changes of *DBI* mRNA in TCGA-LIHC database based on TNM stages and Child-Pugh grade (n = 22-276/group). (N-Q) Differential *DBI* mRNA expression in TCGA-LIHC database with regard to clinical and tumor

characteristics including tumor status, vascular invasion, fibrosis and inflammation (n = 30-212/group). *P* values were calculated by Mann-Whitney U test (A, B, E, F), Welch's t test (C, D, H, I), T test (G), Kruskal-Wallis test with Dunn's post-hoc test (J, K, L, P), and Welch's one-way ANOVA with Games-Howell test (M, N, O, Q). (R) Overall survival of HCC patients based on *DBI* mRNA expression in tumors (n = 111/group). The *P* value was calculated by log-rank test.

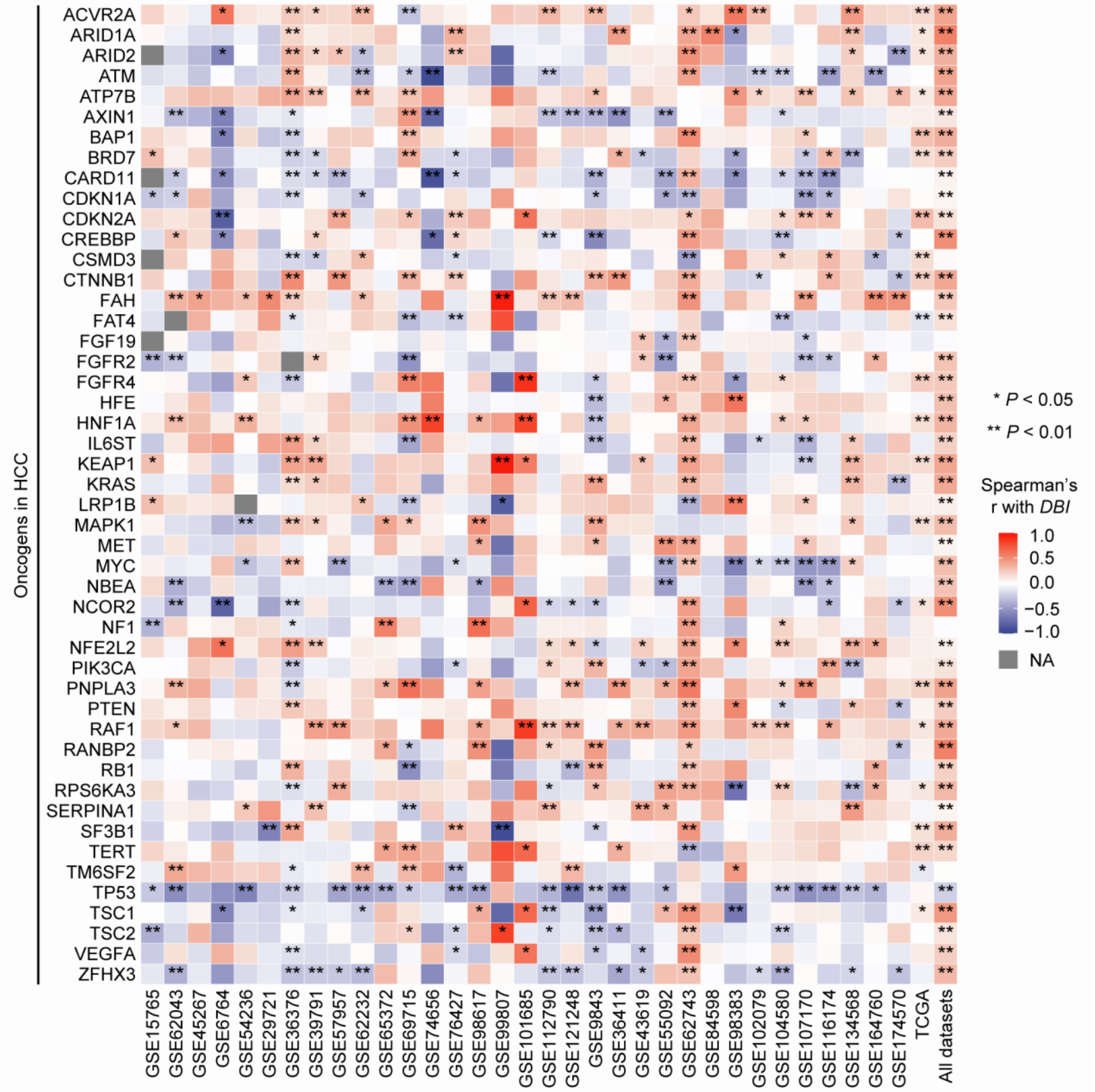

Figure S2 (related to Figure 1). Correlations of ACBP/DBI mRNA levels with mRNAs encoded by oncogenes relevant to human hepatocellular carcinoma. Spearman's coefficient (r) and *P* values were presented in the heatmap for the indicated GSE datasets. NA: not available. **P*<0.05, ***P*<0.01.

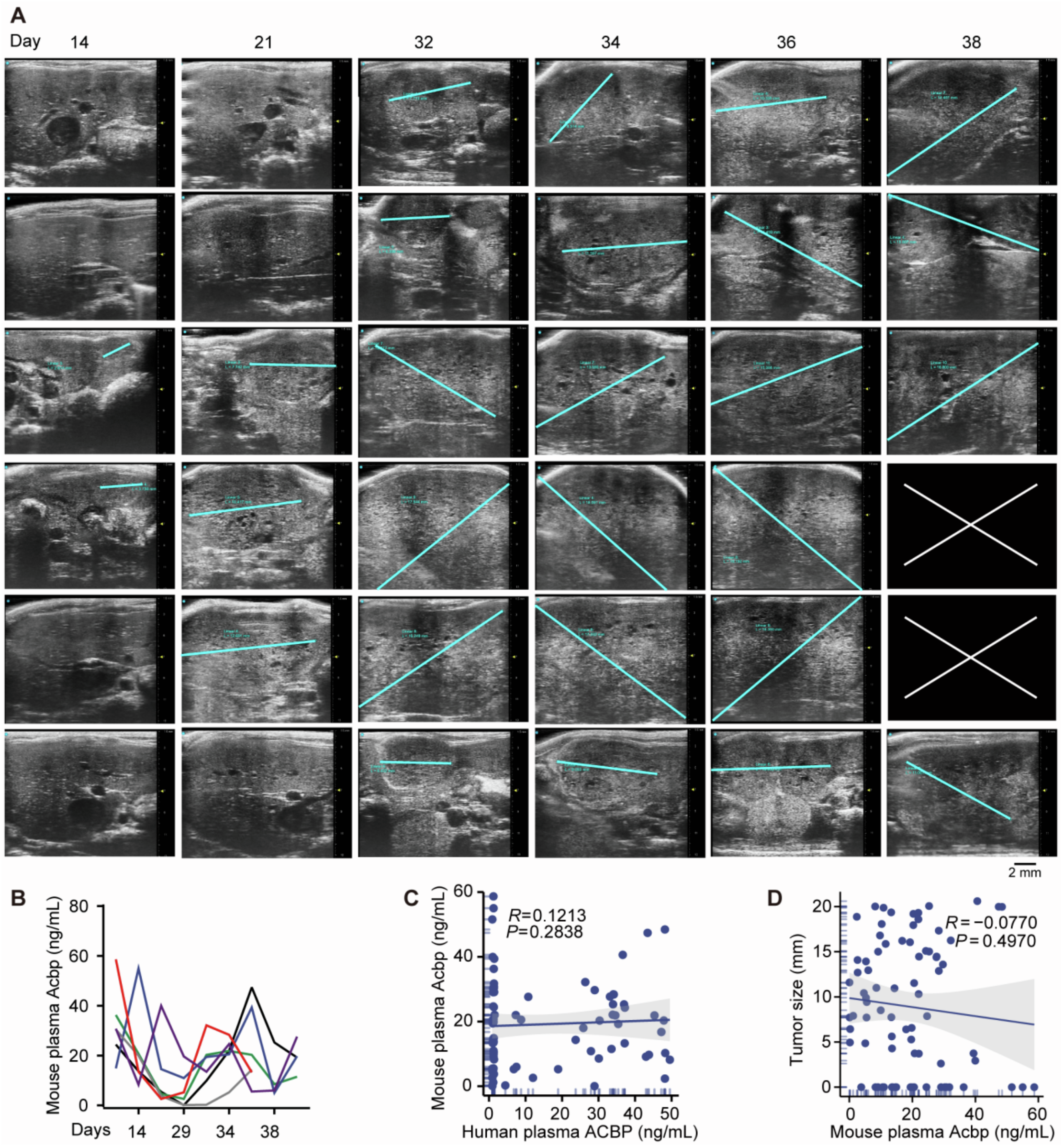

Figure S3 (related to Figure 1). Effect of orthotopic HUH-7 cells implanted in immunodeficient mice on ACBP/DBI levels. (A) Representative images of orthotopic HUH-7 tumors monitored by ultrasonography in nude mice (n = 6). The blue line indicates maximal diameter of a tumor. Scale bar represents 2 mm. Crosses indicate that mice had reached the humane endpoint. (B-D) Effects of HUH-7 cancers inoculated into the liver of immunodeficient *nu/nu* mice (referred to Figure 1M-O) on mouse ACBP/DBI plasma levels over time for individual mice (B) (n = 6), correlation with human ACBP plasma levels (D) (n = 18) and correlation with tumor size (E) (n = 18).

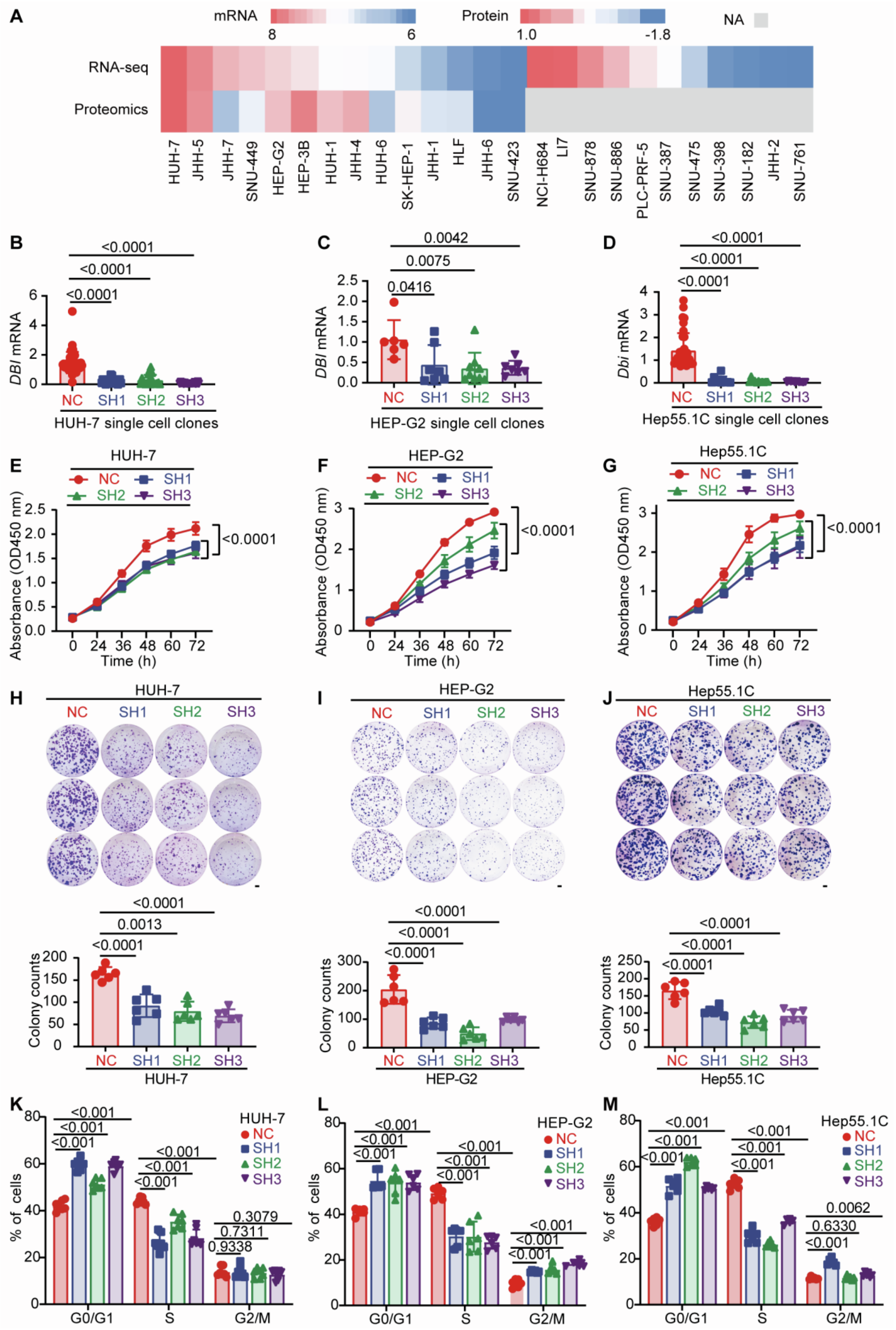

Figure S4 (related to Figure 2). Knockdown of *DBI/Dbi* attenuates proliferation, reduces clonogenic potential and arrests the cell cycle of liver cancer cells. (A) ACBP/DBI mRNA and protein expression profiles in different liver cancer cell lines. Data were extracted from the CCLE database (https://sites.broadinstitute.org/ccle/). (B-D) *DBI/Dbi* depletion efficiency verified by

qRT-PCR in single cell clones, derived from distinct parental cell lines including HUH-7, HEP-G2 and Hep55.1C (n = 6-43/group). (E-G) CCK-8 proliferation assay of HUH-7, HEP-G2 and Hep55.1C-derived *DBI/Dbi* knockdown and control cell lines (n = 6/group). (H-J) Representative images and quantifications of colony formation assays (n = 6/group) and (K-M) cytofluorometric analysis of cell cycle distributions of the aforementioned liver cancer cell lines. Experiments were performed at least three times (n = 6/group). Scale bar represents 3.5 mm. Error bars represent means ± SEM. *P* values were calculated by Kruskal-Wallis test with Dunn's post-hoc test (B, C, D), two-way ANOVA (E, F, G), one-way ANOVA post hoc Tukey HSD test (H, J), Welch's one-way ANOVA with Games-Howell test (I), and Kruskal-Wallis test with Dunn's post-hoc test (K, L, M), and Mann-Whitney U (P).

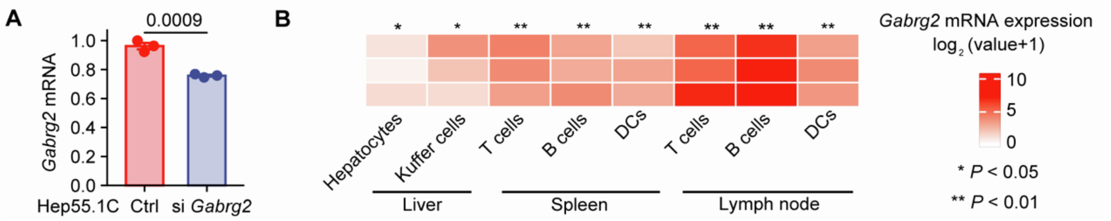

Figure S5 (related to Figure 2). *Gabrg2* mRNA expression in various cell types. (A) Specificity of the *Gabrg2* primer, as assessed by qRT-PCR in negative control (Ctrl) and *Gabrg2*-deficient (si *Gabrg2*) Hep55.1C cells (n = 3/group). The gene 36b4 was utilized as a housekeeping gene. Statistical significance (Ctrl vs. Si *Gabrg2*) was determined using Student's t-test. (B) *Gabrg2* mRNA expression profile in isolated primary hepatocytes, Kupffer cell from liver and immune cells from the spleen and lymph nodes, including T cells, B cells, and dendritic cells (DCs) (n = 3/group). P-values were calculated (relative to an internal control) by means of Student's t-test.

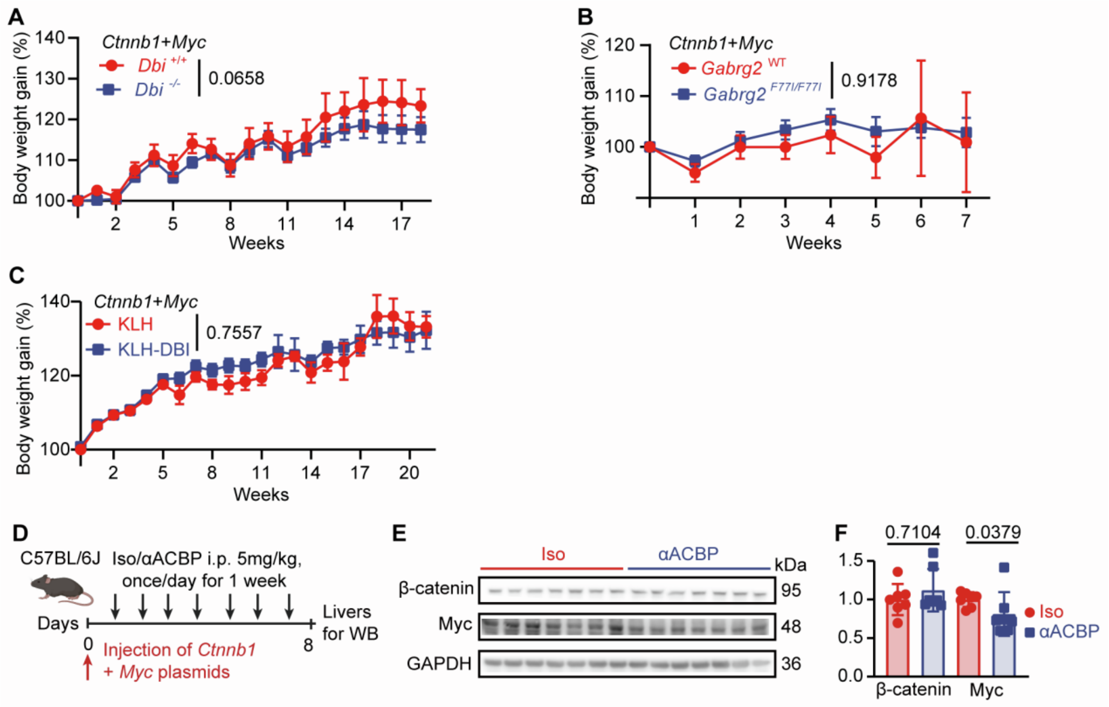

Figure S6 (related to Figure 3). Effects of ACBP/DBI inhibition on body weight changes in *Myc/Ctnnb1*-induced hepatic carcinogenesis mouse models. (A-C) Body weight gain (%) from initial weight throughout the different *in vivo* models shown in Figure 3A, 3E, and 3I (n = 8-12/group). (D) Schematic diagram of ACBP/DBI blockade in *Myc*/*Ctnnb1*-induced hepatic carcinogenesis model. (E-F) Western blots (E) and quantifications (F) of Myc and β-catenin in the whole liver tissues from the mouse model shown in (D) (n = 7/group). *P* values were calculated by two-way ANOVA (A-C) and Mann-Whitney U (F).

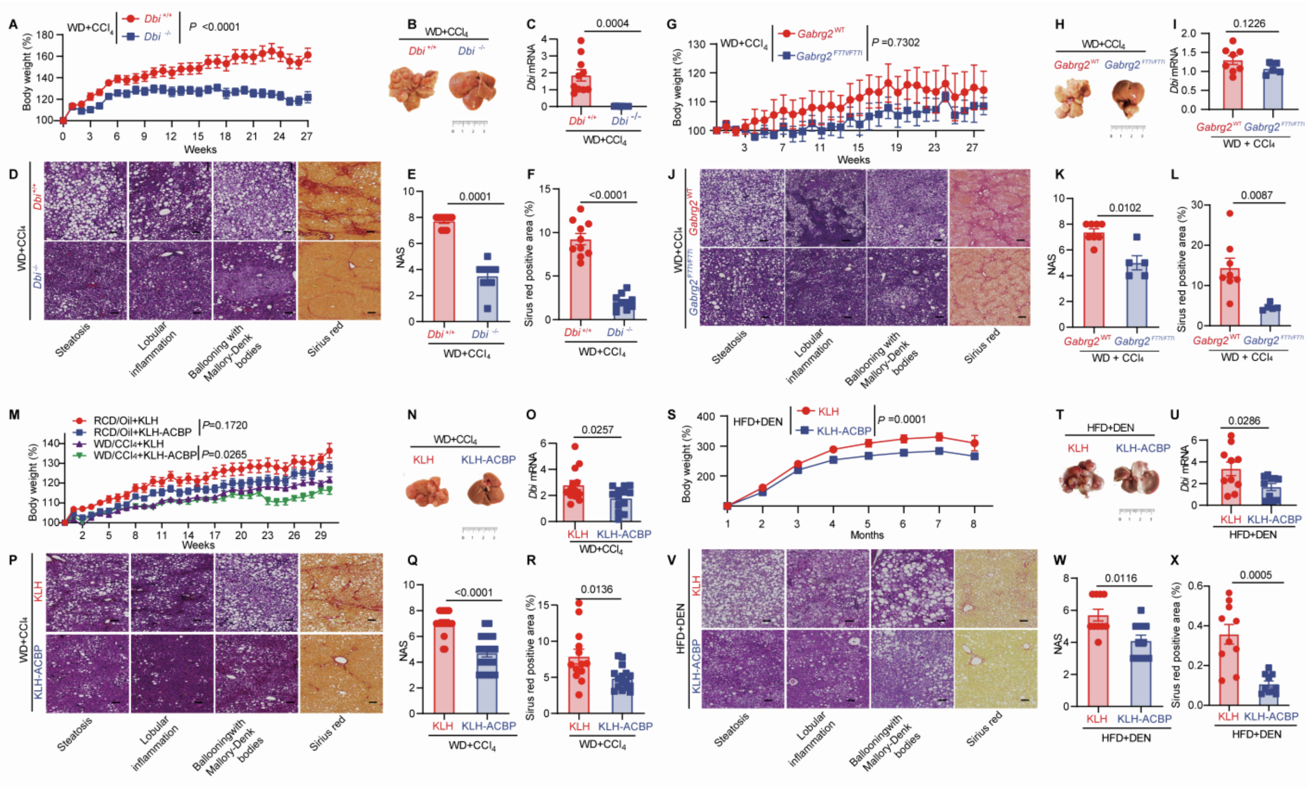

Figure S7 (related to Figure 4). Intra- and extracellular ACBP/DBI inhibition slows MASH/MASH-induced fibrosis. (A, G, M, S) Body weight gain (%) from initial weight throughout the different *in vivo* models (n = 5-27/group). (B, H, N, T) Representative photos of representative livers from each treatment group. (C, I, O, U) *Dbi* mRNA levels in each treatment group (n = 5-13/group). (D, J, P, V) Representative pictures of H&E and Sirius Red staining in each study. Size bar: 200 µm. (E, K, Q, W) NAFLD activity score (NAS) was assessed as the sum of steatosis, lobular inflammation, and ballooning scores for each mouse (n = 5-13/group). (F, L, R, X) Quantification of Sirius Red-positive areas (%) of liver sections in randomly selected liver samples for each treatment group (n = 5-13/group). Error bars represent means ± SEM. *P* values were calculated by two-way ANOVA (A, G, M, S), Welch's t test (C, F, I, R, U, X), T test (L, O), and Mann-Whitney U test (E, K, Q, W).

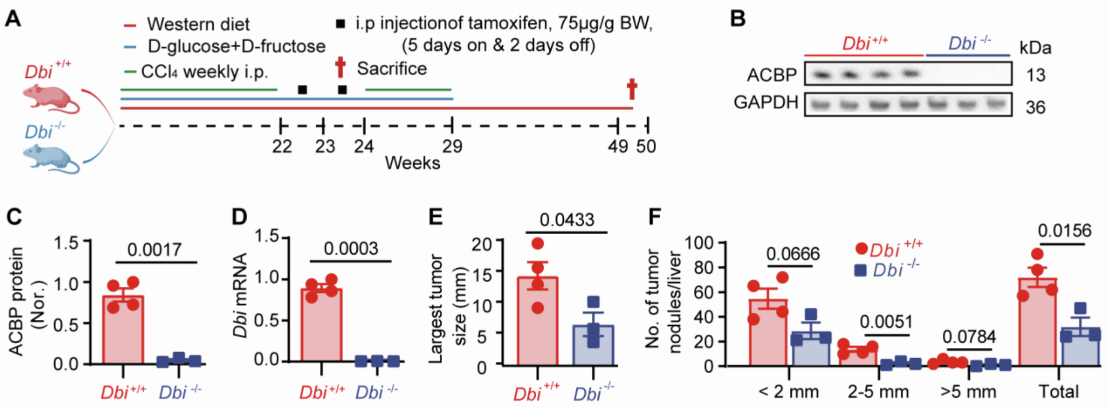

Figure S8 (related to Figure 4). Liver-specific conditional ACBP/DBI knockout suppressed MASH-driven hepatocarcinogenesis. (A) Schematic diagram of hepatic carcinogenesis induced by Western-style diet (WD) plus CCl_4 in tamoxifen-induced liver-specific conditional *Dbi* knockout mice (*Dbi*$^{-/-}$) and control mice (*Dbi*$^{+/+}$). (B) Representative immunoblots detecting ACBP protein in the liver. GAPDH was used as a loading control (n = 3-4/group). (C) Quantification of Western blots (n = 3-4/group). Densitometric ratios of ACBP/GAPDH were normalized to control groups, shown as arbitrary units (Nor.). (D) *Dbi* mRNA levels in each treatment group (n = 3-4/group). (E) Quantification of the largest tumor size of each treatment group (n = 3-4/group). (F) Quantification of the number of tumors with different size (n = 3-4/group). Error bars represent mean ± SEM. *P* values were calculated by Welch's t test (C-E) and multiple t test with Holm-Sidak multiple test correction (F).

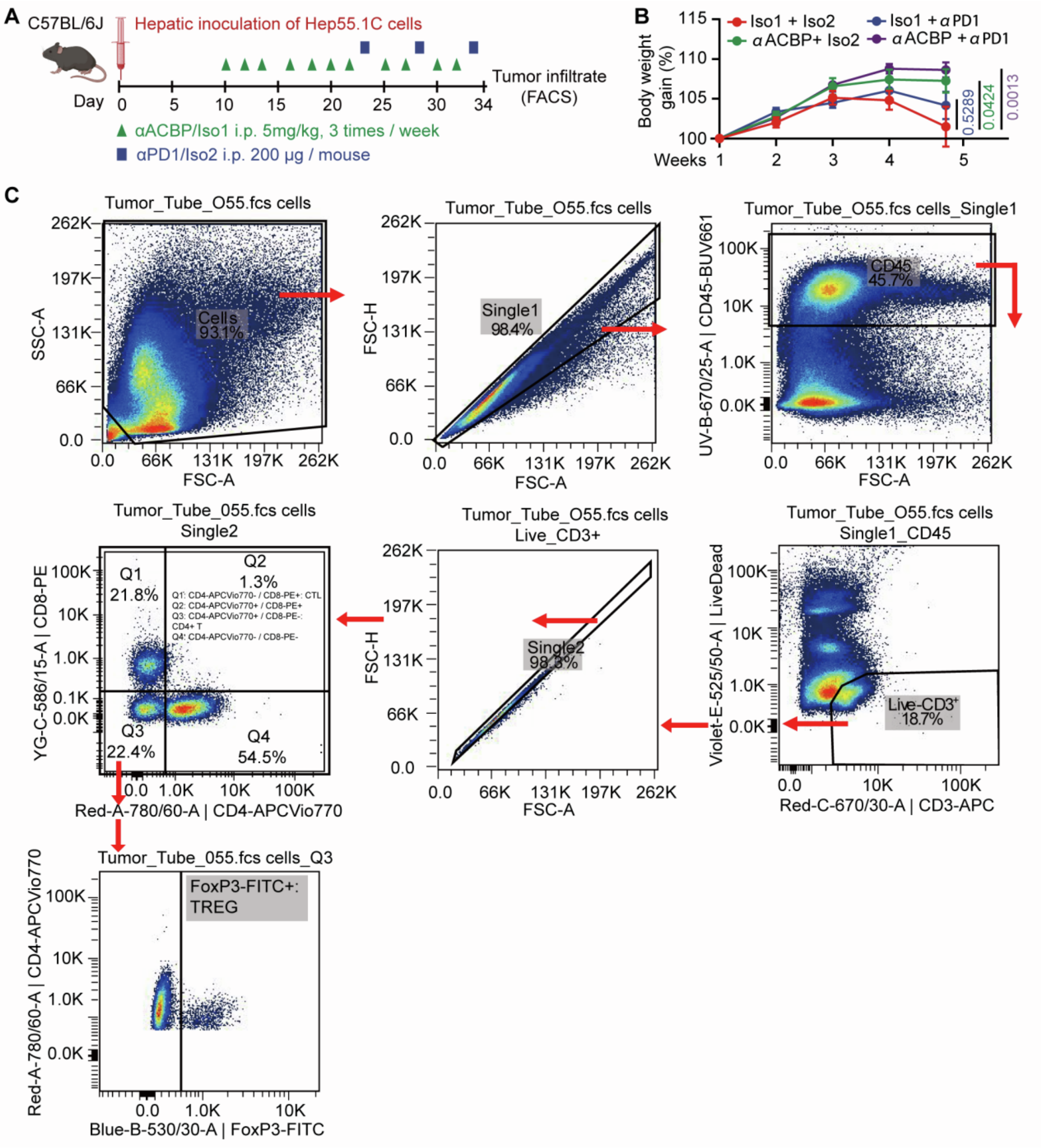

Figure S9 (related to Figure 5). Analysis of the T lymphocyte infiltration in the orthotopic Hep55.1C HCC model treated with anti-ACBP plus anti-PD1. (A) Schematic diagram of experimental flow. (B) Body weight gain (%) from initial weight of the treated mice (n = 9-13/group). *P* values were calculated by two-way ANOVA. (C) Representative immunofluorescence cytometry pictures and gating strategies of T cell infiltrating analyses.

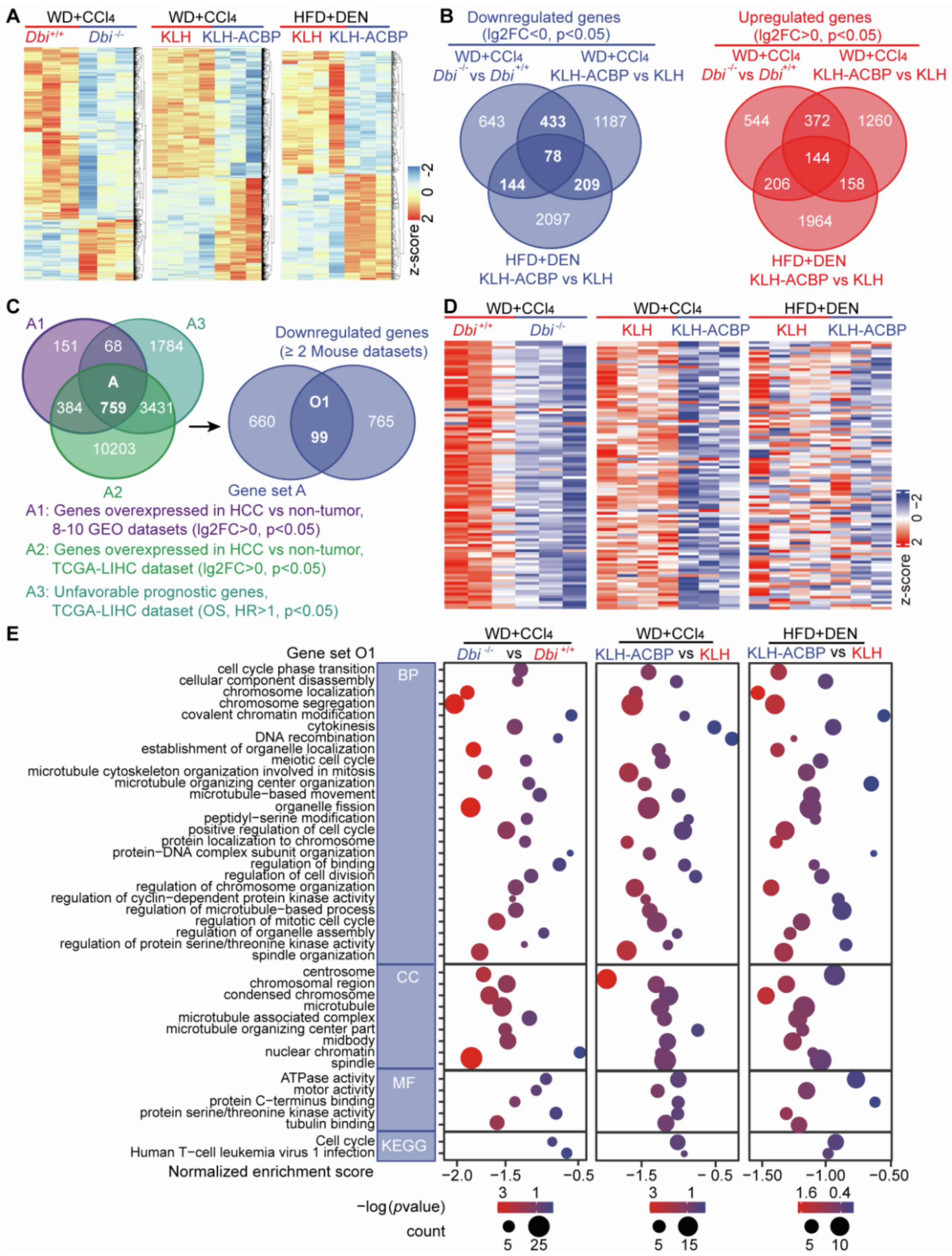

Figure S10 (related to Figure 5). Transcriptomic signatures of ACBP/DBI inhibition in MASH-driven HCC models. (A) Heatmap of the gene expression profiles in three NASH-driven HCC mouse models (n = 3-4/group). (B) Commonly up- or downregulated genes in three mouse models. (C) The flow diagram summarizes the strategy to identify gene set O1. Gene set O1 was defined as genes downregulated by ACBP/DBI inhibition in ≥2 mouse NASH models that are also overexpressed in human HCC, as well as associated with poor prognosis in TCGA-LIHC. (D) Heatmap of gene set O1 in (C) (n = 3-4/group). (E) Gene ontology (GO) and KEGG pathway analysis of gene set O1. Enriched GO-terms for biological process (BP), molecular function (MF), and cellular component (CC) were shown. The pathways were sorted alphabetically in each class. *P* values were calculated using T test (E).

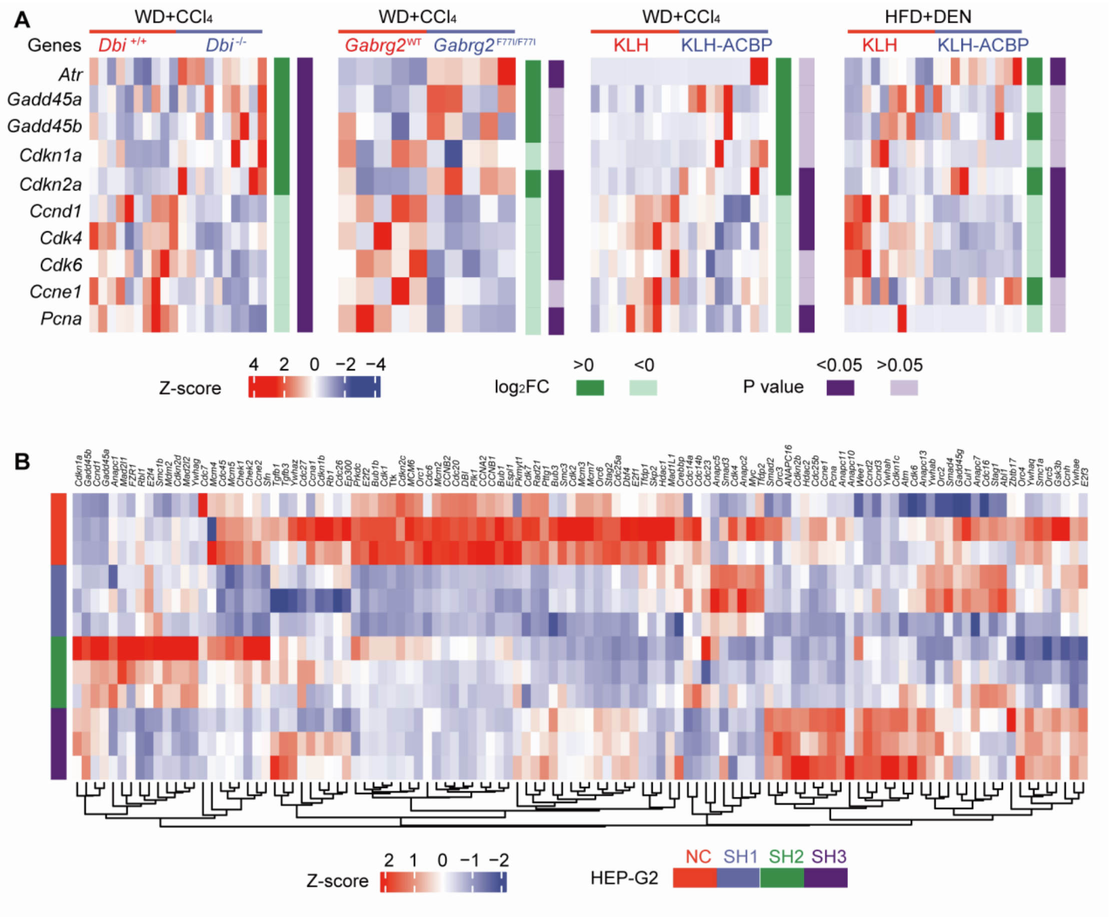

Figure S11 (related to Figure 5). ACBP/DBI inhibition regulates cell cycle-relevant genes. (A) ACBP/DBI inhibition downregulated genes that were positive regulators of cell cycle (*Ccnd1*, *Cdk4*, *Cdk6*, *Ccne1* or *Pcna*) and upregulated genes that inhibit cell cycle progression (*Atr*, *Gadd45a*, *Gadd45b*, *Cdkn1a* or *Cdkn2a*) in four NASH-driven HCC models (n = 5-10/group). *P* values were calculated by T test, Welch's t test, or Mann-Whitney U test, as applicable. (B) Heatmap showing the expression profile of cell cycle-related genes in HEP-G2-derived *DBI* knockdown cell lines (n = 3/group).

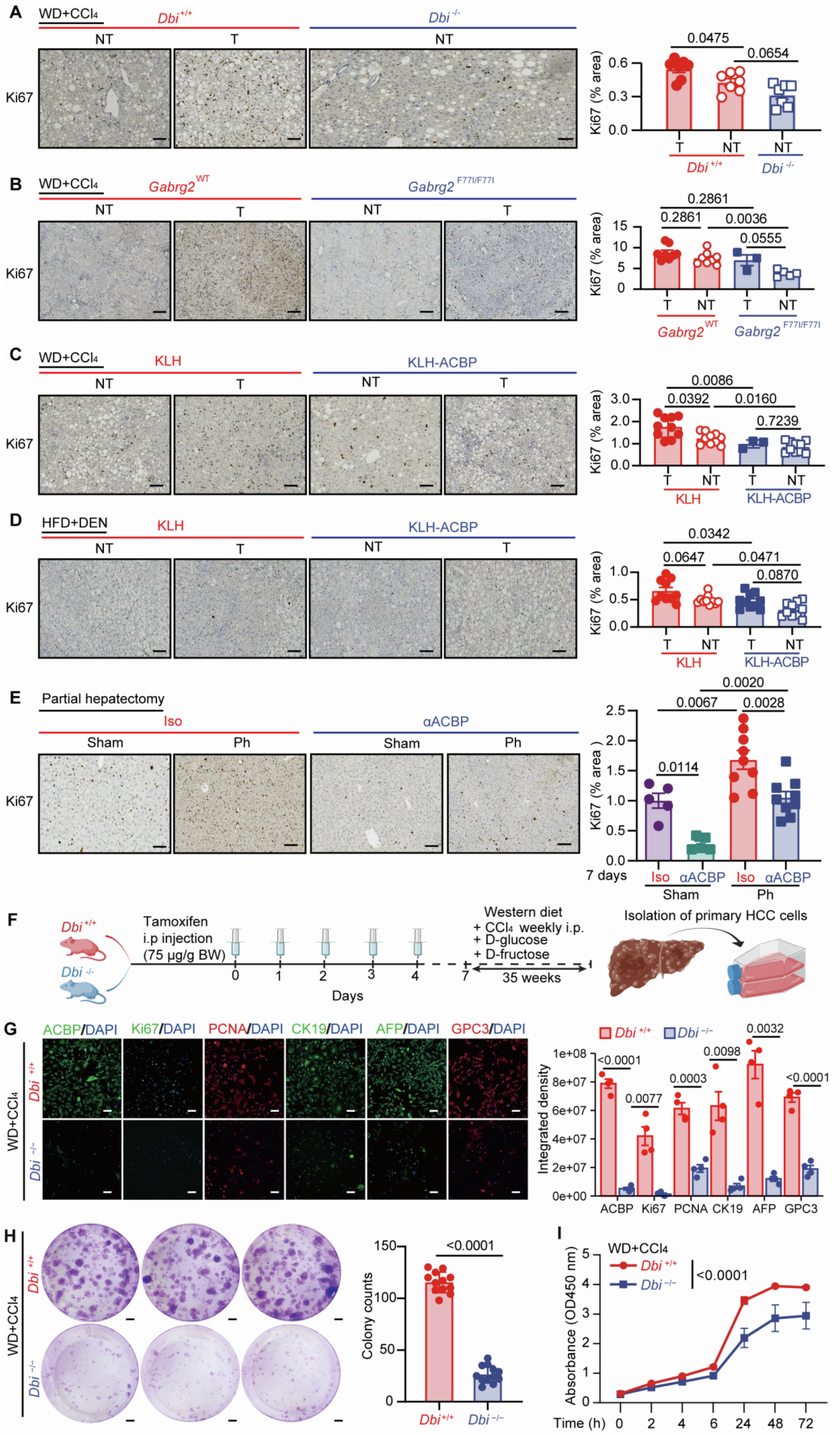
A
WD+CCl4
Dbi +/+
Dbi -/-
NT
T
NT
Ki67
0.0475
0.0654
Ki67 (% area)
0.6
0.3
0.0
T
NT
NT
Dbi +/+
Dbi -/-
B
WD+CCl4
Gabrg2 WT
Gabrg2 F77I/F77I
NT
T
NT
T
Ki67
0.2861
0.2861
0.0036
0.0555
Ki67 (% area)
15
10
5
0
C
WD+CCl4
KLH
KLH-ACBP
0.0086
0.0392
0.0160
0.7239
3.0
2.0
1.0
0.0
D
HFD+DEN
KLH
KLH-ACBP
0.0342
0.0647
0.0471
0.0870
1.0
0.5
0.0
E
Partial hepatectomy
Iso
αACBP
Sham
Ph
0.0020
0.0067
0.0028
0.0114
2.5
2.0
1.5
1.0
0.5
0.0
7 days
Iso
αACBP
Iso
αACBP
Sham
Ph
F
Dbi +/+
Dbi -/-
Tamoxifen
i.p injection
(75 µg/g BW)
0
1
2
3
4
7
Days
Western diet
+ CCl4 weekly i.p.
+ D-glucose
+ D-fructose
35 weeks
Isolation of primary HCC cells
G
ACBP/DAPI
Ki67/DAPI
PCNA/DAPI
CK19/DAPI
AFP/DAPI
GPC3/DAPI
WD+CCl4
Dbi +/+
Dbi -/-
Integrated density
1e+08
8e+07
6e+07
4e+07
2e+07
0e+00
<0.0001
0.0077
0.0003
0.0098
0.0032
<0.0001
ACBP
Ki67
PCNA
CK19
AFP
GPC3
H
WD+CCl4
Dbi +/+
Dbi -/-
Colony counts
150
100
50
0
<0.0001
Dbi+/+
Dbi −/−
I
WD+CCl4
Dbi +/+
Dbi −/−
<0.0001
Absorbance (OD450 nm)
5
4
3
2
1
0
Time (h)
0
2
4
6
24
48
72

Figure S12 (related to Figure 5). ACBP/DBI inhibition decreased cell proliferation. (A-E) Representative images and quantification of Ki67 IHC staining in liver sections from distinct mouse models (n = 5-10/group). T/NT represents Tumor/Non-tumor. Scale bars represent 200 µm. (F) Schematic diagram of the strategy to generate primary *Dbi*$^{+/+}$ and *Dbi*$^{-/-}$ HCC cells in the WD+CCl_4-induced mouse model. (G) Representative images of ACBP, Ki67, PCNA, CK19, AFP and GPC3 immunofluorescence staining in primary *Dbi*$^{+/+}$ and *Dbi*$^{-/-}$ HCC cells and quantification (n = 4/group). Scale bars represent 100 µm. (H) Representative images and quantification of colony formation of primary *Dbi*$^{+/+}$ and *Dbi*$^{-/-}$ HCC cells (n = 12/group). Scale bar represents 3.5 mm. (I) CCK-8 proliferation assay in HCC primary cells. *In vitro* experiments were repeated at least three times (n = 6/group). Error bars represent means ± SEM. *P* values were calculated by one-way ANOVA (A, B, D, E), Welch's one-way ANOVA with Games-Howell test (C), Welch's t test (G), T test (H), and two-way ANOVA (I).

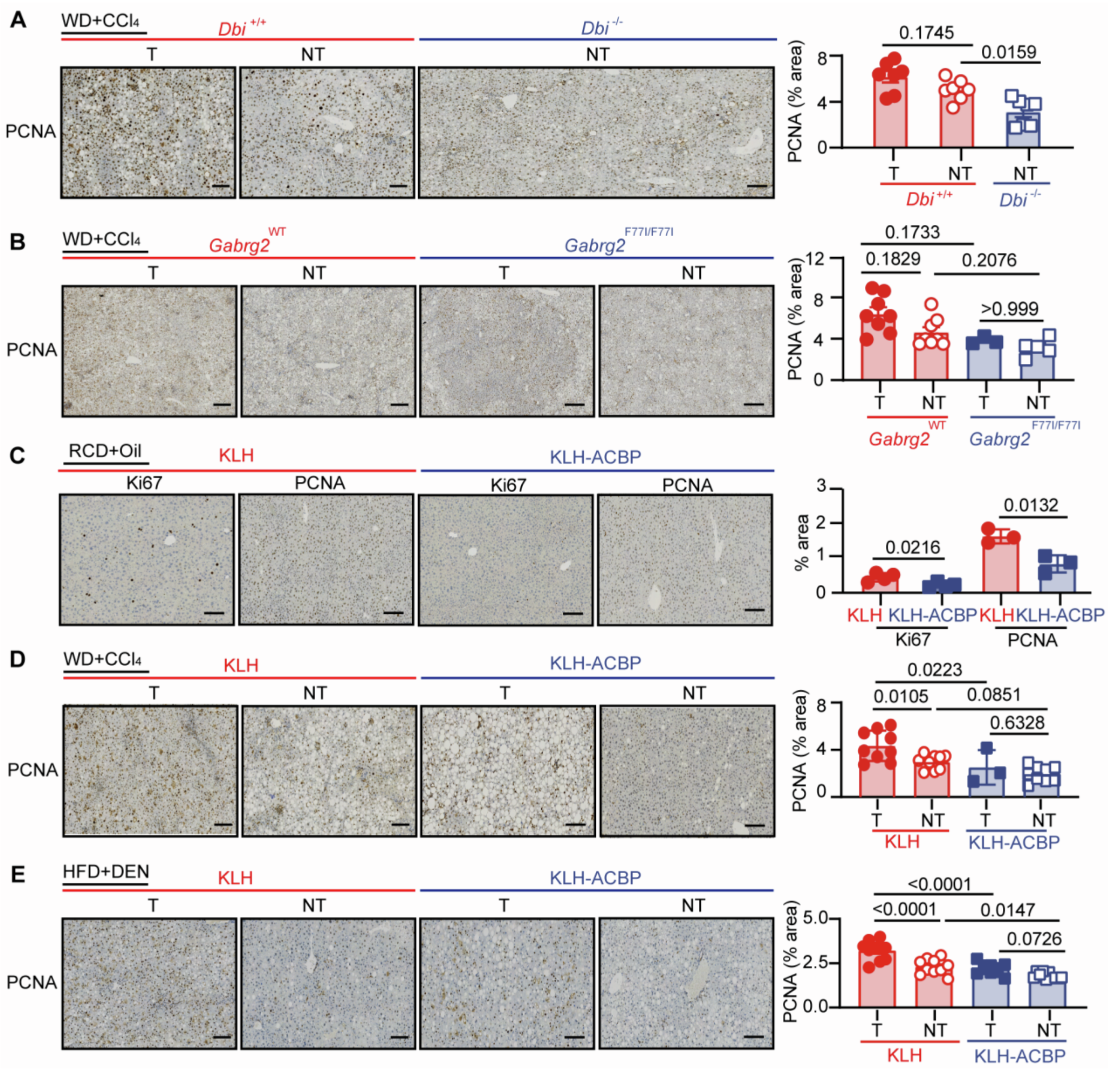

Figure S13 (related to Figures 4 & 5). ACBP/DBI inhibition attenuated cell proliferation. (A-E) Representative images and quantification of PCNA/Ki67 immuno-staining in liver sections from distinct mouse models (n = 3-10/group). Scale bars represent 200 µm. Error bars represent means ± SEM. *P* values were calculated by one-way ANOVA post hoc Tukey HSD test (A), Kruskal-Wallis test with Dunn's multiple comparison (B), T test (C), and One-way ANOVA with Dunnett's multiple comparison test (D, E).

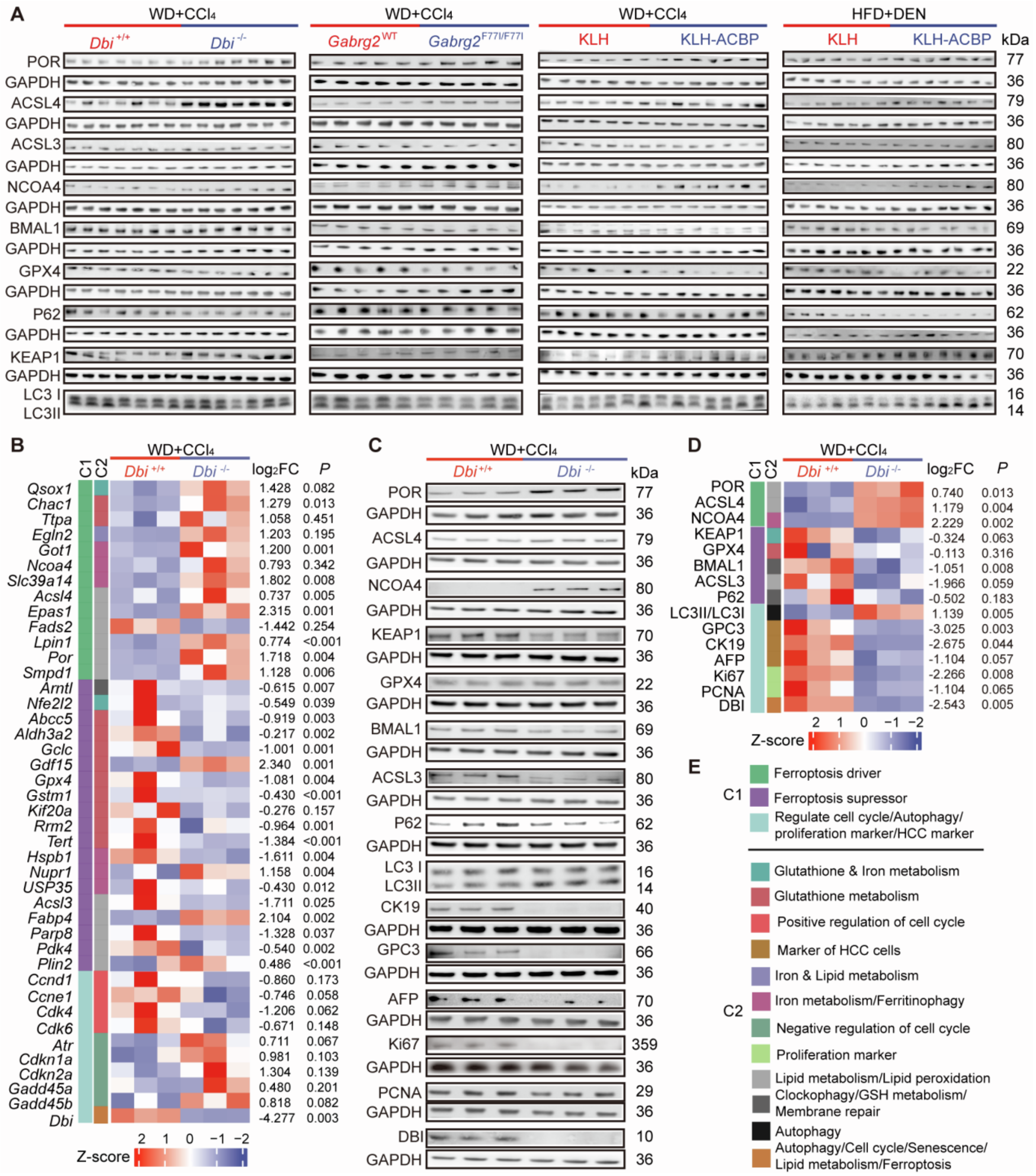

Figure S14 (related to Figures 4, 5 & 6). ACBP/DBI knockout regulate ferroptosis gene/protein signature in primary HCC cells. (A) Western blots of ferroptosis protein signature in liver tissues from different NASH-HCC mouse models (n = 5-7/group). (B) qRT-PCR analysis (n = 3/group). Ferroptosis and cell cycle-related gene expression profiles in primary *Dbi*$^{+/+}$ and *Dbi*$^{-/-}$ HCC cells were shown in a heatmap. (C-D) Immunoblotting and densitometric analysis of the blots (n = 3/group). Expression of ferroptosis-related proteins, malignancy-linked markers and proliferation markers was determined by immunoblotting in primary *Dbi*$^{+/+}$ and *Dbi*$^{-/-}$ HCC cells. The normalized values were shown in a heatmap. (E) Functional annotations of genes/proteins in (B-D). *P* values were calculated by T test, Welch's t test, or Mann-Whitney U test (B, D).

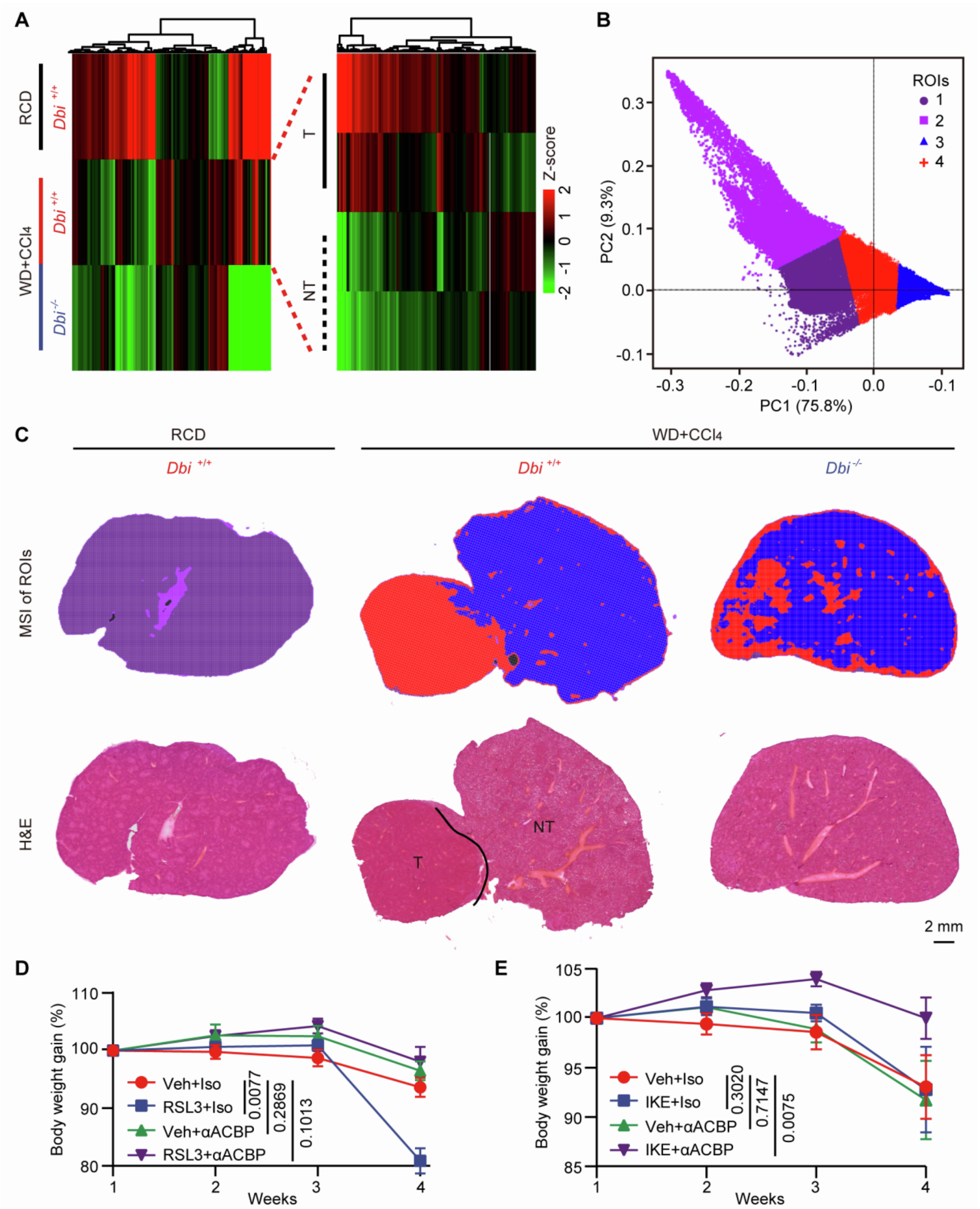

Figure S15 (related to Figures 6 & 7). Spatial metabolomic landscape of liver sections from WD/CCl4-induced HCC models. (A) Heatmap depicting the average metabolite intensities across three representative liver lobes. The left part of the heatmap displays metabolite signatures in liver sections from *Dbi*$^{+/+}$-RCD, *Dbi*$^{+/+}$-WD/CCl_4, and *Dbi*$^{-/-}$-WD/CCl_4 mice (n = 1/group). Rows are mean of the replicates per condition. The right part of the heatmap represents the metabolite signatures of tumor (T) and non-tumor (NT) liver sections of *Dbi*$^{+/+}$-WD/CCl_4 mice. Rows indicate means of the replicates per condition (left) after integration of all data points and columns are putative metabolites. Hierarchal clustering of the metabolite intensities was performed based on Euclidean distance. Color scale (red/green) represents normalized metabolite intensities (log2), and the missing values are shown in grey. (B) Principal component analysis (PCA) of the mass spectrometry imaging

(MSI) data from regions of interest (ROI) of the aforementioned three liver lobes (as defined in C). All replicates from the representative liver lobes were used. Colors represent Kmeans clustering of pixels (metabolites intensities) in liver section. (C) Representative pictures of negative polarity MSI and H&E staining of livers. The color code corresponds to that in (B). (D-E) Body weight gain (%) from initial weight of the treated mice as seen from Figure 7E and Figure 7K (n = 9/group). *P* values were calculated by two-way ANOVA.

Supplemental Table

Table S1. Genes used for calculating Immunosuppression and Immunostimulation scores (related to Figure 6E).

Immunosuppression score	Immunostimulation score
Ptprc	Cd27
Cd3g	Cd276
CD8a	Cd28
Cd4	Cd40
PDCD1	Cd40lg
Lag3	Cd48
Tigit	Cd70
Tnfrsf18	Cd80
Vsir	Cd86
Havcr2	Cxcl12
Foxp3	Cxcr4
	Entpd1
	Icos
	Icosl
	Il2ra
	Il6
	Il6ra
	Klrc1
	Lta
	Mill2
	Nt5e
	Tmem173
	Tnfrsf13b
	Tnfrsf13c
	Tnfrsf14
	Tnfrsf17
	Tnfrsf18
	Tnfrsf25
	Tnfrsf4
	Tnfrsf8
	Tnfrsf9
	Tnfsf13
	Tnfsf13b
	Tnfsf14
	Tnfsf15
	Tnfsf18
	Tnfsf4
	Ulbp1
