# Supplementary material for: Neutralization of acyl coenzyme A binding protein for the experimental prevention and treatment of hepatocellular carcinoma
Source: Cell Rep Med. 2025 Jul 7;6(7):102232. doi: 10.1016/j.xcrm.2025.102232 (PMC12281431; doi:10.1016/j.xcrm.2025.102232)
Supplement: Document S2. Article plus supplemental information [file mmc3.pdf]

Cell Reports
Medicine

Article

Neutralization of acyl coenzyme A binding protein for the experimental prevention and treatment of hepatocellular carcinoma

Graphical abstract

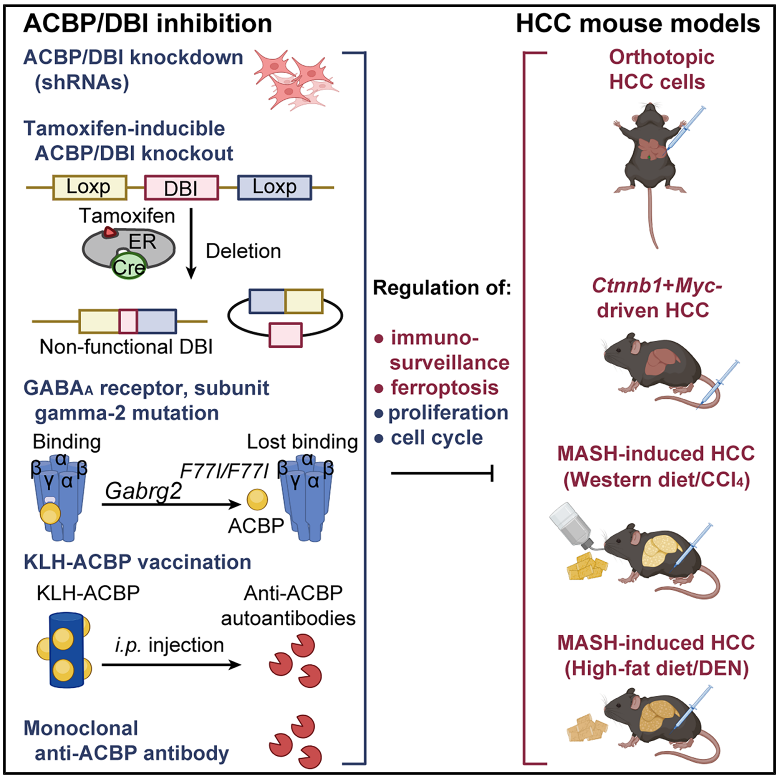

Highlights

- ACBP/DBI contributes to the pathogenesis of human HCC
- ACBP/DBI neutralization inhibits HCC proliferation in mice
- ACBP/DBI neutralization sensitizes hepatocytes and HCC cells to ferroptosis
- ACBP/DBI neutralization enhances cancer immunosurveillance

Authors

Sijing Li, Omar Motiño, Flavia Lambertucci, ..., Maria Chiara Maiuri, Jessica Zucman-Rossi, Guido Kroemer

Correspondence

maria-chiara.maiuri@inserm.fr (M.C.M.), kroemer@orange.fr (G.K.)

In brief

Sijing Li et al. demonstrate that ACBP/DBI expression is upregulated in human HCC. Elevated ACBP/DBI mRNA associates with poor prognosis in patients with HCC. ACBP/DBI neutralization attenuates HCC development and progression across distinct preclinical models. Anti-ACBP/DBI monoclonal antibody sensitizes malignant hepatocytes to ferroptosis and enhances HCC responses to immune checkpoint blockade.

Li et al., 2025, Cell Reports Medicine *6*, 102232
July 15, 2025 © 2025 The Author(s). Published by Elsevier Inc.
https://doi.org/10.1016/j.xcrm.2025.102232

CellPress

Cell Reports Medicine

CellPress
OPEN ACCESS

Article

Neutralization of acyl coenzyme A binding protein for the experimental prevention and treatment of hepatocellular carcinoma

Sijing Li,[1,2,3,4] Omar Motiño,[1,2,3,22] Flavia Lambertucci,[1,2,3,22] Jonathan Pol,[1,2,3] Hui Chen,[1,2,3] Long Pan,[5] Sylvère Durand,[1,2,3] Federica Rossin,[6,7] Claudia Campani,[5,8] Lucie Poupel,[9] Christophe Klein,[9] Léa Montégut,[1,2,3] María Pérez-Lanzón,[1,2,3] Gerasimos Anagnostopoulos,[1,2,3] Uxia Nogueira-Recalde,[1,2,3,10] Alexandra Cerone,[1,2,3] Fanny Aprahamian,[1,2,3] Yanbing Dong,[1,2,3,4] Manuela Lizarralde-Guerrero,[1,2,3,4] Enfu Xue,[1,2,3,4] Peng Liu,[1,2,3] Liwei Zhao,[1,2,3] Hui Pan,[1,2,3] Vincent Carbonnier,[1,2,3] Sylvie Lachkar,[1,2,3] Ester Gloria Saavedra Díaz,[1,2,3,11] Li Sun,[12] Chantal Desdouets,[13] Sabine Colnot,[14] Oliver Kepp,[1,2,3] Isabelle Martins,[1,2,3] Laurence Zitvogel,[15,16,17,18] Mauro Piacentini,[6,7] Jean-Charles Nault,[5,20] Maria Chiara Maiuri,[1,2,19,*] Jessica Zucman-Rossi,[5,21] and Guido Kroemer[1,2,3,21,23,*]

[1]Université Paris Cité, Sorbonne Université, Inserm U1138, Centre de Recherche des Cordeliers, F-75006 Paris, France
[2]Équipe Labellisée Par La Ligue Contre le Cancer, Institut Universitaire de France, Paris, France
[3]Université Paris-Saclay, INSERM US23 / CNRS UAR 3655, Metabolomics and Cell Biology Platforms, Institut Gustave Roussy, 94805 Villejuif, France
[4]Faculté de Médecine, Université de Paris Saclay, Kremlin Bicêtre, Paris, France
[5]Team "Functional Genomics of Solid Tumors", Centre de Recherche des Cordeliers, Inserm U1138, Université Paris Cité, Sorbonne Université, 75006 Paris, France
[6]Department of Biology, University of Rome 'Tor Vergata', Rome, Italy
[7]National Institute for Infectious Diseases IRCCS "Lazzaro Spallanzani", 00133 Rome, Italy
[8]Department of Experimental and Clinical Medicine, Internal Medicine and Hepatology Unit, University of Firenze, Florence, Italy
[9]CHICS, Centre de Recherche des Cordeliers, Inserm U1138, Université Paris Cité, Sorbonne Université, Paris, France
[10]Grupo de Investigación en Reumatología (GIR), Instituto de Investigación Biomédica de A Coruña (INIBIC), Fundación Profesor Novoa Santos, A Coruña, Spain
[11]Departamento de Bioquímica y Biología Molecular, Fisiología, Genética e Inmunología, Instituto Universitario de Investigaciones Biomédicas y Sanitarias (IUIBS), Universidad de Las Palmas de Gran Canaria, Las Palmas de Gran Canaria, Spain
[12]Jiangsu Key Laboratory of Drug Screening, China Pharmaceutical University, Nanjing, China
[13]Team Genomic Instability, Metabolism, Immunity and Liver Tumorigenesis Laboratory, Equipe Labellisée LIGUE 2023, Centre de Recherche des Cordeliers, Sorbonne Université, INSERM, Université de Paris, Paris, France
[14]Team "Oncogenic functions of β-catenin Signaling in the Liver (ONCOLIV)", Centre de Recherche des Cordeliers, Equipe Labellisée Par La Ligue Contre le Cancer, Inserm U1138, Université Paris Cité, Sorbonne Université, Paris, France
[15]Gustave Roussy Cancer Center, ClinicoBiome, 94805 Villejuif, France
[16]Université Paris Saclay, Faculty of Medicine, 94270 Kremlin Bicêtre, France
[17]Inserm U1015, Equipe Labellisée Par La Ligue Contre le Cancer, 94800 Villejuif, France
[18]Center of Clinical Investigations in Biotherapies of Cancer (CICBT), Gustave Roussy, 94805 Villejuif, France
[19]Department of Molecular Medicine and Medical Biotechnologies, University of Napoli Federico II, 80131 Naples, Italy
[20]Liver Unit, Avicenne Hospital, Paris-Seine-Saint-Denis Universitary Hospitals, AP-HP, Bobigny, France
[21]Institut du Cancer Paris CARPEM, Department of Biology, Hôpital Européen Georges Pompidou, AP-HP, Paris, France
[22]These authors contributed equally
[23]Lead contact
*Correspondence: maria-chiara.maiuri@inserm.fr (M.C.M.), kroemer@orange.fr (G.K.)
https://doi.org/10.1016/j.xcrm.2025.102232

SUMMARY

Acyl coenzyme A binding protein (ACBP encoded by *diazepam binding inhibitor DBI*) is involved in non-malignant liver diseases. Here, we show that *DBI* mRNA and circulating ACBP/DBI levels are increased in patients with hepatocellular carcinoma (HCC). We investigated its role in hepatocarcinogenesis in mice, inhibiting ACBP/DBI by three methods: (1) inducible whole-body or liver-specific knockout of *DBI*, (2) a point mutation of the ACBP/DBI receptor (*GABRG2*), and (3) induction of autoantibodies neutralizing ACBP/DBI. ACBP/DBI plays a major pro-carcinogenic role in HCC induced by intrahepatic transplantation of HCC cell lines, transgenic co-expression of the two oncogenes *Myc* and *Ctnnb1*, and chronic challenge with a Western-style diet together with either carbon tetrachloride (CCl_4) or diethylnitrosamine. ACBP/DBI inhibition normalizes HCC-associated gene expression, reducing oncogenic alterations in cell cycle-, immunomodulatory-, and ferroptosis-regulatory genes. ACBP/DBI inhibition increases HCC responses to

This is an open access article under the CC BY license (http://creativecommons.org/licenses/by/4.0/).

PD-1 blockade and sensitizes HCC to the therapeutic induction of ferroptosis. Hence, ACBP/DBI constitutes an actionable target involved in HCC pathogenesis.

INTRODUCTION

Hepatocellular carcinoma (HCC), the third leading cause of cancer-related deaths worldwide,[1] is linked to a series of established risk factors that include metabolic dysregulation (such as obesity and diabetes favoring the development of metabolic dysfunction-associated steatohepatitis [MASH] and progression to advanced fibrosis and cirrhosis), repeated insult by hepatotoxins (such as alcohol and aflatoxin), as well as infection by hepatotropic viruses (such as hepatitis viruses B and C).[2] In spite of the ever more detailed comprehension of the molecular and cellular pathways leading to HCC development,[2–5] there are rather few successful treatment options. Curative attempts (resection, thermal ablation, and transplantation) are usually limited to patients with early-stage HCC (BCLC-0 and BCLC-A), while transarterial chemoembolization or radioembolization and systemic treatments (tyrosine-kinase inhibitors and immunotherapy) are usually reserved for intermediate- (BCLC-B) and advanced-stage (BCLC-C) patients.[6,7]

Acyl coenzyme A (CoA) binding protein (ACBP) is a small (87 amino acids for the dominant isoform) protein encoded by *diazepam binding inhibitor* (*DBI*) (Montégut et al., 2023). ACBP/DBI is expressed by all nucleated cell types but is particularly abundant in hepatocytes.[7,8] In the cytoplasm of cells, ACBP/DBI interacts with various lipids including medium-chain acyl CoA esters and contributes to metabolic homeostasis.[9,10] ACBP/DBI can be released from autophagic, stressed, and dying cells through a nonconventional pathway[8,11] and acts on a particular subunit of the γ-aminobutyric acid type A receptor ($GABA_AR$), namely GABRG2,[12] to mediate a series of effects that include (1) inhibition of autophagy, (2) lipo-anabolic reactions favoring lipid accumulation in cells, and (3) stimulation of food intake.[8,13] Hence, inhibition of ACBP/DBI by its inducible knockout, mutation of its receptor GABRG2 on a specific residue (F77I), or antibody-mediated neutralization of the extracellular ACBP/DBI pool has a triple consequence: (1) stimulation of autophagy, (2) lipo-catabolic reactions favoring lipolysis and fatty acid oxidation, and (3) inhibition of food intake.[8,13]

All three methods of ACBP/DBI inhibition also have marked organ-protective effects that are mechanistically linked to the induction of macroautophagy (to which we refer as "autophagy").[14] Thus, ACBP/DBI inhibition protects the heart against ischemia-reperfusion damage and anthracycline toxicity, the lung against bleomycin-induced inflammation and fibrosis, and has marked hepatoprotective effects as well.[14,15] In the liver, ACBP/DBI neutralization prevents excessive cell death, inflammation, and fibrosis in response to a broad array of insults. Such hepatic insults can be mechanical (e.g., transient arterial occlusion, and bile duct ligation), chemical (e.g., acetaminophen, carbon tetrachloride [CCl_4], concanavalin A, and rosiglitazone), and dietary (e.g., high-fat or methionine/choline-deficient diets),[13,14] underscoring the broad contribution of ACBP/DBI to hepatic pathogenesis.

The implications of ACBP/DBI in malignant disease are poorly studied. ACBP/DBI is upregulated in breast cancer and non-small cell lung cancers (NSCLCs), associated with poor patient prognosis.[16,17] *In vitro*, knockdown of ACBP/DBI mitigated NSCLC cell proliferation.[16] In glioma and glioblastoma, ACBP/DBI is strongly overexpressed and stimulates fatty acid oxidation through cell-autonomous effects relying on its interaction with acyl CoA esters. Its knockdown inhibits proliferation and triggers glioblastoma cell senescence as well.[18] Immunohistochemical detection of ACBP/DBI also reveals its overexpression in cholangiocarcinoma.[19] However, there is no knowledge on the role of ACBP/DBI in HCC.

Prompted by these premises, we investigated the role of ACBP/DBI in hepatic carcinogenesis. Using a compendium of methods to inhibit ACBP/DBI (by its knockout, receptor mutation, or antibody-mediated neutralization), we demonstrate the critical implication of ACBP/DBI in hepatocarcinogenesis and tumor progression. Of note, high intratumoral ACBP/DBI mRNA expression and high levels of circulating ACBP/DBI protein levels are associated with features of poor prognosis in patients with HCC, supporting the translational relevance of our preclinical findings.

RESULTS

Increased ACBP/DBI levels in human HCC

In 9 different publicly available gene expression datasets including The Cancer Genome Atlas (TCGA), HCC tumors consistently contain higher *DBI* mRNA levels than the normal adjacent liver tissue (Figures 1A and S1A–S1I). According to TCGA, this *DBI* elevation occurred independently from each of the components of the tumor/node/metastasis (TNM) staging system, Child-Pugh grade, tumor differentiation, vascular invasion, as well as levels of fibrosis and inflammation (Figures 1B, 1C, and S1J–S1Q). However, *DBI* mRNA was particularly abundant in patients with alpha-fetoprotein (AFP) protein levels >400 ng/mL (Figure 1D), and *DBI* mRNA levels above the median values were associated with shorter overall survival in TCGA and in an independent HCC patient cohort (Figures 1E and S1R). Of note, we detected a significant positive correlation of *DBI* mRNA with the transcription of multiple HCC-relevant oncogenes across 34 publicly available datasets. This analysis also revealed a negative correlation between the tumor suppressor *TPR53* (best known as p53) and *DBI* mRNAs (Figure S2), in accord with prior observations.[20]

Using ELISA, we measured ACBP/DBI protein concentrations in the plasma of 146 patients with HCC belonging to different BCLC stages and 58 plasmas of patients with chronic liver diseases without any history of HCC. We found that ELISA-detectable plasma ACBP/DBI levels (ng/mL) were higher in patients with HCC compared with the group without any current or prior HCC (Figure 1F), correlating with poor prognostic features such as advanced BCLC stage (Figure 1G), vascular invasion

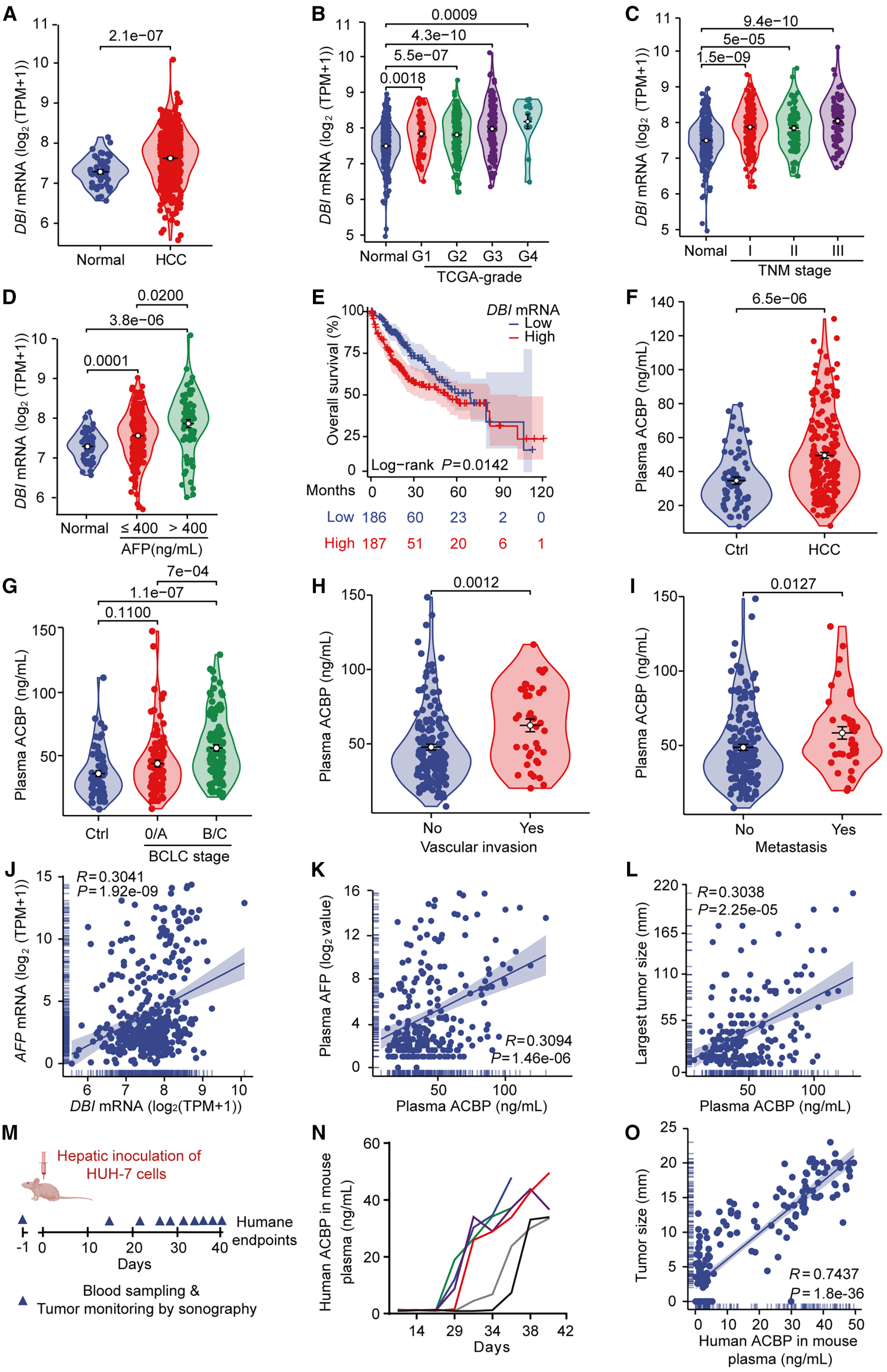

(legend on next page)

(Figure 1H), and extrahepatic metastasis (Figure 1I). In addition, *DBI* mRNA correlated with *AFP* mRNA in liver biopsies (Figure 1J), echoing a correlation between plasma protein levels of ACBP/DBI and AFP (Figure 1K). Moreover, ACBP/DBI plasma concentrations correlated with tumor size, as detected by CT scans (Figure 1L). Altogether, these results support the idea that advanced HCC is associated with high ACBP/DBI levels.

Importantly, when human HCC cells were orthotopically inoculated into the livers from immunodeficient mice (Figure 1M), human but not mouse ACBP/DBI plasma concentrations measured with species-specific ELISAs (that distinguish human and mouse ACBP/DBI)[21] increased over time, correlating with the size of the tumors (Figures 1N, 1O, and S3). These findings indicate that raising circulating ACBP/DBI may directly stem from HCC.

Altogether, these findings suggest that human HCC progression is associated with an increase in ACBP/DBI expression by malignant tissues as well as by an increase in ACBP/DBI plasma levels.

Intra- and extracellular ACBP/DBI contribute to the pathogenesis of transplantable, oncogene-, and carcinogen-induced HCC

Among a collection of distinct human HCC cell lines, HUH-7 cells express the highest ACBP/DBI mRNA and protein levels (Figure S4A). Knockdown of *DBI* with three different short hairpin RNAs (shRNAs) yielded HUH-7 clones that exhibited less proliferation and clonogenic potential, as well as a reduced proportion of cells in the S-phase of the cell cycle. Similar results were obtained for human hepatoblastoma HEP-G2 cells and mouse HCC Hep55.1C cells subjected to the knockdown of *DBI* and *Dbi*, respectively (Figures S4B–S4M). Of note, both HEP-G2 cells and Hep55.1C express the ACBP/DBI receptor GABRG2,[22,23] as do freshly isolated mouse hepatocytes,[22] Kupfer cells, and several immune cell types including B and T lymphocytes, as well as dendritic cells (Figure S5).

As compared to the parental cell line, three Hep55.1C clones subjected to *DBI* depletion (by three distinct shRNAs) were relatively poorly pathogenic when inoculated orthotopically into the livers of immunocompetent C57BL/6 mice (Figure 2A), as indicated by reduced mortality of recipient mice (Figure 2B), a diminished number of mice developing macroscopic HCC (Figure 2C), as well as a decreased number and total weight of HCC nodules at endpoint (Figures 2D and 2E). Constant monitoring of tumor growth using luciferase-transduced Hep55.1C clones confirmed the failure of *Dbi*-depleted cells to develop HCC (Figures 2F–2K). Of note, neutralization of extracellular ACBP/DBI protein in mouse plasma (Figure 2M) by means of a suitable auto-vaccination protocol (in which autoantibodies against ACBP/DBI were induced by adjuvanted inoculation of ACBP/DBI conjugated to the immunogen keyhole limpet hemocyanin [KLH])[24] also delayed the orthotopic growth of parental (ACBP/DBI-expressing) luciferase-transduced Hep55.1C cancers as compared to control animals immunized with KLH alone (Figures 2L–2R).

We next resorted to a model of oncogene-induced hepatocarcinogenesis in which two plasmids coding for *Myc* and *Ctnnb1* were co-transfected into hepatocytes by hydrodynamic injection. Mice in which a floxed version of *DBI* ($DBI^{f/f}$) was ubiquitously excised by a tamoxifen-inducible Cre recombinase (*UBCcre/ERT2*) (genotype: *UBCcre/ERT2::*$DBI^{f/f}$, $Dbi^{+/+}$, control: $DBI^{f/f}$ without *CRE*, $Dbi^{-/-}$) were rather resistant to *Myc/Ctnnb1*-induced hepatic carcinogenesis (Figures 3A–3D and S6A). Moreover, mice in which the γ2 subunit of GABAA receptor (*Gabrg2*) was homozygously mutated (F77I) (genotype: $Gabrg2^{F77I/F77I}$), causing a loss of interaction with ACBP/DBI,[24] were relatively resistant to *Myc/Ctnnb1*-induced HCC as well (Figures 3E–3H and S6B). Finally, immunization with KLH-ACBP/Dbi conferred partial protection against *Myc/Ctnnb1*-mediated hepatocellular carcinogenesis (Figures 3I–3L and S6C). Of note, neutralization of ACBP/DBI did not affect *Ctnnb1* expression in the mouse liver but did reduce that of *Myc* (Figures S6D–S6F).

We previously demonstrated the contribution of ACBP/DBI to diet and toxin-induced hepatosteatosis and fibrosis.[12] However, the possible implication of ACBP/DBI in diet- and toxin-induced HCC is elusive. For this reason, we examined a model of hepatic carcinogenesis induced by a Western-style diet (WD) combined with weekly intraperitoneal (i.p.) injections of the hepatotoxin CCl_4.[25] We observed that knockout of *Dbi* (Figures 4A–4F and S7A–S7F), homozygous *Gabrg2* mutation (Figures 4G–4L and S7G–S7L), as well as immunization with KLH-ACBP/Dbi (Figures 4M–4R and S7M–S7R) mitigated the stigmata of WD + CCl_4-induced liver damage (MASH, lobular inflammation, ballooning with Mallory-Denk bodies, and fibrosis), reduced liver

Figure 1. Overabundance of ACBP/DBI in patients with HCC

(A–E) Analysis of data from The Cancer Genome Atlas (TCGA) dealing with liver hepatocellular carcinoma (HCC or LIHC). (A) Higher expression of *DBI* mRNA in HCC tissues versus normal tissues (n = 50–374/group). (B and C) *DBI* mRNA levels are increased in HCC tissues compared with normal tissues independent of tumor grades and TNM stages in TCGA (n = 12–276/group). (D) Elevated *DBI* mRNA levels are detected in tumor tissues in HCC patients with an AFP >400 ng/mL compared with AFP ≤400 ng/mL (n = 50–221/group). (E) High *DBI* expression is associated with poor prognosis of patients with HCC (n = 186–187/group).

(F–L) Analysis of data from the AP-HP cohort. (F) ACBP plasma levels are increased in patients with HCC compared to controls (n = 64–193/group). (G–I) Elevated ACBP plasma levels are associated with advanced BCLC stages, vascular invasion, and tumor metastasis (n = 37–159/group). (J and K) Positive correlation between ACBP/DBI and AFP at both mRNA (n = 374) and protein levels (n = 233). (L) Plasma ACBP levels are positively associated with the largest tumor size in patients with HCC (n = 188).

(M–O) Tumor origin of ACBP/DBI in mice bearing human HCC.

(M) Schematic diagram of orthotopic liver transplantation of HUH-7 cells in nude mice. HUH-7 tumors were monitored by sonography (for representative images, see Figure S2), while blood samples were simultaneously collected for ELISA-based ACBP detection.

(N) Longitudinal evolution of human ACBP levels in mouse plasma after tumor implantation by ELISA. Data from representative mice are shown in a representative line chart (n = 6).

(O) HUH-7 cell-derived human ACBP in mouse plasma correlates with tumor size (n = 18). Note that tumors were monitored by sonography at different time points post inoculation to create heterogeneity in tumor size.

p values were calculated by Welch's t test (A), Kruskal-Wallis test with Dunn's post hoc test (B, C, and G), Welch's one-way ANOVA with Games-Howell test (D), log rank test (E), Mann-Whitney U test (F, H, and I), and Spearman's rank correlation (J, K, L, and O).

CellPress
OPEN ACCESS

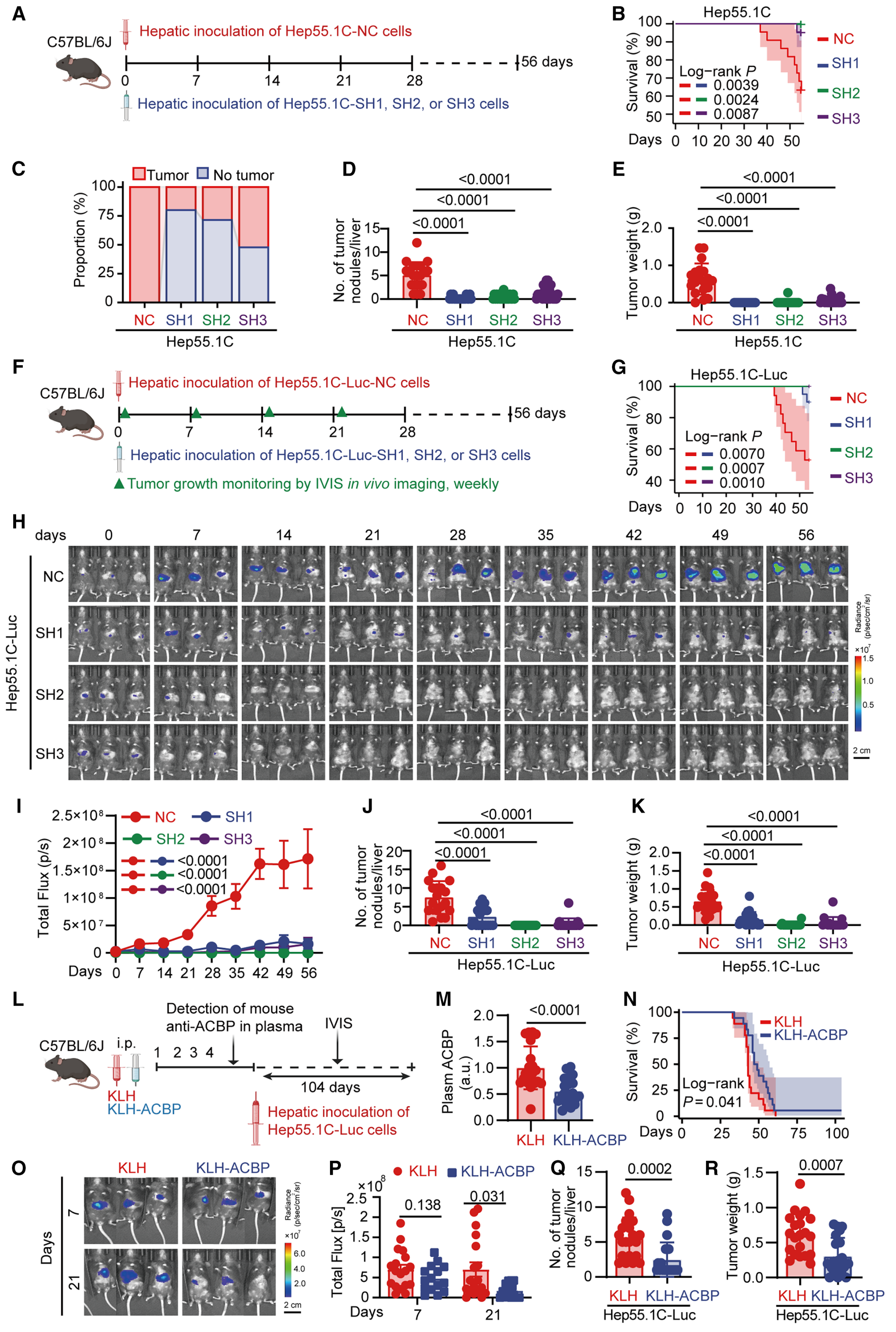

(legend on next page)

ACBP/Dbi mRNA and protein expression as well as circulating ACBP/Dbi concentrations, and inhibited the development of HCC. Thus, all protocols of ACBP/DBI inhibition reduced the size and number of WD/CCl_4-induced tumor lesions developing in the liver (Figures 4E, 4F, 4K, 4L, 4Q, and 4R). Similar anti-MASH, anti-fibrotic, and oncosuppressive effects were observed for KLH-ACBP/Dbi vaccination (as compared to KLH-only-vaccinated controls) when the disease was induced by a combination of high-fat diet (HFD) and the carcinogen diethylnitrosamine (DEN) (Figures 4S–4X and S7S–S7X). In addition, the hepatocyte-specific knockout of ACBP/DBI was sufficient to suppress liver carcinogenesis in mice receiving a carcinogenic WD plus CCl_4 (Figures S8A–S8F).

Altogether, these results indicate that hepatic carcinogenesis is favored by ACBP/DBI. This effect of ACBP/DBI is at least partially mediated by the extracellular pool of ACBP/DBI acting on GABAA receptors.

ACBP/DBI neutralization improves immunosurveillance

To elucidate the mechanisms through which ACBP/DBI facilitates hepatic carcinogenesis, we performed liver bulk RNA sequencing (RNA-seq) on three of the aforementioned models, namely (1) WD + CCl_4-treated *Dbi*$^{+/+}$ versus *Dbi*$^{-/-}$ mice, (2) WD + CCl_4-treated KLH-only versus KLH-ACBP-vaccinated mice, and (3) HFD + DEN-treated KLH-only versus KLH-ACBP-vaccinated animals. We then compared the effects of unrestrained ACBP/DBI action to 25 publicly available datasets detailing the deregulation of molecular pathways in different human liver diseases/injury (Figure 5A). Non-supervised hierarchical clustering indicated that many molecular pathways that are downregulated by ACBP/DBI inhibition are upregulated in several liver diseases (or conversely molecular pathways upregulated by ACBP/DBI inhibition are downregulated in such liver diseases), irrespective of their etiology (alcoholic, inflammatory, metabolic, or toxic), with the notable exception of infection by hepatitis viruses B, C, and D, which do not cluster with ACBP/DBI inhibition (Figure 5A).

In the three RNA-seq datasets that reflect ACBP/DBI inhibition, several gene ontology terms suggesting immunosuppression were downregulated, as this applies to a signature specific for regulatory T cells in lymph nodes,[26] pro-inflammatory T helper (Th)17 cells,[27] and polarized Th2 cells[28] (Figure 5B). According to this prediction, orthotopic Hep55.1C HCC tumors were highly responsive to combination treatment with a neutralizing monoclonal antibody (mAb) against ACBP/DBI plus a PD-1-blocking antibody. This combination was significantly more efficient in reducing tumor growth than each of the two monotherapies (anti-ACBP/DBI alone or PD-1 blockade alone), thus markedly extending survival (Figures 5C–5F). T cells infiltrating orthotopic Hep55.1C liver cancers were characterized by immunofluorescence cytometry (Figures S9A–S9C). Anti-ACBP/DBI increased the infiltration of such tumors by Th cells ($CD3^+CD4^+Foxp3^-$) and improved the ratio of Th cells over regulatory T cells (Tregs, $CD3^+CD4^+Foxp3^+$), as well as that of cytotoxic T cells (CTLs, $CD3^+CD8^+$) over Tregs. In addition, anti-ACBP/DBI enhanced the expression of ICOS1 on Th cells and CTLs, as it reduced the expression of PD1 on Tregs. Moreover, as compared to anti-PD-1-treated tumors, the combination of anti-PD-1 plus anti-ACBP/DBI reduced the expression of TIGIT on tumor-infiltrating Th cells (Figure 5G). These changes in the composition of the immune infiltrate indicate an immunostimulatory effect of ACBP/DBI blockade on T lymphocytes present in the tumor microenvironment. We conclude that ACBP/DBI inhibition can enhance the susceptibility of HCC to immunotherapy.

ACBP/DBI neutralization reduces HCC proliferation

Cell cycle-associated genes were strongly downregulated in all three RNA-seq datasets reflecting ACBP/DBI inhibition (Figure 5B). A bioinformatic analysis identified commonly up- or downregulated genes in livers from (1) WD + CCl_4-treated *Dbi*$^{+/+}$ versus *Dbi*$^{-/-}$, (2) WD + CCl_4-treated, or (3) HFD + DEN-treated KLH-only versus KLH-ACBP/Dbi-vaccinated mice (Figures S10A and S10B). We then identified genes downregulated by ACBP/DBI inhibition that are also overexpressed in human HCC and are associated with poor prognosis of HCC (Figures S10C and S10D). Among these ACBP/DBI-dependent, disease-relevant genes, many were associated with cell cycle advancement (and in particular mitosis) (Figure S10E). Quantitative reverse-transcription PCR (RT-qPCR) measurements confirmed that ACBP/BDI inhibition downregulated genes required for cell cycle advancement (such as *Ccnd1*, *Ccne1*, *Cdk4*, *Cdk6*, or *Pcna*) and upregulated genes that block the cell cycle (such as *Atr*, *Gadd45a*, *Gadd45b*, *Cdkn1a*, or *Cdkn2a*) (Figure S11A). Similarly, knockdown of *DBI* in HEP-G2 cell lines

Figure 2. Intra- and extracellular ACBP/DBI inhibition blunt tumor development in an orthotopic transplantation model

(A) Schematic diagram of the orthotopic HCC model involving intrahepatic injection of Hep55.1C cell-derived *DBI* depletion clones (SH1, SH2, and SH3) or parental control clones (NC).

(B–E) Log rank test for survival curve, tumor incidence, number of HCC nodules, and tumor weight of mice at endpoint (n = 19–23/group).

(F) Schematic diagram of the orthotopic HCC model using Hep55.1C-Luc-derived cell clones (NC, SH1, SH2, and SH3) (n = 18–19/group).

(G) Survival of mice treated as showed in (F).

(H) Representative IVIS images of HCC lesions for the different experimental conditions (n = 3/group).

(I) Quantification of total intensity of tumor lesions from IVIS images (n = 18–19/group).

(J and K) Number of HCC nodules and tumor weight of mice at endpoint (n = 18–19/group).

(L) Schematic diagram of the orthotopic HCC mouse model (Hep55.1C-Luc) treated with KLH/KLH-ACBP.

(M) Plasma ACBP levels.

(N) Survival of mice treated as shown in (L).

(O and P) Representative IVIS images and quantification of tumor burden by IVIS imaging (n = 15–21/group).

(Q and R) Number of HCC nodules and tumor weight of mice at endpoint (n = 15–21/group). Scale bars: 2 cm (H, N).

p values were calculated by log rank test (B, G, and N), Kruskal-Wallis test with Dunn's post hoc test (D, E, J, and K), two-way ANOVA (I), and Mann-Whitney U test (M, P, Q, and R). The mean values ± SEM are shown.

CellPress
OPEN ACCESS

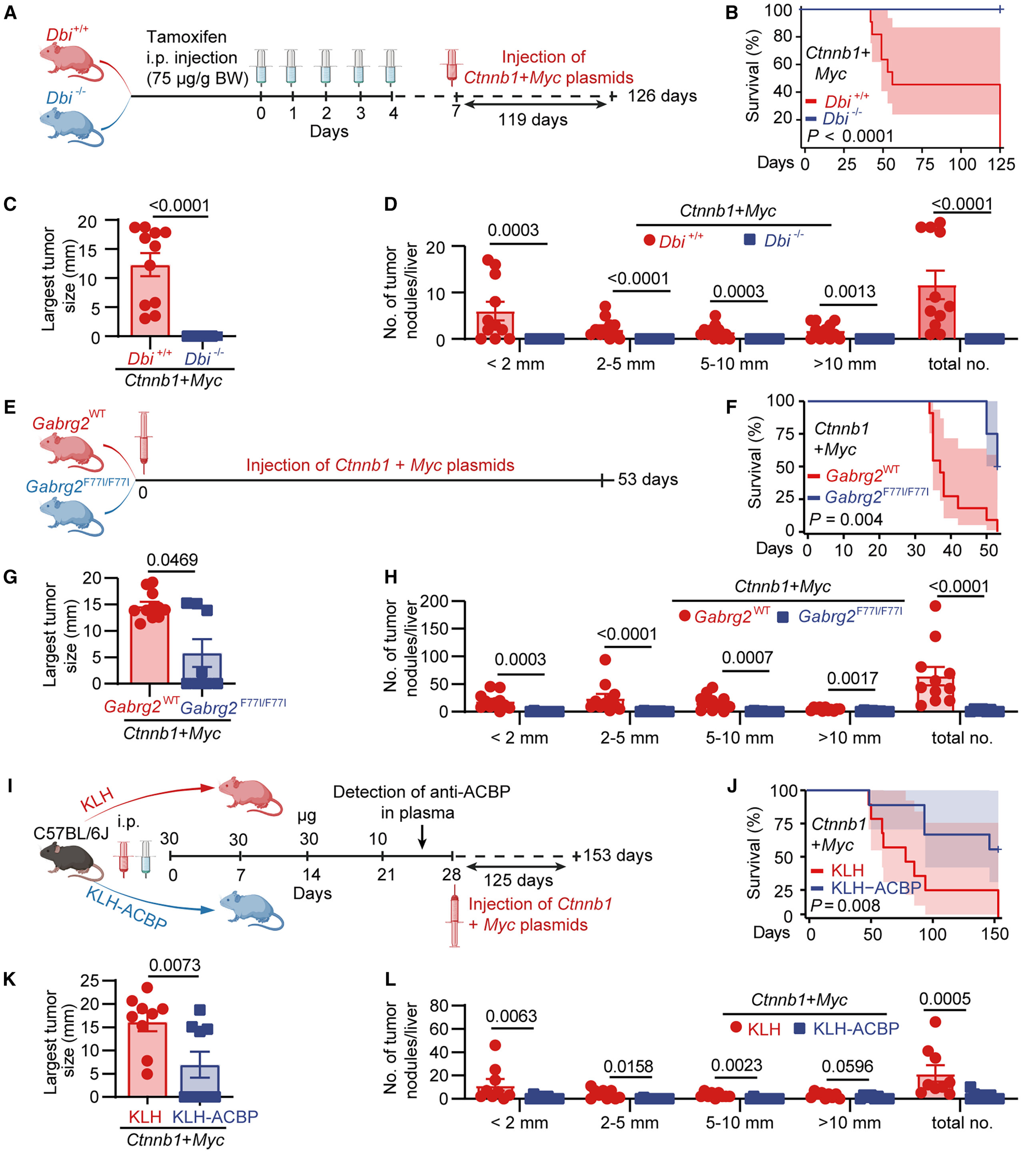

Figure 3. Intra- and extracellular ACBP/DBI inhibition delay hepatic oncogenesis driven by *Myc* plus *Ctnnb1*

(A) Schematic diagram of *Myc*/*Ctnnb1*-induced hepatic carcinogenesis in tamoxifen-induced conditional *Dbi* knockout mice ($Dbi^{-/-}$) and control mice ($Dbi^{+/+}$).

(E) Schematic diagram of *Myc*/*Ctnnb1*-induced hepatocarcinogenesis in Gabrg2-mutated ($Gabrg2^{F77I/F77I}$) and control ($Gabrg2^{WT}$) mice.

(I) Schematic diagram of *Myc*/*Ctnnb1*-induced hepatic tumorigenesis in KLH/KLH-ACBP-immunized mice.

(B, F, and J) Survival of mice in days (n = 8–12/group).

(C, G, and K) Size of the largest tumor per mouse (n = 8–12/group).

(D, H, and L) Quantification of the number of tumors with different size (<2, 2–5, 5–10, and >10 mm) (n = 8–12/group). Error bars represent mean ± SEM. p values were calculated by log rank test (B, F, and J) and Mann-Whitney U test (C, D, G, H, K, and L).

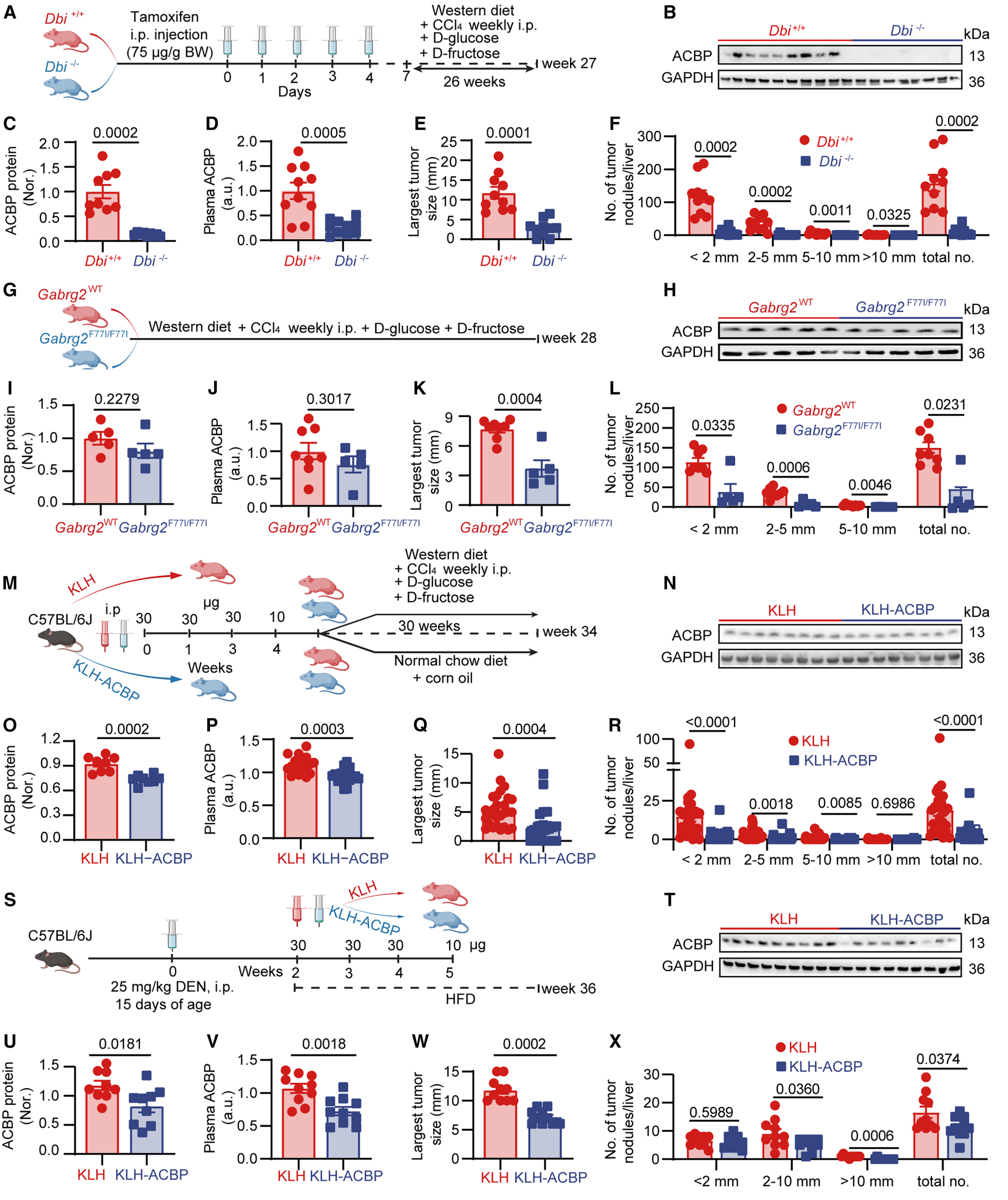

Figure 4. Intra- and extracellular ACBP/DBI inhibition impaired MASH-driven hepatocarcinogenesis

(A) Schematic diagram of hepatic carcinogenesis induced by Western-style diet (WD) plus CCl_4 in tamoxifen-induced conditional *Dbi* knockout mice (*Dbi* $^{-/-}$) and control mice (*Dbi* $^{+/+}$).

(G) Schematic diagram of WD plus CCl_4-induced hepatic carcinogenesis in Gabrg2-mutated (*Gabrg2*$^{F77I/F77I}$) and control (*Gabrg2*^{WT}) mice.

(M) Schematic diagram of WD plus CCl_4-induced hepatic carcinogenesis in KLH/KLH-ACBP-immunized mice.

(legend continued on next page)

elicited transcriptional signs of cell cycle blockade (Figure S11B), in accord with the reduced oncogenic and proliferative ability of such *DBI*-depleted cells (Figures S4E–S4J).

Prompted by these results, we assessed cell proliferation in livers from mice subjected to carcinogen-induced oncogenesis using immunohistochemical detection of Ki67 (Figures S12A–S12E) and PCNA (Figure S13). Of note, ACBP/DBI inhibition by *Dbi* knockout, *Gabrg2* mutation, or KLH-ACBP/DBI vaccination resulted in reduced proliferation, both in tumor lesions (if detectable) and in the non-malignant hepatic parenchyma, irrespective of the precise oncogenic stimulus (CCl_4 or DEN) (Figures S12A–S12E and S13). Intraperitoneal injection of a neutralizing anti-ACBP/DBI mAb inhibited hepatocyte proliferation both after sham operation and after partial hepatectomy, which induces liver regeneration[29] (Figure S12E), suggesting a general pro-proliferative effect of ACBP/DBI in normal hepatocytes.

Finally, we isolated HCC cells from $Dbi^{+/+}$ versus $Dbi^{-/-}$ mice subjected to WD + CCl_4-triggered carcinogenesis (Figure S12F). $Dbi^{-/-}$ HCC cells exhibited comparatively low expression of proliferation markers (Ki67 and PCNA) and malignancy-linked markers (AFP, CK19, and GPC3) (Figure S12G), attenuated clonogenic potential (Figure S12H), and reduced *in vitro* proliferation (Figure S12I). These findings indicate that ACBP/DBI is required for full-blown HCC proliferation. We conclude that ACBP/DBI has profuse pro-proliferative effects on normal and transformed hepatocytes.

ACBP/DBI neutralization sensitizes to the induction of ferroptosis

The aforementioned bioinformatic analysis identified alterations in pathways of cellular self-consumption (Figure 5B). Thus, ACBP/DBI suppression entailed a reduction in transcripts relevant to apoptosis and necroptosis but an increase in the expression of genes relevant to ferroptosis (Figure 5B). In addition, ACBP/DBI inhibition enhanced the expression of autophagy-related genes (Figure 5B), commensurate with the well-documented autophagy-repressive function of ACBP/DBI,[30,31] as well as the known pro-ferroptotic effects of autophagy.[32,33]

Validation by RT-qPCR confirmed the upregulation of ferroptosis driver genes as well as the downregulation of ferroptosis suppressor genes in livers from (1) WD + CCl_4-treated $Dbi^{-/-}$ versus $Dbi^{+/+}$ mice, (2) WD + CCl_4-treated $Gabrg2^{F77I/F77I}$ versus $Gabrg2^{WT}$ mice, (3)WD + CCl_4-treated mice, and (4) WD + DEN-treated KLH-ACBP/Dbi versus KLH-only-vaccinated mice (Figure 6A). Moreover, immunoblots confirmed the increased protein expression of ferroptosis effectors (such as ACSL4, KEAP1, NCOA4, and POR) but decreased expression of ferroptosis suppressors (such as ACSL3, BMAL1, GPX4, and SQSTM1/p62) after inhibition of ACBP/DBI (Figures 6B and S14A). Such shifts from ferroptosis suppression toward execution were also observed for HCC clones derived from the livers of WD + CCl_4-treated $Dbi^{-/-}$ versus $Dbi^{+/+}$ mice, both at the mRNA (Figure S14B) and at the protein levels (Figures S14C–S14E). Spatial transcriptomics or spatially resolved mass spectrometric metabolomics coupled to hematoxylin-eosin staining allowed to distinguish non-malignant and malignant areas of the liver (Figures 6C and S15A–S15C). Of note, inhibition of ACBP/DBI by *Dbi* knockout or KLH-ACBP/Dbi vaccination caused a higher expression of ferroptosis drivers in apparently (still) non-malignant tissue compared to ACBP/DBI-uninhibited controls (non-tumor and tumor tissues). In ACBP/DBI-uninhibited controls, ferroptosis suppressors were upregulated in tumors as compared to the non-malignant adjacent liver tissue (Figures 6C and 6D). Hence, the alterations in ferroptosis-relevant gene expression levels found in total livers were detected both in HCC and in non-malignant tissues. In non-tumor tissues, spatial transcriptomics also revealed a downregulation of immunosuppressive genes and an upregulation of immunostimulatory genes upon ACBP/DBI inhibition (Figure 6E), supporting improved immunosurveillance.

In accord with the gene/protein expression data, HCC cells were particularly vulnerable to losing their viability in response to the combination of *Dbi* knockout and the addition of pharmacological ferroptosis inducers including RSL3, imidazole ketone erastin (IKE), linoleic acid, and linolenic acid (Figures 7A–7D). Moreover, orthotopic Hep55.1C cancers responded to treatment with a combination of anti-ACBP mAb plus ferroptosis induction (with RSL3 or IKE) more efficiently than to standalone therapies with either anti-ACBP mAb or ferroptosis inducers (Figures 7E–7P and S15D). This combination was more efficient in reducing tumor burden than each of the two monotherapies (anti-ACBP alone or RSL3/IKE alone), thus markedly extending survival (Figures 7E–7P and S15E).

We conclude that inhibition of DBI sensitizes HCC to therapeutic interventions with ferroptosis inducers.

DISCUSSION

ACBP/DBI has previously been shown to contribute to the pathogenesis of a wide array of non-malignant experimental liver diseases ranging from acute hepatic necrosis to steatohepatitis and fibrosis/cirrhosis.[8,13,14] Here, we demonstrate that ACBP/DBI also contributes to the development and progression of HCC induced by a variety of rather distinct methods including (1) intrahepatic inoculation of HCC cells, (2) oncogene-induced transformation, and (3) chronic challenge with the hepatotoxin CCl_4 or (4) the mutagen DEN in the context of WD. We used a compendium of genetic methods to inhibit the ACBP/DBI system (i.e., knockdown or knockout of *DBI* and mutation of its receptor GABRG2), as well as immunological approaches

(S) Schematic diagram of HFD plus DEN-induced hepatic carcinogenesis in KLH/KLH-ACBP-immunized mice.
(B, H, N, and T) Representative immunoblots detecting ACBP protein in the liver (n = 5–9/group). GAPDH was used as a loading control.
(C, I, O, and U) Quantification of western blots (n = 5–9/group). Densitometric ratios of ACBP/GAPDH were normalized to control groups, shown as (Nor.).
(D, J, P, and V) Plasma ACBP levels (n = 5–18/group). Values were normalized to control groups, presented as (a.u.).
(E, K, Q, and W) Quantification of the largest tumor size of each treatment group (n = 5–27/group).
(F, L, R, and X) Quantification of the number of tumors with different size (n = 5–27/group). Error bars represent mean ± SEM. p values were calculated by Welch's t test (C, D, and E), Mann-Whitney U test (F, Q, R, and W), t test (I, J, K, O, P, U, and V), as well as t test and Mann-Whitney U test (L and X).

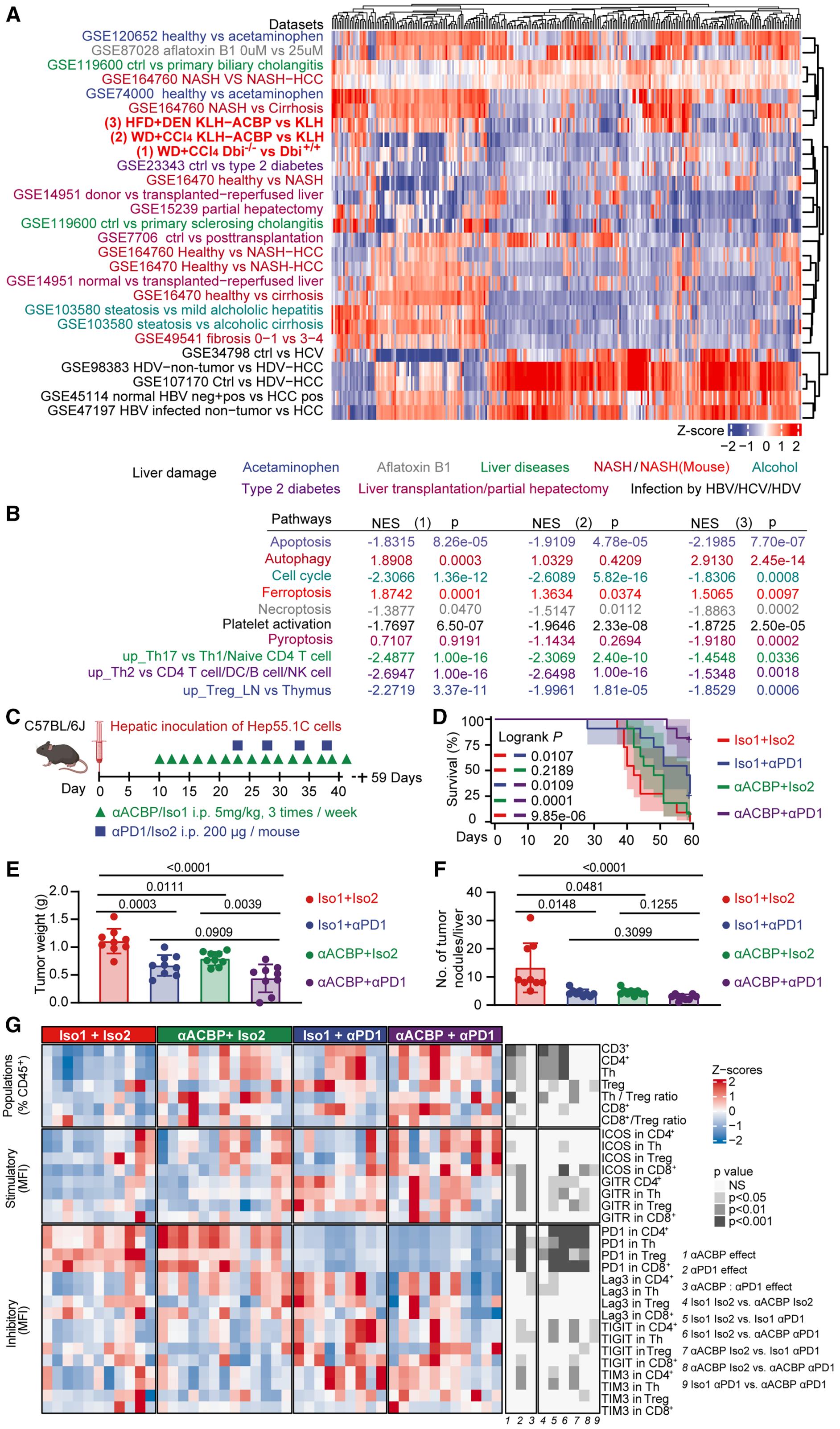

(legend on next page)

(i.e., neutralizing antibodies against ACBP/DBI), finding that all these strategies mitigated hepatic carcinogenesis. This preclinical evidence in favor of pro-HCC effects of ACBP/DBI is backed up by clinical correlations showing that aggressive HCC is associated with the upregulation of *DBI* mRNA, as well as with an increase in circulating ACBP/DBI protein concentrations.

Based on the literature, ACBP/DBI can mediate cell-autonomous cancer cell-supportive metabolic effects, as indicated by the fact that glioblastoma growth was enhanced by transfection with the wild-type ACBP/DBI protein but not by a mutant (K32A) that lacks acyl-CoA binding capacity and fails to stimulate fatty acid oxidation,[18] but still is able to bind to GABRG2 and to mediate metabolic effects when injected into mice.[21] Here, we used several methods to inhibit intracellular ACBP/DBI, namely, its knockdown in HCC cell lines and its tamoxifen-inducible knockout in mice, as well as methods to interfere with extracellular ACBP/DBI, namely, its antibody-mediated neutralization and the mutation of its receptor GABRG2. Both the inhibition of intracellular and extracellular ACBP/DBI reduced hepatic carcinogenesis, suggesting that the two pools of ACBP/DBI are disease relevant. As a caveat, however, it can be argued that a significant amount of extracellular ACBP/DBI is produced by malignant cells (as suggested by the fact that human HCCs present in mice generate ACBP/DBI that becomes detectable in the circulation) and then could affect the tumor microenvironment and even systemic circuitries. Conversely, as shown here, inhibition of extracellular ACBP/DBI (by antibodies or GABRG2 mutation) reduced the expression of liver ACBP/DBI, in accord with the interruption of a feedback loop through which extracellular ACBP/DBI acting on GABRG2 activates the transcription factor peroxisome proliferator-activated receptor-g to stimulate the transcription of the *DBI* gene.[13] Hence, the intra- and extracellular pools of ACBP/DBI are functionally linked, rendering it difficult to weigh their relative contribution to hepatic oncogenesis. That said, it appeared that the knockout of ACBP/DBI had more consistent oncosuppressive effects than F77I GABRG mutation and vaccination with KLH-ACBP/DBI against hepatocarcinogenesis induced by oncogenes (*Myc* plus *Ctnnb1*) or hepatic damage (by WD plus CCl_4), arguing in favor of the importance of both pools of ACBP/DBI.

Tumorigenesis cannot be only ascribed to cell-intrinsic alterations (such as (epi)genetic mutations generated in the context of cellular stress and excessive proliferation)[34] but also implies cell-extrinsic alterations including excessive inflammation[35] and the failure of immunosurveillance, i.e., the elimination of (pre-) malignant cells by innate or acquired immune effectors.[36,37] It appears probable that ACBP/DBI neutralization affects both cell-intrinsic and -extrinsic facets of liver cancer, in line with the fact that both hepatocytes and immune cells express GABRG2. Thus, beyond its cell biological effects, inhibition of ACBP/DBI consistently reduced signs of liver inflammation leading to excessive macrophage infiltration and fibrosis but also reduced the expression of genes associated with immunosuppressive T cell subtypes (such a T_{regs}, T_H2, and T_H17). In addition, ACBP/DBI neutralization enhanced the expression of genes favoring immune responses in non-tumoral liver tissue, as determined by spatial transcriptomics, and improved the ratio among immunostimulatory and immunosuppressive HCC-infiltrating T lymphocytes (i.e., the Th:Treg and CTL:Treg ratios), as determined by immunofluorescence cytometry. Accordingly, inhibition of extracellular ACBP/DBI by means of a mAb sensitized HCC to immunotherapy by PD-1 blockade. In this context, it may be important to note that ACBP/DBI neutralization did not modify the hepatic expression of genes directly involved in the response to PD-1 blockade (such as PD-1 itself and that of its ligands PD-L1/CD274 and PD-L2/PDCD1LG2). Thus, the immunotherapy-enhancing effect of ACBP/DBI blockade is likely mediated by a general improvement of the immune tonus.

At the cancer cell-specific level, we observed that ACBP/DBI inhibition induced several phenomena beyond the well-studied enhancement of autophagy. Thus, any strategy for inhibiting intracellular or extracellular ACBP/DBI resulted in reduced proliferation, not only of malignant HCC cells but also of normal hepatocytes, even in mice that were subjected to partial hepatectomy (to study liver regeneration) and in control animals. This may reflect the known trophic and anabolic effects of ACBP/DBI, which stimulates the AKT/mTORC1 pathway[8] involved in liver growth stimulation.[38,39] In addition, we observed that ACBP/DBI neutralization reduced the expression of pro-apoptotic and pro-necrotic genes. However, regardless of the mode of inhibition (knockout, receptor mutation, or antibody-mediated neutralization), ACBP/DBI inhibition resulted in the upregulation of 13 pro-ferroptotic mRNAs and the downregulation of even more anti-ferroptotic mRNAs. This effect, which could be validated at the protein level, was accompanied by increased susceptibility of HCC cells to pharmacological induction of ferroptosis both *in vitro* and *in vivo*. It is well established that autophagy (which is induced by ACBP/DBI inhibition) can contribute to ferroptotic cell death,[32,33] but autophagy generally inhibits apoptosis and often interferes with necroptosis.[32,40] Further cell biological studies must explore the molecular mechanics

Figure 5. ACBP/DBI neutralization enhances the susceptibility of HCC to immunotherapy

(A) Cross-species comparison of dysregulated molecular pathways between three mouse models and human liver diseases. Normalized enrichment score (NES) of each Kyoto Encyclopedia of Genes and Genomes (KEGG) pathway is row-normalized and presented as *Z* score. Euclidean distance was calculated for row and column clustering analysis.

(B) Representative KEGG pathways in three mouse datasets generated in this study. NESs are shown.

(C) Schematic diagram of anti-ACBP plus anti-PD-1 combination therapy tested in orthotopic Hep55.1C HCC model.

(D) Survival of the mice in days (n = 9/group).

(E and F) Tumor weight and number of tumor nodules per mouse in each treatment group (n = 9/group). Error bars represent means ± SEM.

(G) Analysis of the T lymphocyte infiltration in the orthotopic Hep55.1C HCC model treated with anti-ACBP plus anti-PD1 (see experimental design in Figure S9A) by immunofluorescence cytometry (n = 9–13/group). Results are shown as *Z* scores for each mouse.

p values are calculated by t test (B), log rank test (D), one-way ANOVA post hoc Tukey HSD test (E), Kruskal-Wallis test with Dunn's multiple comparison (F), and two-way ANOVA test (G). p values in (G) correspond to the global effects (1–3) and post hoc pairwise comparisons (4–9), respectively.

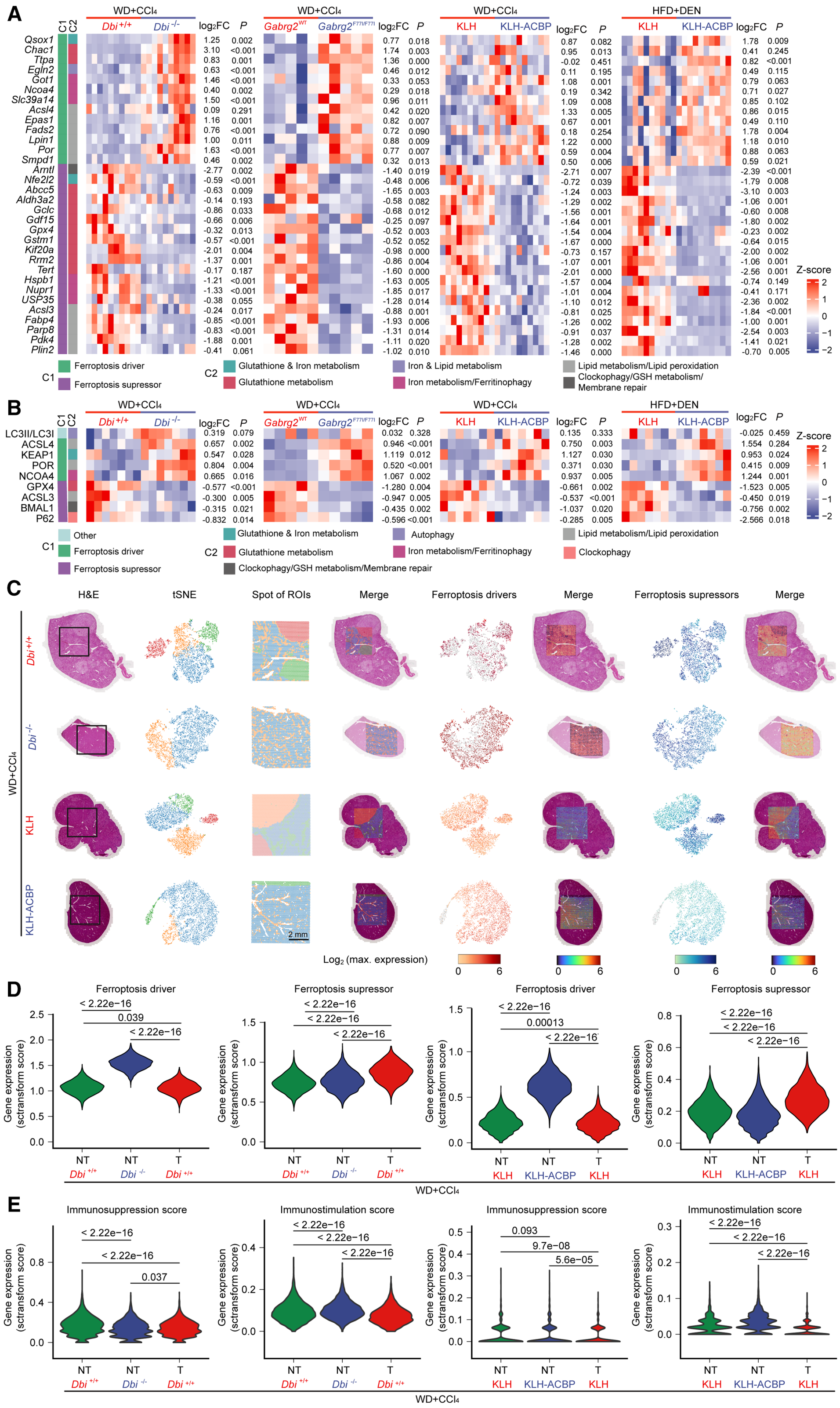

(legend on next page)

underlying the crosstalk between these pathways of self-consumption in HCC.

In conclusion, it appears that hepatocellular carcinogenesis involves the contribution of ACBP/DBI, meaning that the neutralization of its intracellular and extracellular pools reduces or delays the manifestation of HCC in various preclinical models. In addition, we observed that inhibition of ACBP/DBI inhibits proliferation and sensitizes established HCC to PD-1-targeted immunotherapy, as well as pharmacological induction of ferroptosis. In patients with HCC, both intralesional and circulating ACBP/DBI are associated with more aggressive tumors and poor prognosis, arguing in favor of the possible clinical translation of these findings.

Limitations of the study

Limitations also apply to our study, which has not explored the detailed mechanisms linking ACBP/DBI/$GABA_AR$ interactions to intracellular signaling cascades that explain the broad impact of ACBP/DBI neutralization on HCC. Similarly, we have not determined whether the cell-autonomous effects of ACBP/DBI favoring HCC proliferation are mediated through $GABA_AR$ or other mechanisms that might involve the lipid metabolism-enhancing effects of ACBP/DBI. Other limitations concern the absence of data on ACBP/DBI protein in HCC samples, meaning that we only confirmed the negative prognostic impact of *DBI* mRNA in HCC samples (and ACBP/DBI protein in plasma), as well as the absence of food intake measurements for each individual mouse included in this study. We used orthogonal approaches to inhibit the ACBP/DBI system (by knockdown, knockout, antibody-mediated neutralization, or receptor mutation) and to induce HCC (orthotopic inoculation of HCC cells, hydrodynamic injection of vectors expressing MYC and beta-catenin, WD plus CCl_4, and WD plus DEN) that yielded similar conclusions with respect to the pro-HCC effects of ACBP/DBI. However, it is well possible that these pro-HCC effects are heterogeneous or multipronged. The likely complex links between ferroptosis and oncogenesis[41,42] or between ferroptosis and immunomodulation[43] have not been disentangled in this study. At this point, we cannot establish a clear hierarchical relationship among the autophagy-inducing, cell cycle-inhibitory, ferroptosis-sensitizing, and immunostimulatory effects of ACBP/DBI neutralization, although the width of these effects suggests pleiotropic actions of ACBP/DBI. We found that ACBP/DBI inhibition could be favorably combined with ferroptosis-inducing agents of PD-1 blockade. However, we did not investigate the triple combination of (1) ACBP/DBI inhibition, (2) ferroptosis induction, and (3) PD-1 blockade. These questions must be addressed in future studies.

RESOURCE AVAILABILITY

Lead contact

Requests for further information and resources should be directed to and will be fulfilled by the lead contact, Guido Kroemer (kroemer@orange.fr).

Materials availability

This study did not generate new unique reagents.

Data and code availability

- The sequencing data reported in this paper have been deposited in NCBI GEO database with accession number GEO: GSE255799 and GSE296545 (URL: https://www.ncbi.nlm.nih.gov/geo/) and are publicly available as of the date of publication. The metabolomics data reported in this paper have been deposited in Metabolomics Workbench with accession number ST003957 (URL: https://www.metabolomicsworkbench.org/). The accession numbers are listed in the key resources table as well.
- This study did not report new original code.
- Any additional information required to reanalyze the data reported in this paper is available from the lead contact upon request.

ACKNOWLEDGMENTS

The authors thank the CRC core facilities. We thank the help by Olivia Bawa from the PETRA platform of Gustave Roussy Institute for technical support. G.K. is supported by the Ligue Contre le Cancer (équipe labellisée); Agence National de la Recherche (ANR-22-CE14-0066 VIVORUSH, ANR-23-CE44-0030 COPPERMAC, ANR-23-R4HC-0006 Ener-LIGHT); Association pour la recherche sur le cancer (ARC); Cancéropôle Île-de-France; Fondation pour la Recherche Médicale (FRM); European Research Council Advanced Investigator Award (ERC-2021-ADG, grant no. 101052444; project acronym: ICD-Cancer, project title: Immunogenic cell death [ICD] in the cancer-immune dialogue); the ERA4 Health Cardinoff Grant Ener-LIGHT; European Union Horizon 2020 research and innovation programs Oncobiome (grant agreement no. 825410, project acronym: ONCOBIOME, project title: Gut OncoMicrobiome Signatures [GOMS] associated with cancer incidence, prognosis, and prediction of treatment response), Prevalung (grant agreement no. 101095604, project acronym: PREVALUNG EU, project title: Biomarkers affecting the transition from cardiovascular disease to lung cancer: toward stratified interception), and Neutrocure (grant agreement no. 861878, project acronym: Neutrocure; project title: Development of "smart" amplifiers of reactive oxygen species specific to aberrant polymorphonuclear neutrophils for treatment of inflammatory and autoimmune diseases, cancer, and myeloablation); national support managed by the Agence Nationale de la Recherche under the France 2030 programme (reference number 21-ESRE-0028, ESR/Equipex+ Onco-Pheno-Screen); Hevolution Network on Senescence in Aging (reference HF-E Einstein Network); Institut National Du Cancer (INCa); Institut Universitaire de France; PAIR-Obésité INCa_18713, the RHUs Immunolife and LUCA-pi (ANR-21-RHUS-0017 and ANR-23-RHUS-0010, both dedicated to France Relance 2030); Seerave Foundation; and SIRIC Cancer Research and Personalized Medicine (CARPEM, SIRIC CARPEM INCa-DGOS-Inserm-ITMO Cancer_18006 supported by Institut National du Cancer, Ministère des Solidarités et de la Santé, and INSERM). This study contributes to the IdEx Université de Paris Cité ANR-18-IDEX-0001. G.K., J.Z.-R., and C.D. are supported by INCa PAIR Obesity & Cancer. J.Z.-R. is supported by the Ligue Contre

Figure 6. ACBP/DBI inhibition enhances ferroptosis sensitivity at the transcriptional level

(A) RT-qPCR analysis showed upregulation of ferroptosis-promoting genes and downregulation of ferroptosis-inhibiting genes by ACBP/DBI inhibition. Results were displayed in heatmaps (n = 5–10/group).

(B) Quantification of western blots shown in Figure S12A. Ferroptosis and autophagy-related proteins were detected by immunoblotting. Results were quantified and displayed in heatmaps (n = 5–7/group).

(C) Spatial transcriptomic landscape of individual liver sections from WD/CCl_4-induced HCC in $Dbi^{+/+}$/$Dbi^{-/-}$ mice or KLH/KLH-ACBP-immunized mice. Black rectangles (6.5 mm $\times$ 6.5 mm) indicate regions of interest for each sample. Scale bars in spot images indicate 2 mm. Error bars represent means ± SEM. p values were calculated by t test, Welch's t test, or Mann-Whitney U test (A, B).

(D) Quantification of genes classified as ferroptosis drivers or suppressors in non-tumor (NT) and tumor (T) areas defined by H&E staining in (C).

(E) Immunosuppression scores and immunostimulation scores of non-tumor (NT) and tumor (T) areas.

p values were calculated by means of the Wilcoxon signed-rank test (D and E).

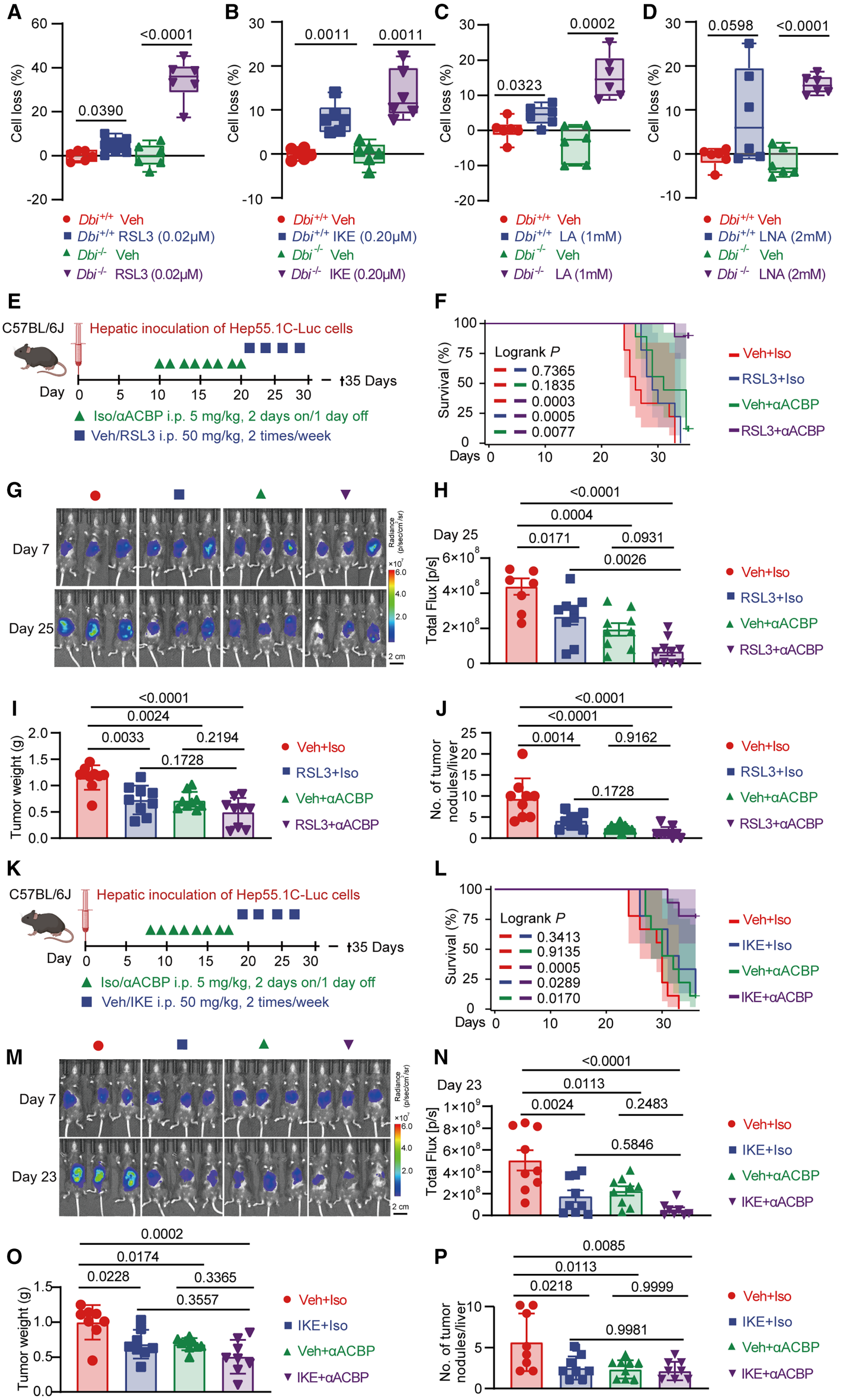

(legend on next page)

le Cancer, SIRIC Cancer Research and Personalized Medicine (CARPEM), and THRIVE EU Horizon funding Mission Cancer (EU-HORIZON-MISS-2023-CANCER-01). This study contributes to the IdEx Université de Paris ANR-18-IDEX-0001. O.K. is supported by INCa and ARC. Views and opinions expressed are those of the author(s) only and do not necessarily reflect those of the European Union, the European Research Council, or any other granting authority. Neither the European Union nor any other granting authority can be held responsible for them. S. Li, H.C., L. Pan, and H.P. are supported by the China Scholarship Council (CSC, file no. 201907060011, file no. 201908070134, file no. 202006320074, and file no. 201908500100, respectively). U.N.R. was supported by Axudas de apoio á etapa de formación posdoutoral da Xunta de Galicia-GAIN. M.P. and F.R. were supported by European Union NextGenerationEU through the Italian Ministry of University and Research under PNRR-MAC2-II.3 project PE6 "Heal Italia." F.R. was supported by Associazione Italiana Ricerca sul Cancro (AIRC 27116). N°Expediente: IN606B-2021/015. E.G.S.D. was supported by the University of Las Palmas de Gran Canaria (ULPGC), financed by the Ministry of Universities, granted by Order UNI/501/2021 of May 26, and by the European Union-Next Generation EU Funds. J.-C.N. was supported by Association Française pour l'Étude du Foie (AFEF) 2022 projet radio-moléculaire, Agence Nationale De La Recherche (ANR) 2022 SYSTHEC, Agence nationale de recherches sur le sida et les hépatites virales (ANRS) 2023 CSS13 HBV-LIRAGE ECTZ232901, and SIRIC CAncer Research in multiple dimensions to accelerate PrEcision Medicine (CARPEM) INCa-DGOS-Inserm-12561. Figures 1M, 2A, 2F, 2L, 3A, 3E, 3I, 4A, 4G, 4M, 4S, 5C, 6F, 7E, 7K, S6D, S8A, S9A, and S12F and the graphical abstract were created with "BioRender.com." The funders had no role in the design of the study, in the writing of the manuscript, or in the decision to publish the results.

AUTHOR CONTRIBUTIONS

S. Li, O.M., F.L., I.M., M.C.M., and G.K. designed research. G.K. and M.C.M. supervised the study. S. Li performed most of the *in vitro* and *in vivo* experiments. O.M., F.L., J.P., H.C., F.R., C.C., G.A., U.N.R., E.G.S.D., O.K., P.L., L. Zhao, H.P., L.M., E.X., M.L.G., and S. Lachkar helped with the research. S.D., A.C., and F.A. performed the metabolomic experiments. S. Li, O.M., and F.L. contributed to setting up novel *in vivo* models. S. Li, S.D., C.C., L. Pan, L. Poupel, and V.C. analyzed the data. L. Poupel and C.K. performed the spatial transcriptomic experiments. S. Li prepared the figures. G.K. and S. Li wrote the paper. G.K., M.C.M., J.Z.-R., J.-C.N., M.P., L. Zitvogel, I.M., S.C., C.D., and L.S. provided intellectual input and edited the paper.

DECLARATION OF INTERESTS

G.K. and O.K. have been holding research contracts with Daiichi Sankyo, Kaleido, Lytix Pharma, PharmaMar, Osasuna Therapeutics, Samsara Therapeutics, Sanofi, Sutro, Tollys, and Vascage. G.K. is on the Board of Directors of the Bristol Myers Squibb Foundation France. O.K. is a scientific co-founder of Samsara Therapeutics and holds patents covering therapeutic targeting of neurodegenerative diseases and cancer. G.K. is a scientific co-founder of EverImmune, Osasuna Therapeutics, Samsara Therapeutics, and Therafast Bio. G.K. is on the scientific advisory boards of Centenara Labs (formerly Rejuveron Life Sciences), Hevolution, and Institut Servier. G.K. is the inventor of patents covering therapeutic targeting of ACBP/DBI, aging, cancer, cystic fibrosis, and metabolic disorders. G.K.'s wife, L. Zitvogel, has held research contracts with Glaxo Smyth Kline, Incyte, Lytix, Kaleido, Innovate Pharma, Daiichi Sankyo, Pilege, Merus, Transgene, 9M, Tusk, and Roche, was on the Board of Directors of Transgene, is a cofounder of EverImmune, and holds patents covering the treatment of cancer and the therapeutic manipulation of the microbiota. G.K.'s brother, Romano Kroemer, was an employee of Sanofi and now consults for Boehringer Ingelheim. I.M. is a consultant for Osasuna Therapeutics.

STAR★METHODS

Detailed methods are provided in the online version of this paper and include the following:

- KEY RESOURCES TABLE
- EXPERIMENTAL MODEL AND STUDY PARTICIPANT DETAILS
  - HCC patient cohort
  - Animals
- METHOD DETAILS
  - Generation of stable cell lines, cell culture, and cell assays
  - Inhibition of ACBP/DBI *in vivo*
  - MASH-driven HCC model
  - Oncogene-induced HCC
  - Orthotopic transplantation of HCC cells
  - Partial hepatectomy
  - Combination therapies
  - Isolation of primary HCC cells
  - Isolation and purification of primary mouse hepatocytes and immune cells
  - Multi-omics analysis
  - Flow cytometry analyses of the immune infiltrate
  - Liver histology and Immunohistochemistry
  - Immunofluorescence
  - Enzyme-linked immunosorbent assay
  - Quantitative real-time PCR
  - Immunoblotting
- QUANTIFICATION AND STATISTICAL ANALYSIS
  - Bioinformatics analyses

SUPPLEMENTAL INFORMATION

Supplemental information can be found online at https://doi.org/10.1016/j.xcrm.2025.102232.

Received: March 13, 2024
Revised: September 20, 2024
Accepted: June 12, 2025
Published: July 7, 2025

REFERENCES

1. Sung, H., Ferlay, J., Siegel, R.L., Laversanne, M., Soerjomataram, I., Jemal, A., and Bray, F. (2021). Global Cancer Statistics 2020: GLOBOCAN Estimates of Incidence and Mortality Worldwide for 36 Cancers in 185 Countries. CA Cancer J. Clin. *71*, 209–249.

Figure 7. ACBP/DBI neutralization increased sensitivity to ferroptosis induction *in vitro* and *in vivo*

(A–D) CCK-8 viability assays of $Dbi^{+/+}$ and $Dbi^{-/-}$ HCC cells treated with ferroptosis inducers, including RSL3, IKE (imidazole ketone erastin), LA (linoleic acid), and LNA (linolenic acid) (n = 6/group). Groups receiving ferroptosis inducers were normalized to their respective $Dbi^{+/+}$ and $Dbi^{/-}$ vehicle (Veh)-treated control groups.

(E and K) Schematic diagram of anti-ACBP plus RSL3 or IKE combination therapy tested in orthotopic Hep55.1C-Luc HCC model.

(F and L) Survival of the mice in days (n = 9/group).

(G, H, M, and N) Representative IVIS images of HCC lesions and quantification of total intensity of HCC lesions from IVIS images at day 25/23, respectively (n = 7–9/group).

(I, J, O, and P) Tumor weight and number of HCC nodules of mice at the human endpoint (n = 8–9/group).

Error bars represent Min to Max (A–D). Error bars represent means ± SEM (H, I, J, N, O, and P). Scale bars: 2 cm (G and M). p values were calculated by t test (A, B, C, and D), log rank test (F, L), and one-way ANOVA (H, I, J, N, O, and P).

2. Singal, A.G., Kanwal, F., and Llovet, J.M. (2023). Global trends in hepatocellular carcinoma epidemiology: implications for screening, prevention and therapy. Nat. Rev. Clin. Oncol. *20*, 864–884.
3. Nault, J.C., Martin, Y., Caruso, S., Hirsch, T.Z., Bayard, Q., Calderaro, J., Charpy, C., Copie-Bergman, C., Ziol, M., Bioulac-Sage, P., et al. (2020). Clinical Impact of Genomic Diversity From Early to Advanced Hepatocellular Carcinoma. Hepatology *71*, 164–182.
4. Caruso, S., O'Brien, D.R., Cleary, S.P., Roberts, L.R., and Zucman-Rossi, J. (2021). Genetics of Hepatocellular Carcinoma: Approaches to Explore Molecular Diversity. Hepatology *73*, 14–26.
5. Limousin, W., Laurent-Puig, P., Ziol, M., Ganne-Carrié, N., Nahon, P., Ait-Omar, A., Seror, O., Sidali, S., Campani, C., Blanc, P., et al. (2023). Molecular-based targeted therapies in patients with hepatocellular carcinoma and hepato-cholangiocarcinoma refractory to atezolizumab/bevacizumab. J. Hepatol. *79*, 1450–1458.
6. Vogel, A., Meyer, T., Sapisochin, G., Salem, R., and Saborowski, A. (2022). Hepatocellular carcinoma. Lancet (London, England) *400*, 1345–1362.
7. Pinter, M., Scheiner, B., and Pinato, D.J. (2023). Immune checkpoint inhibitors in hepatocellular carcinoma: emerging challenges in clinical practice. Lancet Gastroenterol. Hepatol. *8*, 760–770.
8. Bravo-San Pedro, J.M., Sica, V., Martins, I., Pol, J., Loos, F., Maiuri, M.C., Durand, S., Bossut, N., Aprahamian, F., Anagnostopoulos, G., et al. (2019b). Acyl-CoA-Binding Protein Is a Lipogenic Factor that Triggers Food Intake and Obesity. Cell Metab. *30*, 1171.
9. Neess, D., Bek, S., Engelsby, H., Gallego, S.F., and Færgeman, N.J. (2015). Long-chain acyl-CoA esters in metabolism and signaling: Role of acyl-CoA binding proteins. Prog. Lipid Res. *59*, 1–25.
10. Udupa, P., Kumar, A., Parit, R., and Ghosh, D.K. (2023). Acyl-CoA binding protein regulates nutrient-dependent autophagy. Metabolism *145*, 155338.
11. Duran, J.M., Anjard, C., Stefan, C., Loomis, W.F., and Malhotra, V. (2010). Unconventional secretion of Acb1 is mediated by autophagosomes. J. Cell Biol. *188*, 527–536.
12. Dumitru, I., Neitz, A., Alfonso, J., and Monyer, H. (2017). Diazepam Binding Inhibitor Promotes Stem Cell Expansion Controlling Environment-Dependent Neurogenesis. Neuron *94*, 125–137.e5.
13. Anagnostopoulos, G., Motiño, O., Li, S., Carbonnier, V., Chen, H., Sica, V., Durand, S., Bourgin, M., Aprahamian, F., Nirmalathasan, N., et al. (2022). An obesogenic feedforward loop involving PPARγ, acyl-CoA binding protein and GABA(A) receptor. Cell Death Dis. *13*, 356.
14. Motino, O., Lambertucci, F., Anagnostopoulos, G., Li, S., Nah, J., Castoldi, F., Senovilla, L., Montegut, L., Chen, H., Durand, S., et al. (2022). ACBP/DBI protein neutralization confers autophagy-dependent organ protection through inhibition of cell loss, inflammation, and fibrosis. Proc. Natl. Acad. Sci. USA *119*, e2207344119.
15. Montegut, L., Joseph, A., Chen, H., Abdellatif, M., Ruckenstuhl, C., Motino, O., Lambertucci, F., Anagnostopoulos, G., Lachkar, S., Dichtinger, S., et al. (2023b). High plasma concentrations of acyl-coenzyme A binding protein (ACBP) predispose to cardiovascular disease: Evidence for a phylogenetically conserved proaging function of ACBP. Aging Cell *22*, e13751.
16. Harris, F.T., Rahman, S.M.J., Hassanein, M., Qian, J., Hoeksema, M.D., Chen, H., Eisenberg, R., Chaurand, P., Caprioli, R.M., Shiota, M., and Massion, P.P. (2014). Acyl-coenzyme A-binding protein regulates Beta-oxidation required for growth and survival of non-small cell lung cancer. Cancer Prev. Res. *7*, 748–757.
17. Jacob, J.B., Wei, K.C., Bepler, G., Reyes, J.D., Cani, A., Polin, L., White, K., Kim, S., Viola, N., McGrath, J., et al. (2023). Identification of actionable targets for breast cancer intervention using a diversity outbred mouse model. iScience *26*, 106320.
18. Duman, C., Yaqubi, K., Hoffmann, A., Acikgöz, A.A., Korshunov, A., Bendszus, M., Herold-Mende, C., Liu, H.K., and Alfonso, J. (2019). Acyl-CoA-Binding Protein Drives Glioblastoma Tumorigenesis by Sustaining Fatty Acid Oxidation. Cell Metab. *30*, 274–289.e5.
19. Cavalloni, G., Peraldo-Neia, C., Massa, A., Bergamini, C., Trentini, A., De Rosa, G., Daniele, L., Ciccosanti, F., Cervellati, C., Leone, F., and Aglietta, M. (2021). Proteomic analysis identifies deregulated metabolic and oxidative-associated proteins in Italian intrahepatic cholangiocarcinoma patients. BMC Cancer *21*, 865.
20. Montégut, L., Liu, P., Zhao, L., Pérez-Lanzón, M., Chen, H., Mao, M., Zhang, S., Derosa, L., Naour, J.L., Lambertucci, F., et al. (2024). Acyl-coenzyme a binding protein (ACBP) - a risk factor for cancer diagnosis and an inhibitor of immunosurveillance. Mol. Cancer *23*, 187.
21. Joseph, A., Moriceau, S., Sica, V., Anagnostopoulos, G., Pol, J., Martins, I., Lafarge, A., Maiuri, M.C., Leboyer, M., Loftus, J., et al. (2020). Metabolic and psychiatric effects of acyl coenzyme A binding protein (ACBP)/diazepam binding inhibitor (DBI). Cell Death Dis. *11*, 502.
22. Anagnostopoulos, G., Saavedra, E., Lambertucci, F., Motiño, O., Dimitrov, J., Roiz-Valle, D., Quesada, V., Alvarez-Valadez, K., Chen, H., Sauvat, A., et al. (2024). Inhibition of acyl-CoA binding protein (ACBP) by means of a GABA(A)Rgamma2-derived peptide. Cell Death Dis. *15*, 249.
23. Liu, M., Zhang, S.L., Cheng, J., Liu, Y., Wang, L., Shao, Q., Zhang, J., and Lin, S.M. (2005). Genes transactivated by hepatitis C virus core protein, a microarray assay. World J. Gastroenterol. *11*, 3351–3356.
24. Montegut, L., Chen, H., Bravo-San Pedro, J.M., Motino, O., Martins, I., and Kroemer, G. (2022). Immunization of mice with the self-peptide ACBP coupled to keyhole limpet hemocyanin. STAR Protoc *3*, 101095.
25. Tsuchida, T., Lee, Y.A., Fujiwara, N., Ybanez, M., Allen, B., Martins, S., Fiel, M.I., Goossens, N., Chou, H.I., Hoshida, Y., and Friedman, S.L. (2018). A simple diet- and chemical-induced murine NASH model with rapid progression of steatohepatitis, fibrosis and liver cancer. J. Hepatol. *69*, 385–395.
26. Miragaia, R.J., Gomes, T., Chomka, A., Jardine, L., Riedel, A., Hegazy, A. N., Whibley, N., Tucci, A., Chen, X., Lindeman, I., et al. (2019). Single-Cell Transcriptomics of Regulatory T Cells Reveals Trajectories of Tissue Adaptation. Immunity *50*, 493–504.e7.
27. Sałkowska, A., Karaś, K., Karwaciak, I., Walczak-Drzewiecka, A., Krawczyk, M., Sobalska-Kwapis, M., Dastych, J., and Ratajewski, M. (2020). Identification of Novel Molecular Markers of Human Th17 Cells. Cells *9*.
28. Walker, J.A., and McKenzie, A.N.J. (2018). T(H)2 cell development and function. Nat. Rev. Immunol. *18*, 121–133.
29. Wu, Y., Li, N., Shu, X., Li, W., Zhang, X., Lü, D., and Long, M. (2023). Biomechanics in liver regeneration after partial hepatectomy. Front. Bioeng. Biotechnol. *11*, 1165651.
30. Bravo-San Pedro, J.M., Sica, V., Martins, I., Anagnostopoulos, G., Maiuri, C., and Kroemer, G. (2019a). Cell-autonomous, paracrine and neuroendocrine feedback regulation of autophagy by DBI/ACBP (diazepam binding inhibitor, acyl-CoA binding protein): the obesity factor. Autophagy *15*, 2036–2038.
31. Montegut, L., Joseph, A., Chen, H., Abdellatif, M., Ruckenstuhl, C., Martins, I., Madeo, F., and Kroemer, G. (2023a). DBI/ACBP is a targetable autophagy checkpoint involved in aging and cardiovascular disease. Autophagy *19*, 2166–2169.
32. Chen, F., Cai, X., Kang, R., Liu, J., and Tang, D. (2023). Autophagy-Dependent Ferroptosis in Cancer. Antioxidants Redox Signal. *39*, 79–101.
33. Zhou, B., Liu, J., Kang, R., Klionsky, D.J., Kroemer, G., and Tang, D. (2020). Ferroptosis is a type of autophagy-dependent cell death. Semin. Cancer Biol. *66*, 89–100.
34. Tomasetti, C., and Vogelstein, B. (2015). Cancer etiology. Variation in cancer risk among tissues can be explained by the number of stem cell divisions. Science *347*, 78–81.
35. Shalapour, S., and Karin, M. (2019). Pas de Deux: Control of Anti-tumor Immunity by Cancer-Associated Inflammation. Immunity *51*, 15–26.
36. Vesely, M.D., Kershaw, M.H., Schreiber, R.D., and Smyth, M.J. (2011). Natural innate and adaptive immunity to cancer. Annu. Rev. Immunol. *29*, 235–271.

CellPress
OPEN ACCESS

37. Hou, J., Karin, M., and Sun, B. (2021). Targeting cancer-promoting inflammation - have anti-inflammatory therapies come of age? Nat. Rev. Clin. Oncol. *18*, 261–279.

38. Gielchinsky, Y., Laufer, N., Weitman, E., Abramovitch, R., Granot, Z., Bergman, Y., and Pikarsky, E. (2010). Pregnancy restores the regenerative capacity of the aged liver via activation of an mTORC1-controlled hyperplasia/hypertrophy switch. Genes Dev. *24*, 543–548.

39. Toso, C., Patel, S., Asthana, S., Kawahara, T., Girgis, S., Kneteman, N.N., Shapiro, A.M.J., and Bigam, D.L. (2010). The impact of sirolimus on hepatocyte proliferation after living donor liver transplantation. Clin. Transplant. *24*, 695–700.

40. Ploumi, C., Papandreou, M.E., and Tavernarakis, N. (2022). The complex interplay between autophagy and cell death pathways. Biochem. J. *479*, 75–90.

41. Chen, X., Kang, R., Kroemer, G., and Tang, D. (2021). Broadening horizons: the role of ferroptosis in cancer. Nat. Rev. Clin. Oncol. *18*, 280–296.

42. Galy, B., Conrad, M., and Muckenthaler, M. (2024). Author Correction: Mechanisms controlling cellular and systemic iron homeostasis. Nat. Rev. Mol. Cell Biol. *25*, 671.

43. Tang, D., Kroemer, G., and Kang, R. (2024). Ferroptosis in immunostimulation and immunosuppression. Immunol. Rev. *321*, 199–210.

44. (2018). EASL Clinical Practice Guidelines: Management of hepatocellular carcinoma. J. Hepatol. *69*, 182–236.

45. Schulze, K., Imbeaud, S., Letouzé, E., Alexandrov, L.B., Calderaro, J., Rebouissou, S., Couchy, G., Meiller, C., Shinde, J., Soysouvanh, F., et al. (2015). Exome sequencing of hepatocellular carcinomas identifies new mutational signatures and potential therapeutic targets. Nat. Genet. *47*, 505–511.

46. Bravo-San Pedro, J.M., Sica, V., Martins, I., Pol, J., Loos, F., Maiuri, M.C., Durand, S., Bossut, N., Aprahamian, F., Anagnostopoulos, G., et al. (2019c). Acyl-CoA-Binding Protein Is a Lipogenic Factor that Triggers Food Intake and Obesity. Cell Metab. *30*, 754–767.e759.

47. Ip, B.C., Hu, K.Q., Liu, C., Smith, D.E., Obin, M.S., Ausman, L.M., and Wang, X.D. (2013). Lycopene metabolite, apo-10’-lycopenoic acid, inhibits diethylnitrosamine-initiated, high fat diet-promoted hepatic inflammation and tumorigenesis in mice. Cancer Prev. Res. *6*, 1304–1316.

48. Park, E.J., Lee, J.H., Yu, G.Y., He, G., Ali, S.R., Holzer, R.G., Osterreicher, C.H., Takahashi, H., and Karin, M. (2010). Dietary and genetic obesity promote liver inflammation and tumorigenesis by enhancing IL-6 and TNF expression. Cell *140*, 197–208.

49. Lambertucci, F., Motiño, O., Pérez-Lanzón, M., Li, S., Plantureux, C., Pol, J., Maiuri, M.C., Kroemer, G., and Martins, I. (2024). Isolation of Primary Mouse Hepatocytes and Non-Parenchymal Cells from a Liver with Precancerous Lesions. Methods Mol. Biol. *2769*, 109–128.

50. Kleiner, D.E., Brunt, E.M., Van Natta, M., Behling, C., Contos, M.J., Cummings, O.W., Ferrell, L.D., Liu, Y.C., Torbenson, M.S., Unalp-Arida, A., et al. (2005). Design and validation of a histological scoring system for nonalcoholic fatty liver disease. Hepatology *41*, 1313–1321.

STAR★METHODS

KEY RESOURCES TABLE

REAGENT or RESOURCE	SOURCE	IDENTIFIER
Antibodies		
Anti-ACBP/DBI antibody	Abcam	RRID: AB_231910
Anti-POR antibody	Abcam	RRID: AB_180597
Anti-KEAP1 antibody	Abcam	RRID: AB_119403
Anti-CHAC1 antibody	Abcam	RRID: AB_217808
Anti-NCOA4 antibody	Abcam	RRID: AB_86707
Anti-ACSL4 antibody	Abcam	RRID: AB_234167
Anti-ACSL3 antibody	Abcam	RRID: AB_151959
Anti-GPC3 antibody	Thermo Fisher Scientific	Cat# MA5-17083
Anti-BMAL1 antibody	Abcam	RRID: AB_93806
Anti-Ki67 antibody	Abcam	RRID: AB_119403
Anti-PCNA antibody	Abcam	RRID: AB_217808
Anti-alpha 1 Fetoprotein antibody	Abcam	RRID: AB_86707
Anti- Cytokeratin 19 antibody	Abcam	RRID: AB_52625
Anti-c-Myc antibody	Abcam	RRID: AB_32072
Anti-β-Catenin antibody	Abcam	RRID: AB_32572
Anti-GPX4 antibody	Cell Signaling Technology	Cat# 52455
Anti-GAPDH antibody	Cell Signaling Technology	Cat# 2118
Anti-MAP1LC3B antibody	Cell Signaling Technology	Cat# 2775
Anti-p62/SQSTM1 antibody	Abnova	Cat# H00008878-M01
Goat Anti-Rat IgG (H + L) secondary antibody	Southern Biotech	Cat# 3050–05
Goat Anti-Rabbit IgG (H + L) secondary antibody	Southern Biotech	Cat# 4050–05
IgG2a antibody (*in vivo* isotype control)	Bioxcell	Cat# BE0085
Monoclonal anti-ACBP/DBI (*in vivo* neutralization)	Fred Hutch Antibody Technology	N/A
Biological samples		
Human plasma from HCC patients	Samples provided by Prof. Jessica Zucman-Rossi	Centre de Recherche des Cordeliers, Inserm U1138,Team "Functional Genomics of solid Tumors"
Chemicals, peptides, and recombinant proteins		
Imidazole ketone erastin(IKE)	SelleckChem	100mg
Erastin	Sigma Aldrich	E7781-1M
1S,3R-RSL 3	Sigma Aldrich	SML2234-25MG
Blasticidin (solution)	Invivogen	ant-bl-05
Linoleic acid	Sigma Aldrich	L1012-1G
Linolenic acid	Sigma Aldrich	L2376-500MG
Arachidonic Acid	Sigma Aldrich	181198-100MG
Albumin Bovine FrV BSA	Euromedex	04-100-812-E
Poly(ethylene glycol)	Sigma Aldrich	202371-250G
TWEEN® 80	Sigma Aldrich	P1754-500ML
Imject mcKLH Subunits	Thermo Fisher Scientific	77649
Montanide ISA 51 VG	SEPPIC	36362/FL2R3
Recombinant mouse ACBP/DBI	Custom-made	N/A

(*Continued on next page*)

Continued

REAGENT or RESOURCE	SOURCE	IDENTIFIER
QIAzol Lysis Reagent (200 mL)	QIAGENE	79306
SuperSignal West Pico chemiluminescent substrate	Thermo Fisher Scientific	34579
SYBR Green Master Mix	Applied Biosystems	4367659
Tamoxifen Free Base	Sigma Aldrich	T5648
Firefly luciferase lentivirus	GenTarget Inc	LVP568-PBS
Polybrene	Sigma Aldrich	TR-1003-G
Beetle Luciferin, Potassium Salt, 1g	Promega	E1605
Percoll^{TM}	Cytiva	17-0891-01
Laminine	Sigma Aldrich	11243217001
Collagénase/dispase®	Sigma Aldrich	10269638001
Red Blood Cell Lysis Buffer	Sigma Aldrich	11814389001
ITS+1 Liquid Media Supplement (100×)	Sigma Aldrich	I2521-5ML
EGFh	Sigma Aldrich	E9644-.2MG
IGF-II human	Sigma Aldrich	SRP3070-50UG
Entellan rapid mouting medium, with Xylene	Sigma Aldrich	PN 1.07961.0100
Mayer's hemalum solution	Sigma Aldrich	PN 1.09249.0500
Eosine – Y in aqueous solution 0.5%	VWR	PN 10047001
D(–)-Fructose	Sigma Aldrich	F0127-1KG
D-(+)-Glucose	Sigma Aldrich	G8270-5KG
Carbon tetrachloride (CCl_4)	Sigma Aldrich	289116-100ML
Corn oil	Sigma Aldrich	C8267-500ML
**Critical commercial assays**		
Cell counting kit-8	Sigma Aldrich	96992-500TESTS-F
miRNeasy Mini Kit (50)	Qiagen	217004
SuperScript^{TM} IV VILO^{TM} Master Mix with ezDNase^{TM} Enzyme	Invitrogen	11766500
Visium CytAssist for FFPE Spatial Gene Expression 6.5mm, Mouse, 4 rxns	10x Genomix	PN-1000251
Dual Index Kit TS Set A, 96 rxn	10x Genomix	PN-1000251
MojoSort^{TM} Mouse CD3 T cell Isolation Kit	BioLegend	Cat# 480024
MojoSort^{TM} Biotin anti-mouse CD19 Antibody	BioLegend	Cat# 152420
MojoSort^{TM} Mouse Pan Dendritic Cell Isolation Kit	BioLegend	Cat# 480097
**Deposited data**		
Mouse (MASH-driven HCC) bulk RNA sequencing	NCBI Gene expression omnibus (GEO)	GEO: GSE255799
Mouse (MASH-driven HCC) spatial transcriptomics	NCBI Gene expression omnibus (GEO)	GEO: GSE296545
Mouse (MASH-driven HCC) spatially resolved mass spectrometric imaging (MSI)-based metabolomics	Metabolomics Workbench	ST003957
**Experimental models: Cell lines**		
HUH-7	Cells provided by Dr. Patrick Soussan	N/A
HEP-G2	ATCC	HB-8065
Hep55.1C	Cytion	400201
Mouse primary MASH-induced HCC cell lines	This paper	N/A

(*Continued on next page*)

Continued		
REAGENT or RESOURCE	SOURCE	IDENTIFIER
Experimental models: Organisms/strains		
ACBP/DBI $^{fl/fl}$ mice in which *loxP* sites flank *ACBP* exon 2	Ozgene	N/A
Gabrg2tm1Wul/J GABAA g2$^{+/+}$ mice	Charles River Laboratory, Lentilly, France	N/A
Gabrg2tm1Wul/J GABAA g2$^{F77I/F77I}$ mice	Charles River Laboratory, Lentilly, France	N/A
Liver-specific *ACBP/DBI* $^{fl/fl}$ mice in which *loxP* sites flank *ACBP* exon 2	Ozgene	N/A
Oligonucleotides		
Mouse qRTPCR		N/A
Primer: 36b4<br>F: 5′-ACTGGTCTAGGACCCGAGAAG-3′<br>R: 5′-TCCCACCTTGTCTCCAGTCT-3′	Sigma-Aldrich	N/A
Primer: Abcc5<br>F: 5′-GCAAACTGGTTGGAATCTGCGG-3′<br>R: 5′-CAAAGGTCCCACTGACGGCAAT-3′	Sigma-Aldrich	N/A
Primer: Acsl3<br>F: 5′-GCGAGAAGGATTCCAAGACTGG-3′<br>R: 5′-GAAGAGTAGCCGATTCGGCATC-3′	Sigma-Aldrich	N/A
Primer: Acsl4<br>F: 5′-CCTTTGGCTCATGTGCTGGAAC-3′<br>R: 5′-GCCATAAGTGTGGGTTTCAGTAC-3′	Sigma-Aldrich	N/A
Primer: Aldh3a2<br>F: 5′-GAGACCGGCTAAGAAGAACCT-3′<br>R: 5′-CGAAAGGGTAATTCCAAGCTCC-3′	Sigma-Aldrich	N/A
Primer: Arntl<br>F: 5′-ACCTCGCAGAATGTCACAGGCA-3′<br>R: 5′-CTGAACCATCGACTTCGTAGCG-3′	Sigma-Aldrich	N/A
Primer: Atr<br>F: 5′-GAATGGGTGAACAATACTGCTGG-3′<br>R: 5′TTTGGTAGCATACACTGGCGA-3′	Sigma-Aldrich	N/A
Primer: Ccnb1<br>F: 5′-CTTGCAGTGAGTGACGTAGAC-3′<br>R: 5′-CCAGTTGTCGGAGATAAGCATAG-3′	Sigma-Aldrich	N/A
The list of additional mouse and human oligonucleotides used in this study is provided in Table S2.		
Software and algorithms		
ImageJ	National Institutes of Health	https://imagej.nih.gov/ij/notes.html
GraphPad Prism 8 software	Graph Pad Software Inc	https://www.graphpad.com/features
QuPath 0.2.3 software	Center for Cancer Research & Cell Biology at Queen's University Belfast	https://qupath.github.io/
StepOne Software v2.3	Applied Biosystems Inc	https://stepone-software.software.informer.com/2.3/
Zen 3.2 software	ZEISS	https://www.zeiss.com/microscopy/en/home.html
**BD FACSDiva v8.0.2**	BD Biosciences	https://www.bdbiosciences.com/en-fr/products/software/instrument-software/bd-facsdiva-software
OMIQ	Dotmatics	https://www.omiq.ai/
R software (4.2.1; 4.3.3)	Posit, PBC	https://www.R-project.org/
MetaXpress® Image software v6.6.3.55	Molecular Devices	N/A

(*Continued on next page*)

Continued

REAGENT or RESOURCE	SOURCE	IDENTIFIER
Other		
42%Kcal/Fat Diet (incr.Sucrose 12,5% Chol) Teklad	Envigo	MD.120528
Precellys tissue homogenizing ceramic beads	Bertin Technologies	CKMix
Lithium heparin blood collection tubes	Sarstedt	6443
Poches Transparentes Sipper Sack	Avidity Science	2204–002117
Corning® cell strainer 100uM	Corning	CLS431752-50EA
Corning® cell strainer 70uM	Corning	CLS431751-50EA
Corning® cell strainer 40uM	Corning	CLS431750-50EA
Polysine adhesion microscope slides with white tab	Epredia	PN J2800AMNZ
Cover splits 22x50mm	Epredia	PN BB02200500A113MNZ0
Slide mailer 5 places with side open	VWR	PN 631-1515
Histosette I	Simport	PN M490-3
Biopsy foam pads square	Simport	PN M476-1

EXPERIMENTAL MODEL AND STUDY PARTICIPANT DETAILS

HCC patient cohort

A total of 260 plasma samples were collected from 146 patients with HCC and 58 patients with chronic liver diseases without HCC at the Avicenne tertiary University Hospital (Bobigny, France) between March 2013 and May 2021. Two or more plasma samples were collected from 46 patients. Plasma samples were divided into two groups: (i) plasma collected at the time of diagnosis of HCC, the day of treatment or at the time of radiological evaluation showing active HCC (n = 195), (ii) plasma collected in patients with chronic liver disease without HCC or plasma collected after HCC treatment without any active tumor at imaging (n = 65). Whole blood (5 mL) was collected using EDTA tubes. Blood samples were centrifuged at 2000 $\times$g for 10 min at room temperature and were immediately stored at -80°C. All patients signed an informed consent for sample collection and the ethics committee approved the study (CCPPRB Paris Saint-Louis IRB00003835).

Patient, tumor characteristics, and treatment types were obtained from medical records. Patient baseline characteristics were collected before treatment: including age, gender, etiology of liver disease, and presence of cirrhosis. Cirrhosis was defined either by histology or by the combination of clinical-biological data, ultrasonography, and liver stiffness measurement (Fibroscan). HCC was diagnosed at histology or using non-invasive criteria through imaging techniques (magnetic resonance imaging [MRI] and/or triphasic computed tomography [CT]) according to the European Association for the Study of the Liver (EASL) guidelines.[44] Tumor characteristics at imaging (tumor size and number, macrovascular invasion, and metastasis), Barcelona Clinic liver cancer staging system (BCLC), alpha-fetoprotein (AFP), and Child-Pugh (CP) score were also collected. Data about the type of treatment (ablation, embolization, and/or systemic treatment), and radiological response assessed by mRECIST criteria at 4 and 12 weeks after locoregional and systemic treatment respectively, were also recorded.

An independent HCC series from Functional Genomics of Solid Tumors, Center de recherche des Cordeliers (Paris, France) was used to evaluate the prognostic value of DBI in HCC.[45]

Animals

All mice (males and females, aged 2–7 weeks) used in this study had a C57BL/6 background. Five mice/cage were housed in a 12 h light/dark cycle under specific pathogen-free conditions. Mice were allowed at least 1 week of acclimation to the new housing facilities prior to use. All mouse experiments were performed according to protocols approved by the local Animal Experimental Ethics Committee (protocols #25000, #25355, #10862, #8530, #25010, #0447.02, #31411, #34537, #34538, and #34539; 490/2019-PR).

METHOD DETAILS

Generation of stable cell lines, cell culture, and cell assays

Generation of stable luciferase-expressing Hep55.1C cells

Hep55.1C cells (2×10^5 per well in 6-well plates) were cultured overnight in 2 mL full medium (DMEM, 10% FBS). Then 10 µL firefly luciferase lentivirus (GenTarget Inc, LVP568-PBS) was added at an MOI of 10 and 8 µg/mL Polybrene (Sigma, TR-1003-G). After 72h of culture, the virus-containing media were replaced by fresh full medium containing 10 µg/mL blasticidin, and media were replaced every 2–3 days until cells cultures reached ~70% confluency before expansion in T25 flasks. Single cell sorting was performed in

96-well plates. Around 30 days clones were expanded, and the expression of luciferase was validated. For this, ~5 × 10^3cells/well were seed in 96-well plate, 150 μg/mL D-Luciferin (Promega, E1605) was added for 5min, and bioluminescence was measured.

Generation of stable DBI knockdown liver cancer cell lines

Liver cancer cells were grown under the following conditions: HEP-G2 (EMEM +10% FBS +1% sodium pyruvate +1% HEPES), HUH-7 (DMEM, 10% FBS), Hep55.1C (DMEM, 10% FBS) and Hep55.1C cells expressing firefly luciferase (Hep55.1C-luc, DMEM, 10% FBS, 0.1% blasticidin). These cancer cells were grown in 6-well dishes to 60–70% confluency then transfected using 25–35 μL lentiviral shRNAs targeting DBI (SH1, SH2, SH3) or negative control (NC) with 5 μg/mL polybrene (5–8 μL) mixed thoroughly in 1mL medium. The medium was replaced with fresh medium after 24–48 h, and the cells were maintained for another 24 h. Puromycin (10 μg/mL) was used to select the transduced cells. Single cell clones were isolated by single-cell FACS sorting in 96-well plates and gradually expanded in 24-well plates, 12-well plates and 6-well plates. All the cell clones from 6-well plates were duplicated, one stocked at −80°C for stock and one collected for qRT-PCR to detect the knockdown efficiency of DBI. Clones with the highest knockdown efficiency were chosen to perform further assays.

Proliferation assays

CCK-8 assays were performed with a commercial kit (Cell Counting Kit-8, Sigma-Aldrich) according to the manufacturer's instructions. For colony formation assays, HEPG-2 (NC, SH1, SH2, and SH3, 1000 cells/well), HUH-7 (NC, SH1, SH2, and SH3, 1000 cells/well) and Hep55.1C (NC, SH1, SH2, and SH3, 500 cells/well) cancer cells were seeded into 6-well plates in 2 mL medium with 10% FBS. The medium was changed every 3 days until control (NC) wells approached confluency, within 7–14 days. After gently washed twice with PBS, colonies were fixed with 4% paraformaldehyde for 30 min and stained with 0.2% crystal violet for 30min at room temperature. The colony number (colonies >0.3 mm) was counted with ImageJ software. All experiments were performed in triplicates.

Cell cycle analysis

Following the instructions of a commercial kit (Cell Cycle Analysis Kit, Sigma-Aldrich), the aforementioned transfected cells (25x10^4 cells/well) were plated in 6-well plates for 24 h. Cell cycle was then synchronized with medium containing 0.1% FBS for 24h, followed by culture in medium with 10% FBS for 24 h. The cells were harvested and fixed using ice-cold 70% ethanol. The cell cycle distribution was then determined by flow cytometry.

Reverse siRNA transfection

Hep55.1C cells were transfected with *Gabrg2*-targeting siRNA (SMARTpool, Dharmacon, Horizon Discovery, Cat# M-045579-01-0010) using lipofectamine (RNAiMAX, Invitrogen, Cat# 13778150) following the manufacturers's instructions. The culture medium was replaced 24 h post-transfection, and cells were harvested 48 h later for gene knockdown validation by qRT-PCR.

Inhibition of ACBP/DBI *in vivo*

Four strategies were developed to inhibit the expression or function of ACBP/DBI. (i)*Tamoxifen-inducible ACBP/DBI knockout*. Mice with tamoxifen-inducible ACBP/DBI knockout were generated by crossing *ACBP/DBI*$^{fl/fl}$ mice (obtained from OZgene) with *B6.Cg-Tg (UBC-Cre/ERT2)1Ejb/1J* mice (obtained from Jackson laboratory, Bar Harbor, ME, USA), followed by tamoxifen injection (*i.p.*75 mg/kg/day, for 5 consecutive days). Control mice were *ACBP/DBI*$^{fl/fl}$ mice without Cre. Tamoxifen (Sigma-Aldrich) was dissolved in corn oil (90%) + ethanol (10%) at a concentration of 20 mg/mL, aliquoted and stored at −20°C. (ii) *Constitutive Gabrg2*$^{F77I/F77I}$ *point mutation. Gabrg2*^{tm1Wul}*/J* mice (obtained from Charles River Laboratory, Lentilly, France) containing the point mutation F77I were compared to control mice with wild type mice without the mutation. (iii) Induction of ACBP/DBI-specific autoantibodies. As previously described,[8,24,30,46] KLH was conjugated with recombinant ACBP at 1:30 molar ratio to produce KLH-ACBP. KLH alone was used as control. Montanide (Seppic, Paris, France) and KLH/KLH-ACBP were then mixed a 1:1 volume ratio. The mice were i.p. vaccinated with the aforementioned mixture on days 0 (30 μg), 7 (30 μg), 14 (30 μg), and 21 (10 μg of KLH-ACBP) to induce anti-ACBP autoantibodies. Immunoblots of recombinant ACBP were incubated with plasma from KLH/KLH-ACBP-immunized mice to detect anti-ACBP antibody. (iv) Monoclonal anti-ACBP antibody. Mice were i.p. injected with anti-ACBP mAb (a-ACBP, 5mg/kg) or its control isotype (5mg/kg, Bioxcell, NH, USA) 3–4 times/week to neutralize ACBP.

Liver tissues and plasma samples were collected for further analysis. Liver tissues were freshly frozen in liquid nitrogen and stored at −80°C, fixed in 4% paraformaldehyde, or embedded in optimum cutting temperature compound (OCT).

MASH-driven HCC model

Three major types of HCC mouse models were tested in this study. Diet plus toxin-induced mouse models were based on a western diet (WD) or high-fat diet (HFD) plus carbon tetrachloride (CCl_4) or diethylnitrosamine (DEN), respectively. In most experiments, mice received a western diet (WD, i.e., a high-fat, high-fructose and high-cholesterol diet, MD.120528, Envigo, Paris, France), high-sugar water (23.1 g/L D-fructose plus 18.9 g/L D-glucose), and weekly i.p. injections of carbon tetrachloride (CCl_4, 1:10 diluted in corn oil, Sigma-Aldrich, Darmstadt, Germany) at a final dose of 2 μL/g of body weight.[25] High-sugar water was added in the sterile 450mL sipper sack bags and replaced weekly. Also, food was added weekly. Male mice were used in this HCC model. Four ACBP/DBI inhibition strategies (conditional whole body *DBI* knockout, *Gabrg2*$^{F77I/F77I}$ mutation, KLH-ACBP vaccination, and liver-specific *DBI* knockout) were used. The detailed experimental designs are depicted in Figures 4A, 4G, 4M, 4S, and S8A. Mice were sacrificed after 27–49 weeks, based on hepatic tumor detection by means of medical ultrasound (also called sonography). At necropsy, tumor number and size were determined by counting the number of visible tumors and measuring their size with a caliper. Body weights of mice

CellPress
OPEN ACCESS

were monitored weekly. HFD-DEN induced HCC mouse model (combined with KLH-ACBP) was used to confirm data from WD plus CCl_4 mouse model. For this, male C57BL/6 mice (15 dpp) were treated with a single dose of diethylnitrosamine (DEN, N0258, Merck, Darmstadt, Germany) dissolved in saline at a dose of 25 mg/kg body weight by intraperitoneal injection.[47,48] After 2 weeks, concomitant with the weaning, mice started the vaccination protocol and HFD composed by 60% fat calories (Bioserv F3282, Flemington, NJ, US). Fresh diet was provided every 2 to 3 days and body weights were recorded monthly. Mice were sacrificed after 36 weeks and livers explanted to measure tumor number and size. Vaccination was performed with KLH/KLH-ACBP. Specifically, for the vaccination protocol, DEN injected mice (28 dpp) were randomized in two groups.

Oncogene-induced HCC

Hydrodynamic transfection of oncogenes (*Myc+Ctnnb1*). A mixture of transposase-encoding vector (SB100, 1.5 μg), pT3-EF1a-Myc plasmid (7.5 μg), and pT3-N90-Ctnnb1 plasmid (7.5 μg) were prepared fresh in 1mL 1xPBS at a ratio 1:5:5. The final solutions was sterile-filtrated through a 0.22 μm filter and placed in a 37°C water bath prior to use. A volume equivalent to 10% of mouse body weight (1mL/10g) was injected via tail veins with high pressure within 8-10s, using a 3 mL syringe with 30G x 1/2 needles in female mice (7 weeks-old). Combinations of the aforementioned four ACBP/DBI inhibition strategies and this HCC mouse models were tested to and tumor formation were monitored with ultrasound. The survival was monitored and mice were sacrificed at the humane endpoint, defined by mouse grimace scale, notable abdominal distension caused by tumor burden, >20% initial body weight loss, or largest tumor size reaching 20 mm based on ultrasound. The tumor number and size were measured and recorded at necropsy. To determine the effect of ACBP/DBI blockade on Myc and β-catenin, anti-ACBP was administered intraperitoneally at 5 mg/kg once per day for one week. At day 8, the livers were collected and protein was extracted from the whole liver to detect the expression of Myc and β-catenin by immunoblotting.

Orthotopic transplantation of HCC cells

Murine HCC cell lines (Hep55.1C and Hep55.1C-derived cell lines such as Hep55.1C-Luc, or Hep55.1C/Hep55.1C-Luc-NC, -SH1, -SH2, and -SH3, $30x10^4$/50μL/mouse in DMEM without FBS) and human HCC cells (HUH-7, $200x10^4$/50μL/mouse in DMEM without FBS) were transplanted into mouse liver to generate orthotopic liver tumors in female C57BL/6J mice or male nude mice, respectively. Tumor growth of mouse models implanted with luciferase-labeled Hep55.1C-Luc was monitored by IVIS bioluminescence *in vivo* imaging system, otherwise via ultrasound unless noted. Mice were monitored for survival and symptoms (such as skin jaundice). Mice were sacrificed at the humane endpoints mentioned above.

Partial hepatectomy

9 weeks-old male mice were submitted to a 70% partial hepatectomy (Ph). Briefly, mice were administered with buprenorphine for analgesia and anesthetized by inhalation of isoflurane (2%). Then, a midline abdominal skin and muscle incision was made to expose the peritoneal cavity, followed by the removal of the left and median hepatic lobes. First, a 4-0 silk thread was placed on the base of the left lateral lobe, the knot was tied and the tied lobe was cut just above the suture. The same procedure was done with the median lobe. Finally, the peritoneum was closed with a 5-0 suture and the skin with wound clips. Sham controls mice were submitted to the same surgical procedure without removing the liver lobes. Mice were randomized into four groups (Sham+Isotype, Sham+anti-ACBP, Ph+Isotype and Ph+anti-ACBP), 9–10 mice/group. Anti-Acbp mAb (i.p. 2.5mg/kg) or Isotype (i.p. 2.5mg/kg, Bioxcell, NH, USA) were administered 4h and 1h before the surgery and three more times before sacrifice. Seven days after the partial hepatectomy, animals were sacrificed, and the remaining liver was harvested and processed for further analysis.

Combination therapies

Anti-ACBP plus anti-PD-1

Hep55.1C cells ($3x10^5$/50μL/mouse in DMEM without FBS) were inoculated into the liver of 7-week-old female C57BL/6J mice. Mice were randomized into four groups (isotype1+isotype2, isotype1+anti-PD1, anti-ACBP+isotype2, and anti-ACBP+anti-PD1), 9–10 mice/group, 10 days after tumor initiation. Treatment with anti-ACBP mAb (i.p. 5 mg/kg) or isotype1 (i.p. 5 mg/kg, Bioxcell, Lebanon, NH, USA) was then started and administered 3 times/week. Treatment of anti-PD1/isotype2 (i.p. 200μg/mice) started at the week after 6-time dosing of anti-ACBP/isotype1. Mice were sacrificed at the humane endpoint mentioned above. In Figures 5 and 7, Iso represents isotype, and αACBP/αPD1 indicate anti-ACBP/anti-PD1, respectively. Similarly, effect of anti-ACBP/anti-PD1 on immune infiltration was investigated in this model by means of Flow cytometry analysis. The exact experiment design referred to Figure S9A and the details for flow cytometry analyses of the immune infiltration shown below.

Anti-ACBP plus ferroptosis inducers

Hep55.1C-Luc cells ($3x10^5$/50μL/mouse in DMEM without FBS) were inoculated into the liver of 7-week-old female C57BL/6J mice. The tumors were assessed by *in vivo* bioluminescence imaging using the IVIS system (*i.p.* injection of 200 μL 15 mg/mL luciferin/mice 5 min before detection) at day 7 after tumor initiation. The mice were divided into 4 groups (isotype+vehicle, isotype+RSL3/IKE, anti-ACBP+vehicle, anti-ACBP+RSL3/IKE) based on the bioluminescence imaging. Treatment with anti-ACBP/isotype (*i.p.* 5 mg/kg) was then started with a regular dosing schedule, 2 days on/1 day off for 8 out of 10 days. RSL3 and IKE were administered at 50 mg/kg for 4 times in 2 continuous weeks, started at day 21/19 from tumor inoculation. Tumor growth was monitored for 4 weeks. Mice were sacrificed at endpoints mentioned above.

Isolation of primary HCC cells

Six-well plates were coated with 2%FBS/PBS+0.1% laminin. HCC tumors were isolated from the MASH-driven mouse model (WD + D-fructose+D-glucose in water+CCl_4 administered to $DBI^{+/+}$ or $DBI^{-/-}$ mice) and washed twice with ice-cold PBS. The tumor tissues were transferred to 10 cm plates, minced completely with a razor blade and then transferred to 15 mL falcon tubes. Ten mL 2 mg/mL collagenase dispase solution was added, the cells were incubated rotating for 30 min at 37°C and were filtered through 100 μm meshes into 50 mL falcon tubes, before addition of 10 mL 2%FBS/PBS to wash the tissues. The samples were then filtered sequentially through 70 μm and 40 μm strainers, brought up to 15 mL volume and centrifuged again (room temperature (RT), 1000 rpm, 2 min). The cells were then resuspended in 5 mL of 1× red blood cell (RBC) lysis buffer, incubated on ice for 10 min before addition of 5 mL 2%FBS/PBS, followed by spinning (RT, 1000 rpm, 2 min). The washing step was repeated 2 times, and the cells were resuspended in culture medium (500 mL DMEM +10% FBS+1×ITS +20μL EGF (1 μg/μL) + 20 μL IGFII (0.2 μg/μL)), before seeding into 6-well plates.

Isolation and purification of primary mouse hepatocytes and immune cells

Isolation of primary hepatocytes and Kupffer cells

Hepatocytes were isolated from 11-week-old female C57BL/6 mice as described.[49] Simultaneously, Kupffer cells were isolated. For this, the digested liver cell suspension was filtered through a 100 μm straine, and centrifuged at 50 × g for 2 min at 4°C. The pellet was sued for the purification of hepatocytes and the supernatant, which contains non-parenchymal cells (NPCs), for the purification of Kupffer cells. For the enrichment of NPCs, the supernatant was centrifuged at 300 × g for 5 min at 4°C, and the pellet was resuspended in 1x HBSS with 2% FBS. Percoll gradient centrifugation was then performed to collect Kupffer cells. Isotonic Percoll solutions (50% and 25%) were prepared by diluting Percoll (Cytiva, 17-0891-01) with 10× HBSS and distilled water. The 25% Percoll was carefully layered over 50% Percoll in a 15 mL conical tube, and the NPC suspension was gently added on top of the 25% Percoll layer and centrifuged at 800 × g for 20 min at 4°C without braking. After centrifugation, the interphase layer (between 25% and 50% Percoll) was collected by means of a pipette, transferred to a fresh tube, diluted with 1x HBSS containing 2% FBS, centrifuged at 300 × g for 5 min at 4°C. Finally, the pellet was resuspended in QIAzol lysis reagent for RNA extraction to proceed to qRT-PCR.

Isolation and purification of T cells, B cells and dendritic cells

Isolation and purification of T and B lymphocytes, and dendritic cells (DCs) were performed using MojoSort mouse immune cell isolation kits (BioLegend, Cat: 480024, 152420, and 480097) from the spleen and lymph nodes of 11-week-old female C57BL/6J mice. Harvested tissues were placed in cold RPMI-1640, mechanically dissociated, and filtered through a 70 μm strainer. The suspension was adjusted to 5 mL with RPMI-1640, filtered again, and centrifuged at 400 × g for 5 min at 4°C, after which the supernatant was discarded. For spleen samples, cells were resuspended in 1× RBC Lysis Buffer, incubated at room temperature for 5 min, then washed with MACS buffer (1× PBS with 0.5% BSA and 2 mM EDTA), centrifuged at 400 × g for 5 min at 4°C, and the supernatant was discarded. The cells were resuspended in MACS buffer and divided into three aliquots per mouse for T cell, B cell and DC isolation. Each aliquot was incubated with 10 μL of the respective biotin-antibody cocktail on ice for 15 min, followed by washing with 5 mL of MACS buffer and centrifugation at 300 × g for 5 min at 4°C. After discarding the supernatant, 10 μL of streptavidin nanobeads were added per tube, mixed and incubated on ice for 15 min. The cells were then washed twice with 5 mL of MACS buffer, centrifuged at 300 × g for 5 min at 4°C and resuspended in 1 mL of MACS buffer. For magnetic separation, an LS column was placed onto a MultiMACS Cell24 Separator Plus single-column adapter, and a 40 μm MACS filter was positioned on top. The column was prerinsed with 2 mL of MACS buffer before applying the resuspended negative fraction onto the LS column. The column was washed three times with 1 mL of MACS buffer, following the MultiMACS Cell24 Separator Plus user manual. Correctly labeled 5 mL tubes were placed in the MultiMACS Cell24 Separator Plus 5 mL tube rack on the elution chamber, and plugged columns were positioned to ensure a vacuum seal. The single-column adapter with the columns was placed on top of the elution chamber, and the system was operated according to on-screen instructions. To elute the cells, 1 mL of MACS buffer was added to each column, and collection tubes were retrieved. The eluted samples were centrifuged at 300 × g for 5 min, and the pellets were resuspended in QIAzol lysis reagent for RNA extraction and later qRT-PCR.

Multi-omics analysis

Bulk RNA-sequencing

Murine liver tissue samples were homogenized in QIAzol lysis reagent (Cat. 79306, QIAGEN, Hilden, Germany). Total RNA was extracted and purified by means of the miRNeasy Mini Kit (Cat. 217084, Qiagen), following the manufacturer's protocol. Total RNA was subjected to RNA sequencing on a NovaSeq 6000 PE150 instrument by Novogene (London, UK). The RNA sequencing data were obtained in Fasta file format. Reads were mapped to mouse genome assembly (GRCm39, mm10) using HISAT2 (Version 2.2.1), followed by read counting with HTSeq-count (Version 2.0.2). The differential expression analysis was estimated using DESeq2 R-package (1.36.0). We used variance stabilizing transformation (VST) to transform and normalize the count matrix as implemented in DESeq2. Gene set enrichment analysis (GSEA)-based KEGG pathway and gene ontology (GO), including biological process (BP), molecular function (MF), and cellular component (CC), were performed with the differential expression data using WebGestalt, a web-based tool for functional enrichment analysis. R software (4.2.1), as well as ggplot2[3.3.6] and Complex Heatmap[2.13.1] packages were used to visualize data.

CellPress
OPEN ACCESS

Spatial transcriptomic

Tissue samples were initially screened for RNA quality, ensuring DV200 scores exceeded 50% (RNA 6000 Nano kit, Part number 5067–1511, Agilent, Santa Clara, CA USA), as recommended by the Visium Tissue Preparation Guide (10X Genomics, Pleasanton, CA, USA). The Visium spatial gene expression slides and reagent kits were employed following the manufacturer's instructions (User Guide, CG000495 Rev C, Product number 1000520). Five-micrometer thick tissue sections were cut from the FFPE tissue blocks and mounted on Superfrost Plus slide (VWR, Radnor, PA, USA). The sections underwent initial deparaffinization, imaging and permeabilization steps (User Guide, CG000520 Rev B) before being meticulously hybridized with the probes. Then, probes pairs that had successfully hybridized to RNA are ligated to facilitate the sealing of junctions between them and finally released from the tissue within the Visium CytAssist instrument for capture on the FFPE Visium Spatial Gene Expression Slide within the fiducial frame.

Each capture area comprised approximately 5000 gene expression spots, that include an Illumina TruSeq partial read 1 sequencing primer, 16 nucleotide (nt) Spatial Barcode, 12 nt unique molecular identifier (UMI), 30 nt poly(dT) sequence (captures ligation product). These spots offered a resolution of approximately 5–10 cells. Ligation products were extended by the addition of UMI, partial Read 1, and spatial barcodes, ultimately yielding spatially barcoded products for subsequent library preparation. qRT-PCR was meticulously employed to ascertain cycle numbers, and the ligated and spatially barcoded products underwent indexing via Sample Index PCR. The sequencing of libraries was executed on a Novaseq 6000 instrument with an SP flow cell (100 cycles, Illumina, CA, USA). The Space Ranger pipeline v2.1.1 (10×Genomics, Pleasanton, CA, USA) and the refdata-gex-mm10-2020-A reference were used to process FASTQ files and generate the.cloup files. tSNE and spatial plots were run and plotted using Loupe Browser (10× genomics). Then, the R package *seruat (version 5.0.0)* was used to conduct downstream analyses. We used *sctransform* to normalize the count data to remove technical variability. Ferroptosis driver and suppressor molecular scores were calculated as the average expression of genes involved in ferroptosis gene signature (referred to Figure 6A) and *p* values calculated by means of the Wilcoxon signed-rank test. Immunosuppression scores and immunostimulation scores were calculated as the average expression of genes shown in Table S1.

Spatial metabolomics

The details including sample preparation, H&E staining, mass spectrometry imaging (MSI) acquisition, and data analysis are described. *(i) Sample preparation for MSI.* Liver tissues were harvested from mice, rinsed with PBS and placed in embedding cassette with one piece of foam pads then gently frozen in steam bath of liquid nitrogen. Cassettes were stored at −80°C. Tissue sections were performed with a cryotome HM 500 O (Micro). Tissues were warmed from −80°C to −15°C in 20 min. The piece of tissue was mounted with milliQ water on a peltier platform, and the object was fast-frozen for 10 min. Sections of tissues were cut with a thickness of 20 μm and put on labeled microscope slides. Three slides were alternatively dedicated to the Mass Spectrometry Imaging (MSI) positive mode, MSI negative mode and Hematoxylin and Eosin (H&E) staining. MSI Mass spectrometry imaging (MSI) sections were frozen in slide mailers at −80°C and H&E staining was performed right after cutting. *(ii) H&E staining*. Sections on the microscope slide were successively bathed in 100%, 70% and 50% of ethanol (EtOH), each step during 2 min, then followed by 2 min in tap water. Tissues were afterward colored with hematoxylin for four 4.5 min, and rinsed with water for 4 min. Second coloration was done with eosin for 2.2 min, rinsed again with water for 1.5 min. Three last baths were applied on the sections, respectively at 50, 70 and 100% of EtOH, for 1 min of each. Finally, a quick rinse with Xylene was done before mounting cover slips with Entellan. *(iii) MSI acquisition*. Full scan (50 m/z to 1200 m/z) acquisitions were performed in sensitive mode with a synapt XS waters equipped with a DESI XS source. Three slices were acquired in positive polarity acquisition mode, and three others were used for negative polarity acquisition mode. Tissue sections acquired in negative polarity were sprayed with 2 μL/min of methanol/water buffer (96/4, with 1 mmol of ammonium acetate) with lockspray leucine-enkephaline (250 ng/mL). Capillary voltage was set at 0.70V, sampling cone at 40V, source temperature at 150°C. Regarding the negative polarity acquisition mode, the 2 μL/min of buffer was composed of methanol/water (82/18) with 0.1% of formic acid and 250 ng/mL of leucine-enkephaline. Capillary voltage was set at 0.40V, sampling cone at 40V, source temperature at 150°C. DESI source was scanning the tissue spatial resolution with a spatial resolution of 50 μm^2 per pixel, and a scan time of 0.153s. Acquisition parameters were set with HDI v1.6 and acquired with MassLynx v4.2. *(iv) Data analysis*. Raw data were processed by HDI v1.6. A target list was built from one replicate, based on the thousand most intense ion signals. Then we added to this data-driven built list our own targeted list of special interest. It is resulting to an identical list of m/z searched throughout pixels (corresponding to mass spectra) of the whole set of MSI files. HDI raw outputs were exported in text files and import in R software to further data treatments. Data handling with R included raw data cleaning (image cropping, mis-acquired replicate exclusion) and normalization by total ion count (TIC). A smooth function was applied on the MSI files, in order to limit the artifactual signal variations between pixels. Then, artifactual metabolites were removed: if the mean of the tissue region of interest (tissue ROI, based on Kmeans pixels clustering) was less than the mean of the glass ROI for more than the half of the MSI files, then metabolites were excluded from ulterior analysis. All pixel values corresponding to the tissue replicates were then gathered and analyzed by a centered and non-scaled principal component analysis (PCA). PCA values were then clusterized by the Kmeans method, and each pixel was assigned by a color code depending on its cluster. Images of the replicated tissue sections were then reconstructed from this color code assignation. Finally, each mean values for each ion signal were calculated for each ROI and computed in a heatmap.

Flow cytometry analyses of the immune infiltrate

Tumor infiltrating lymphocytes were isolated and characterized by immunofluorescence staining as described.[20] Orthotopic Hep55.1C tumors were harvested at the end of the experiment (refer to Figure S9A) and dissociated into a single-cell suspension using mechanical and enzymatic disruption, following the manufacturer's instructions (Tumor Dissociation Kit, Miltenyi Biotec, Cat# 130-096-730). For flow cytometry analysis, cells were first stained with a viability dye (Live/Dead Fixable Yellow Dye, Invitrogen, Cat# L34967). Fc receptors were blocked using an anti-mouse CD16/CD32 antibody (BD Biosciences, clone 2.4G2) before incubation with fluorophore-conjugated antibodies targeting specific surface markers: CD45-BUV661 (RRID: AB_2870247), CD3-APC (RRID: AB_10597589), CD4-APC-Vio770 (RRID: AB_2751634), CD8a-PE (RRID: AB_394570), ICOS-BV421 (RRID: AB_2738576), GITR-BV786 (RRID: AB_2740641), LAG3-BV605 (RRID: AB_2742805), PD1-BUV395 (RRID: AB_2742320), TIGIT-BV711 (RRID: AB_2742063), TIM3-PerCP-Cy5.5 (RRID: AB_2561400). For intranuclear staining, cells were fixed and permeabilized using the FoxP3/Transcription Factor Staining Buffer (eBioscience, Thermo Fisher, Cat# 00-5523-00), followed by staining with FoxP3-FITC (RRID: AB_465243). Fluorescence data were acquired using a BD LSRFortessa X20 cytometer with BD FACS Diva software. Compensation, scaling, gating and data analysis were performed using the Omiq.ai (https://www.omiq.ai/) online tool. Gating strategies were determined based on the marker expression profiles of the clusters of interest (refer to Figure S9C). Statistical comparisons were conducted using one-way ANOVA with Sidak's correction for multiple comparisons. Data was exported to R for subsequent analysis (version 4.3.3, https://www.R-project.org/). Samples with low counts in at least one of the cell populations (<5 for tumor samples) were excluded, and counts relative to the number of $CD45^+$ cells as well as geometric means of fluorescence intensity of activation/inhibition markers were Z-score-transformed and plotted as heatmap with the ComplexHeatmap package (version 2.16.0). Statistical comparisons were performed for each variable by ANOVA with two factors (anti-ACBP treatment and anti-PD1 treatment) with the stats package (version 4.3.3) and post-hoc pairwise comparisons with the emmeans package (version 1.10.4).

Liver histology and Immunohistochemistry

Liver tissues in cassettes were fixed with 4% paraformaldehyde for 24h, and transferred into 70% ethanol. After dehydration steps, cassettes were embedded in paraffin. The liver sections were stained with hematoxylin and eosin (H&E) and Sirius red for histology assessment. Slides were scanned and imaged using a slide scanner (Nanozoomer 2.0-HT, Hamamatsu, Japan). NAFLD Activity Score (NAS) was examined by an expert pathologist, who was blinded to the treatments received, according to the NASH-CRN scoring system.[50] Fibrosis stage was evaluated by Sirius red staining and quantified as % Sirius red+ area by QuPath software. The proliferation score was evaluated by Ki67 and PCNA IHC staining of the liver sections. The image analysis was performed with QuPath software as % positive area.

Immunofluorescence

Primary HCC cells ($DBI^{+/+}$ and $DBI^{-/-}$) were seeded at 2000cells/well using four replicates. After 24h, the cells were fixed with 4% PFA/PBS (100 μL/well) for 20 min at room temperature (RT). Fixed plates were washed twice with PBS (200 μL/well), permeabilized and blocked with 0.1% Triton X-100 + 5% BSA +10% FBS for 60 min (100 μL/well) at RT. Primary antibodies were diluted to the appropriate concentration in 1% BSA (100 μL/well), and incubated overnight at 4°C. After two steps of PBS washing (200 μL/well), secondary antibodies (1:250) and DAPI (1:5000) in 1% BSA (50 μL/well) were subsequently added to the cells and incubated for 1 h at RT in the dark. Plates were washed twice with PBS followed by adding PBS (100 μL/well). Image acquisition and analysis were performed with the MetaXpress Image software (Molecular Devices, Sunnyvale, CA, US).

Enzyme-linked immunosorbent assay

For enzyme-linked immunosorbent assays (ELISA), whole blood was collected from mice. Plasma was isolated by centrifugation at 5000 × rpm, 4°C for 10 min. ACBP levels in plasma were assayed by ELISA. High binding ELISA plates were coated with 100 μL anti-ACBP antibodies (human: MBS768488, mouse: ab231910) overnight at 4°C. Plates were washed twice with 100 μL washing buffer (PBS+0.05% Tween 20) and blocked with 200 μL blocking buffer (PBS+1%BSA+0.05% Tween 20) for 2h at RT. After washing, 100 μL standards or diluted plasma samples (human:1/50, mouse:1/20) were added to the plates and incubated for 2h at RT. Plates were washed 3 times with washing buffer, followed by incubation with 1μg/μl anti-ACBP detection antibody (100 μL/well, human: LS-C299614, mouse: MBS2005521) for 1h at RT. After 3 times washing buffer, 100 μL of diluted Avidin-HRP (human:1/5000, mouse:1/1000) was added to each well and incubated at RT for 30 min. The plates were washed 3 times and 100 μL substrates were added, followed by 30-min incubation in dark. After which 50μL stop solution was added and the absorbance at 450 nm was read by a microplate reader.

Quantitative real-time PCR

Liver tissues (about 25mg) were homogenized in QIAzol lysis reagent. Total RNA was extracted and purified with miRNeasy Mini Kit (Cat. 217004, QIAGEN, Germany) according to the manufacturer's instructions. Total RNA (1 000ng) was reversed transcribed into cDNA. Quantitative real-time PCR (qRT-PCR) detection was carried out using PowerUp SYBR Green Master Mix (Cat. A25776, Applied Biosystems, Thermo Fisher Scientific, USA). The primers used in the study were listed in the KEY RESOURCES TABLE.

Immunoblotting

Liver tissues were homogenized in protein lysis buffer (20 mM Tris buffer pH 7.4 + 150 mM NaCl+1% Triton X-100 + 10 mM EDTA +Complete protease inhibitor cocktail) by a Precellys 24 Homogenizer (Bertin Technologies, Montigny-le-Bretonneux, France). Protein extracts were centrifuged at 12000g (4°C) for 15 min to collect supernatants. Protein concentration was determined by BCA protein assay kit (Thermo Fisher Scientific) and samples were then boiled in Laemmli buffer for 10 min at 100°C. Total protein (25 μg) of each sample was loaded onto the 4–12% NuPAGE Bis-Tris gel (Thermo Fisher Scientific) and transferred to PDVF membranes (BioRad). To simultaneously detect different antigens within the same experiment, the membranes were sliced horizontally in several parts based on the molecular weight of the protein of interest. Membranes were blocked with 5% (w/v) BSA in TBST (TBS with 0.1% Tween 20) for 1h at RT and incubated with the appropriate antibodies (KEY RESOURCES TABLE) in 5% (w/v) BSA in TBST overnight at 4°C. Detection was performed with incubation of appropriate horseradish peroxidase (HRP)-conjugated secondary antibodies for 1h at RT. Blots were visualized by ECL detection reagent (GE Healthcare). Densitometric analysis were performed using ImageJ software (NIH). GAPDH serves as a loading control. Densitometric ratios of target protein/GAPDH were normalized to control groups, respectively.

QUANTIFICATION AND STATISTICAL ANALYSIS

Unless indicated otherwise, data are presented as means ± SEM. Normality was assessed by D'Agostino-Pearson, Shapiro-Wilk and Kolmogorov-Smirnov normality tests when use GraphPad Prism 8. Normally distributed data were analyzed by T test, one-way ANOVA or two-way ANOVA. Non-normally distributed data were analyzed using the Welch's t test (two groups), Mann-Whitney U test (two groups) or Kruskal-Wallis test (multiple groups) followed by Dunn's post-test. Body weight curves were analyzed using two-way ANOVA performed through the Tum-Growth online tool (https://kroemerlab.shinyapps.io/TumGrowth/). The log-rank test was used for Kaplan-Meier (KM) survival analyses. Correlations were assessed using Spearman's rank correlation analysis. Unless indicated otherwise, statistical analyses were performed by GraphPad Prism 8.

Bioinformatics analyses

For *DBI* differential expression of cancer patients, RNA-seq data were downloaded from TCGA database (https://portal.gdc.cancer.gov), extracted in TPM format and presented as log2(value + 1). *DBI* differential expression (presented as log2(value)) in HCC patients were further validated by analyzing data from HCC-related GEO datasets (GSE36376, GSE76427, GSE39791, GSE54236, GSE10143, GSE98617, GSE65372, and GSE112790). For HCC patient plasma analysis, continuous variables are presented as median and interquartile range, and categorical variables as number and percentages. Correlations between continuous variables were assessed using Spearman's rank correlation analysis. Survival curves plotted using Kaplan-Meier (KM) analysis was undertaken using survival [3.3.1] survminer, ggplot2[3.3.6] packages. Liver diseases related GEO datasets (GSE120652, GSE164760, GSE74000, GSE120652, GSE87028, GSE103580, GSE49541, GSE45114, GSE47197, GSE34798, GSE98383, GSE107170, GSE15239, GSE119600, GSE7706, GSE14951, GSE23343, and GSE59045) downloaded from the GEO database through the GEOquery package [2.64.2]. The data were normalized with the NormalizeBetweenArrays function of the Limma package [3.52.2]. Only the probe with the largest signal value was retained when encountering multiple probe values for the same gene. GSEA-based KEGG pathway analysis was performed using WebGestalt 2019 (https://www.webgestalt.org/), an online tool for functional enrichment analysis in various biological contexts. The similarity of the global transcriptome dysregulation of the aforementioned liver disease GEO datasets and three MASH-driven mouse RNA-seq datasets in this study (WD + CCl_4_ACBP/DBI knockout, WD + CCl_4_KLH-ACBP, and HFD+DEN_KLH-ACBP) were analyzed by row and column clustering analysis (Euclidean distance) of the normalized enrichment score (NES). We calculated Spearman's correlations between ACBP/DBI and oncogenes in HCC, using data from 33 HCC GEO datasets (refer to Figure S2 for GEO accession numbers) and the TCGA-LIHC dataset. The data were normalized and processed as described above. Statistical analyses and data visualization were performed with R software (4.2.1) and R packages including ggplot2 [3.3.6], stats [4.2.1], car [3.1–0], and ComplexHeatmap [2.13.1].

Cell Reports Medicine, Volume *6*

Supplemental information

Neutralization of acyl coenzyme A binding protein for the experimental prevention and treatment of hepatocellular carcinoma

Sijing Li, Omar Motiño, Flavia Lambertucci, Jonathan Pol, Hui Chen, Long Pan, Sylvère Durand, Federica Rossin, Claudia Campani, Lucie Poupel, Christophe Klein, Léa Montégut, María Pérez-Lanzón, Gerasimos Anagnostopoulos, Uxia Nogueira-Recalde, Alexandra Cerone, Fanny Aprahamian, Yanbing Dong, Manuela Lizarralde-Guerrero, Enfu Xue, Peng Liu, Liwei Zhao, Hui Pan, Vincent Carbonnier, Sylvie Lachkar, Ester Gloria Saavedra Díaz, Li Sun, Chantal Desdouets, Sabine Colnot, Oliver Kepp, Isabelle Martins, Laurence Zitvogel, Mauro Piacentini, Jean-Charles Nault, Maria Chiara Maiuri, Jessica Zucman-Rossi, and Guido Kroemer

Supplemental Figures

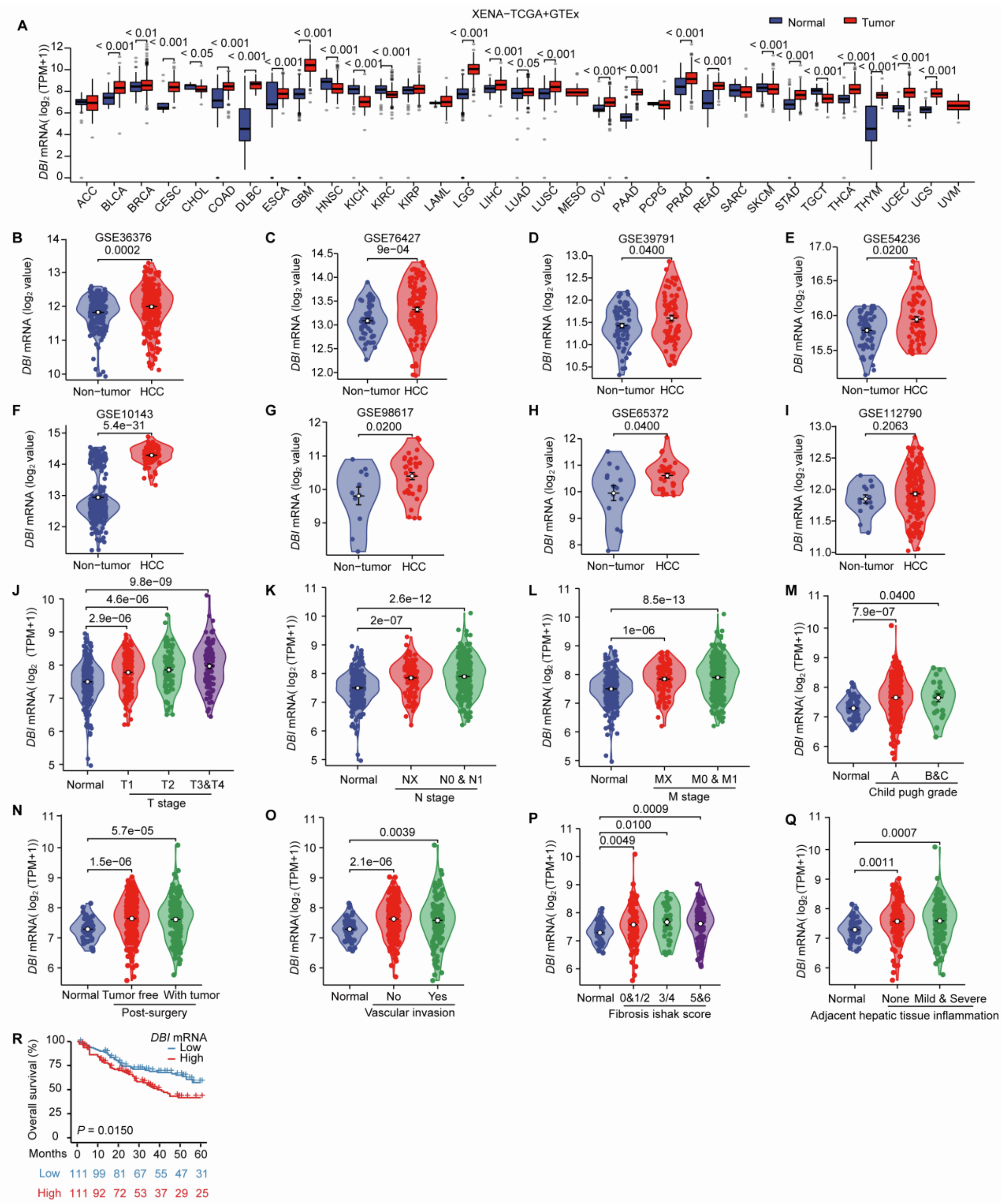

Figure S1 (related to Figure 1). Differential *DBI* mRNA expression in TCGA database. (A) Pan-cancer *DBI* mRNA expression profiles in GEO and TCGA databanks (n = 3-1113/group). (B-I) *DBI* mRNA is significantly upregulated in HCC tissues versus non-tumor tissues in various GEO datasets, including GSE36376 (n = 193-240/group), GSE76427 (n = 52-115/group), GSE39791 (n = 70/group), GSE54236 (n = 64/group), GSE10143 (n = 81-306/group), GSE98617 (n = 11-35/group), GSE65372 (n = 14-33/group), and GSE112790 (n = 15-176/group). (J-M) Changes of *DBI* mRNA in TCGA-LIHC database based on TNM stages and Child-Pugh grade (n = 22-276/group). (N-Q) Differential *DBI* mRNA expression in TCGA-LIHC database with regard to clinical and tumor

characteristics including tumor status, vascular invasion, fibrosis and inflammation (n = 30-212/group). *P* values were calculated by Mann-Whitney U test (A, B, E, F), Welch's t test (C, D, H, I), T test (G), Kruskal-Wallis test with Dunn's post-hoc test (J, K, L, P), and Welch's one-way ANOVA with Games-Howell test (M, N, O, Q). (R) Overall survival of HCC patients based on *DBI* mRNA expression in tumors (n = 111/group). The *P* value was calculated by log-rank test.

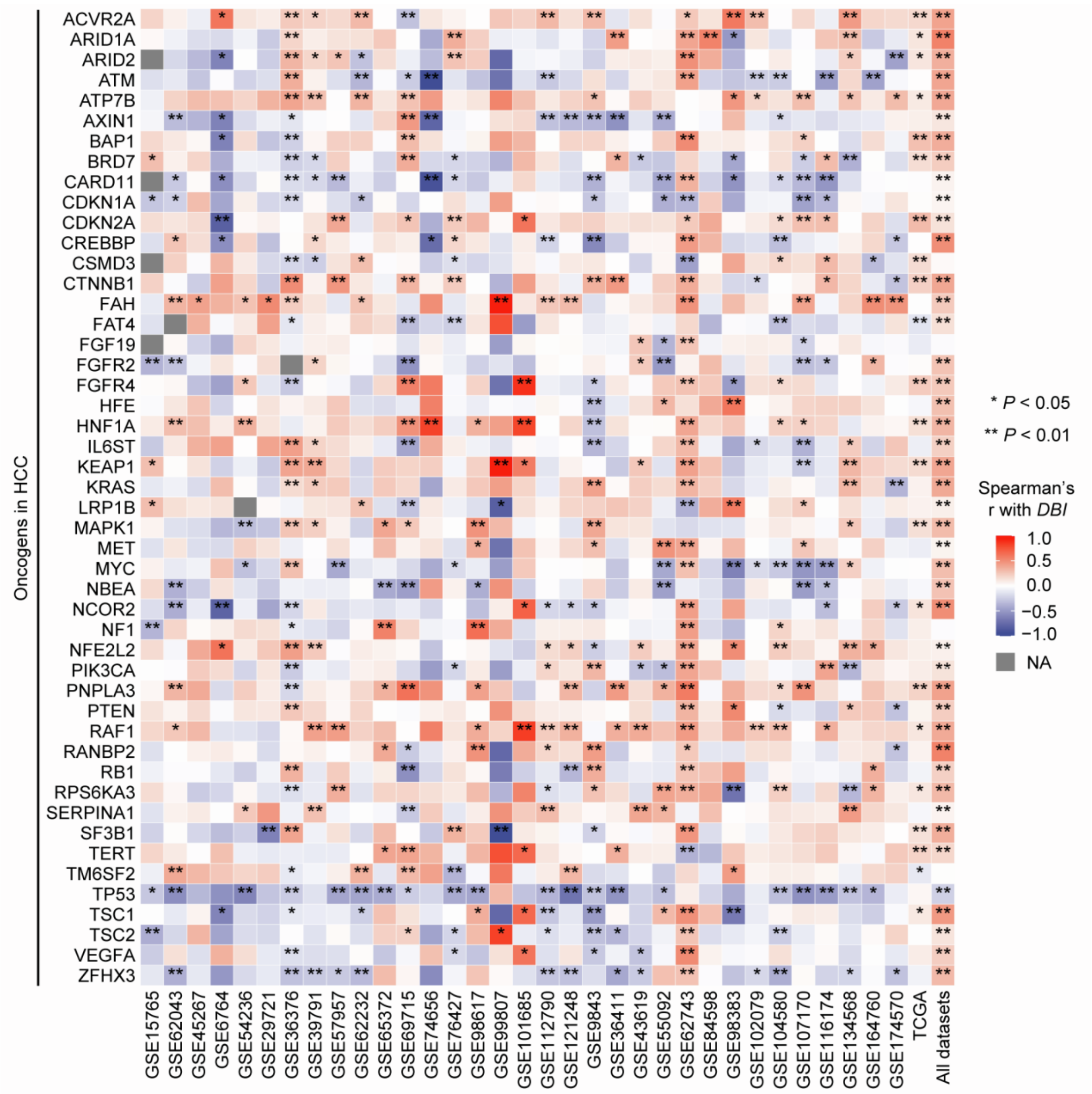

Figure S2 (related to Figure 1). Correlations of ACBP/DBI mRNA levels with mRNAs encoded by oncogenes relevant to human hepatocellular carcinoma. Spearman's coefficient (r) and *P* values were presented in the heatmap for the indicated GSE datasets. NA: not available. **P*<0.05, ***P*<0.01.

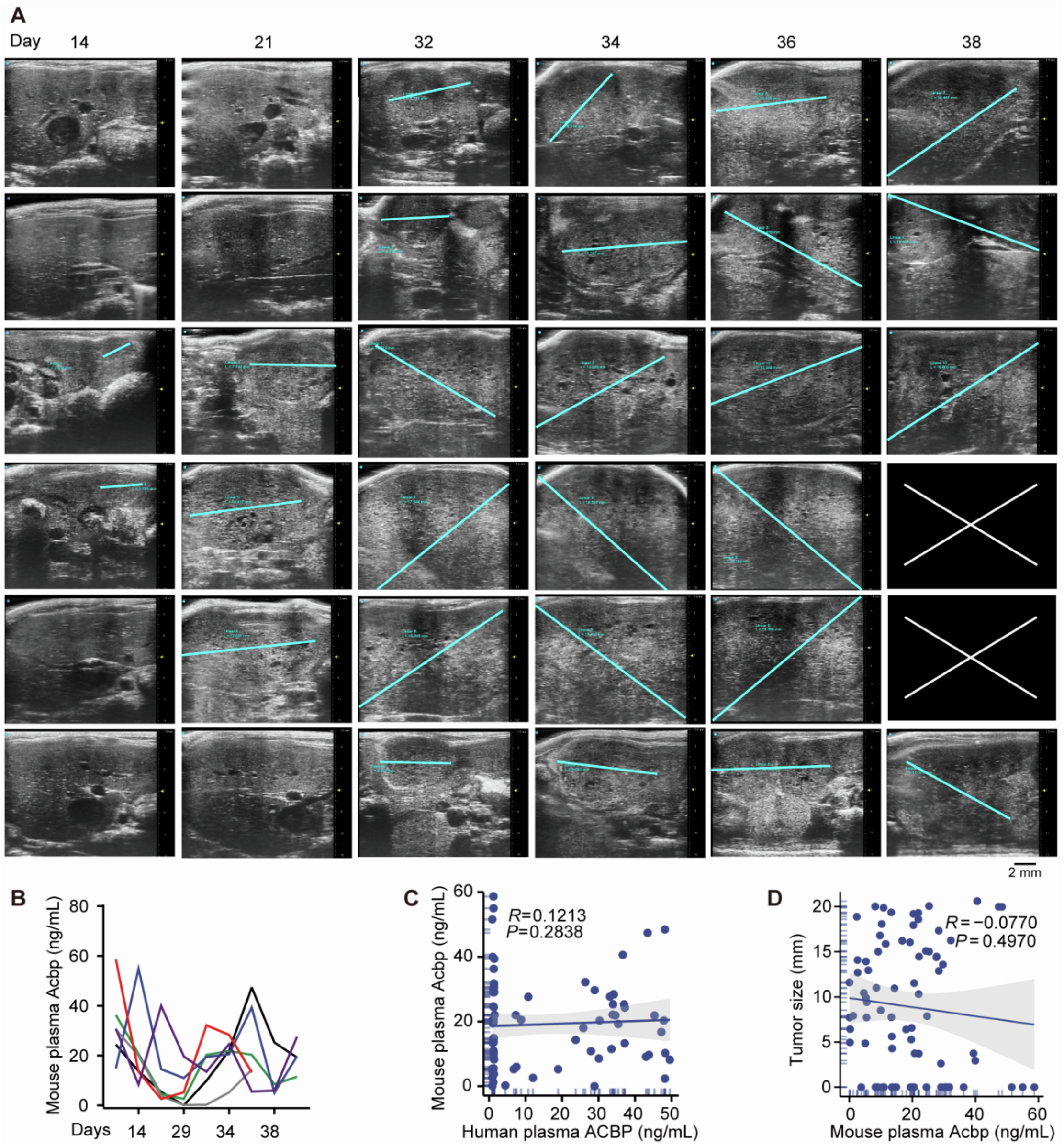

Figure S3 (related to Figure 1). Effect of orthotopic HUH-7 cells implanted in immunodeficient mice on ACBP/DBI levels. (A) Representative images of orthotopic HUH-7 tumors monitored by ultrasonography in nude mice (n = 6). The blue line indicates maximal diameter of a tumor. Scale bar represents 2 mm. Crosses indicate that mice had reached the humane endpoint. (B-D) Effects of HUH-7 cancers inoculated into the liver of immunodeficient *nu/nu* mice (referred to Figure 1M-O) on mouse ACBP/DBI plasma levels over time for individual mice (B) (n = 6), correlation with human ACBP plasma levels (D) (n = 18) and correlation with tumor size (E) (n = 18).

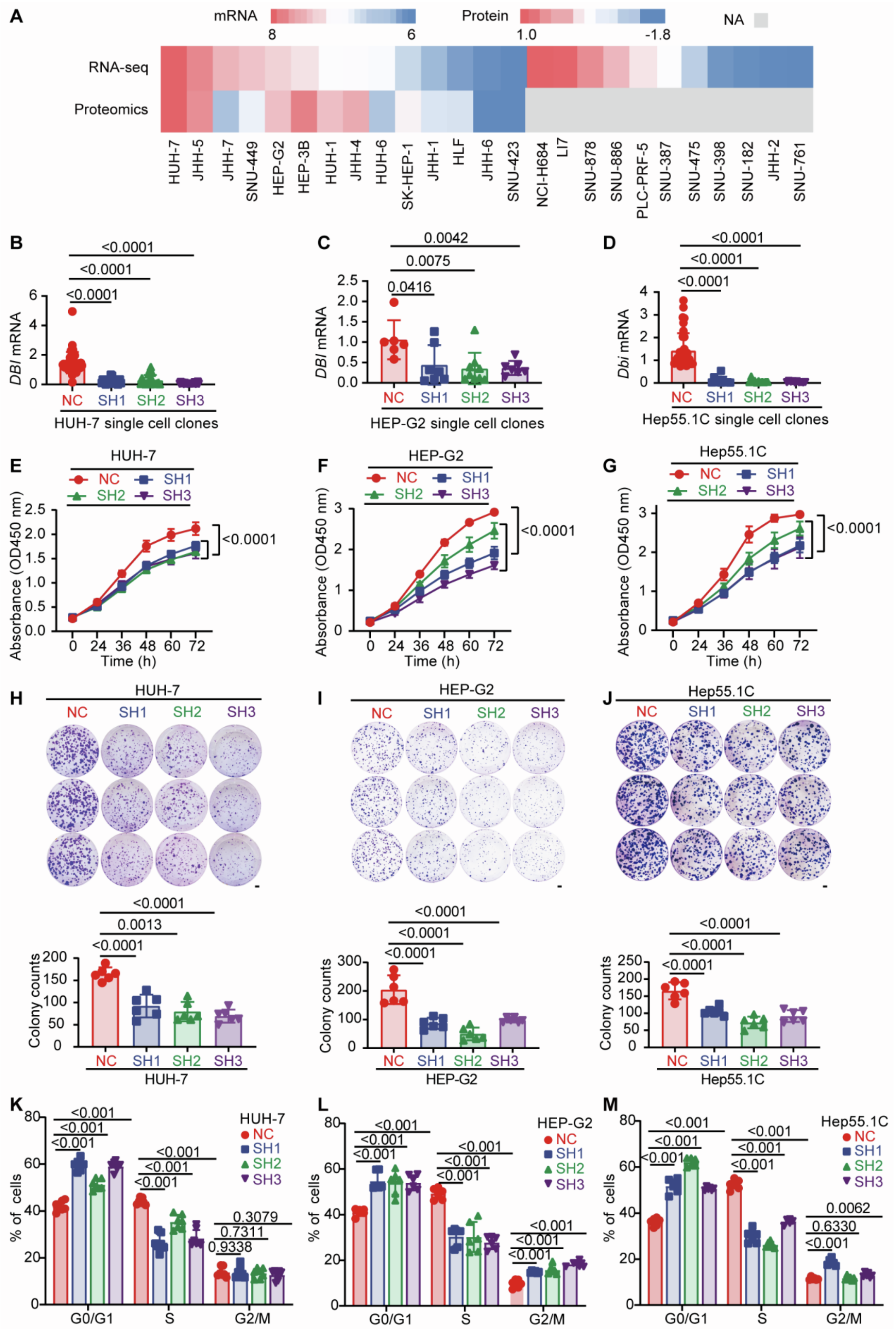

Figure S4 (related to Figure 2). Knockdown of *DBI/Dbi* attenuates proliferation, reduces clonogenic potential and arrests the cell cycle of liver cancer cells. (A) ACBP/DBI mRNA and protein expression profiles in different liver cancer cell lines. Data were extracted from the CCLE database (https://sites.broadinstitute.org/ccle/). (B-D) *DBI/Dbi* depletion efficiency verified by

qRT-PCR in single cell clones, derived from distinct parental cell lines including HUH-7, HEP-G2 and Hep55.1C (n = 6-43/group). (E-G) CCK-8 proliferation assay of HUH-7, HEP-G2 and Hep55.1C-derived *DBI/Dbi* knockdown and control cell lines (n = 6/group). (H-J) Representative images and quantifications of colony formation assays (n = 6/group) and (K-M) cytofluorometric analysis of cell cycle distributions of the aforementioned liver cancer cell lines. Experiments were performed at least three times (n = 6/group). Scale bar represents 3.5 mm. Error bars represent means ± SEM. *P* values were calculated by Kruskal-Wallis test with Dunn's post-hoc test (B, C, D), two-way ANOVA (E, F, G), one-way ANOVA post hoc Tukey HSD test (H, J), Welch's one-way ANOVA with Games-Howell test (I), and Kruskal-Wallis test with Dunn's post-hoc test (K, L, M), and Mann-Whitney U (P).

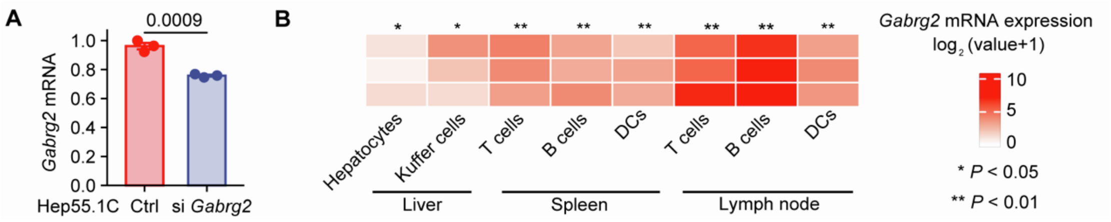

Figure S5 (related to Figure 2). *Gabrg2* mRNA expression in various cell types. (A) Specificity of the *Gabrg2* primer, as assessed by qRT-PCR in negative control (Ctrl) and *Gabrg2*-deficient (si *Gabrg2*) Hep55.1C cells (n = 3/group). The gene 36b4 was utilized as a housekeeping gene. Statistical significance (Ctrl vs. Si *Gabrg2*) was determined using Student's t-test. (B) *Gabrg2* mRNA expression profile in isolated primary hepatocytes, Kupffer cell from liver and immune cells from the spleen and lymph nodes, including T cells, B cells, and dendritic cells (DCs) (n = 3/group). P-values were calculated (relative to an internal control) by means of Student's t-test.

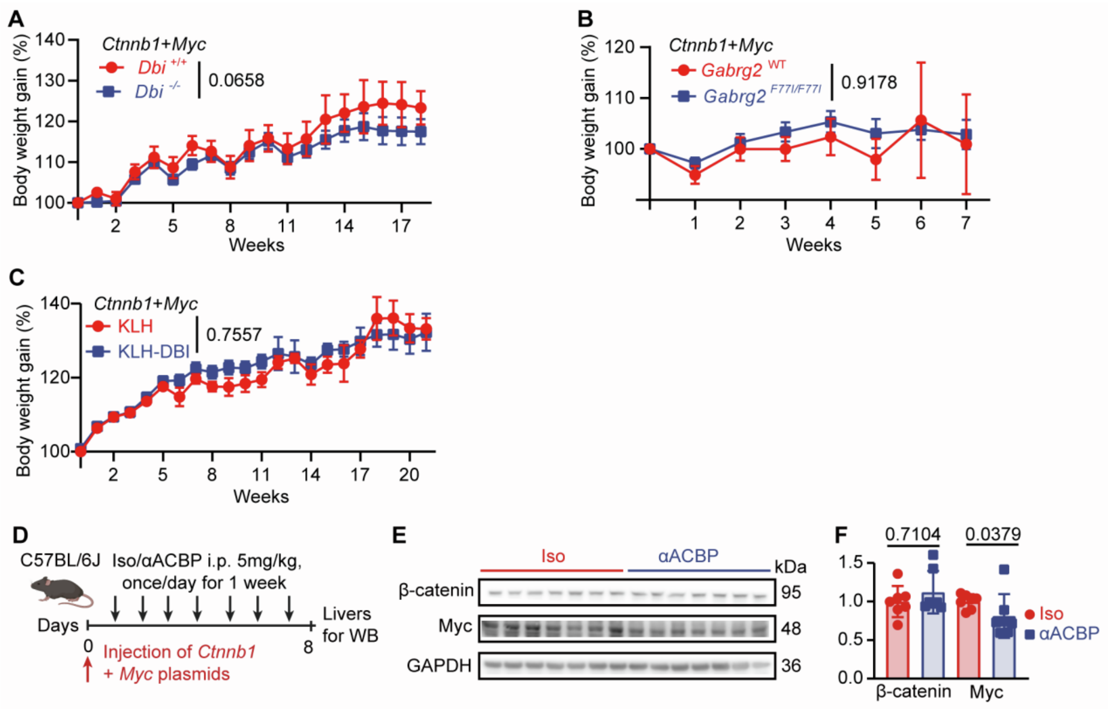

Figure S6 (related to Figure 3). Effects of ACBP/DBI inhibition on body weight changes in *Myc/Ctnnb1*-induced hepatic carcinogenesis mouse models. (A-C) Body weight gain (%) from initial weight throughout the different *in vivo* models shown in Figure 3A, 3E, and 3I (n = 8-12/group). (D) Schematic diagram of ACBP/DBI blockade in *Myc*/*Ctnnb1*-induced hepatic carcinogenesis model. (E-F) Western blots (E) and quantifications (F) of Myc and β-catenin in the whole liver tissues from the mouse model shown in (D) (n = 7/group). *P* values were calculated by two-way ANOVA (A-C) and Mann-Whitney U (F).

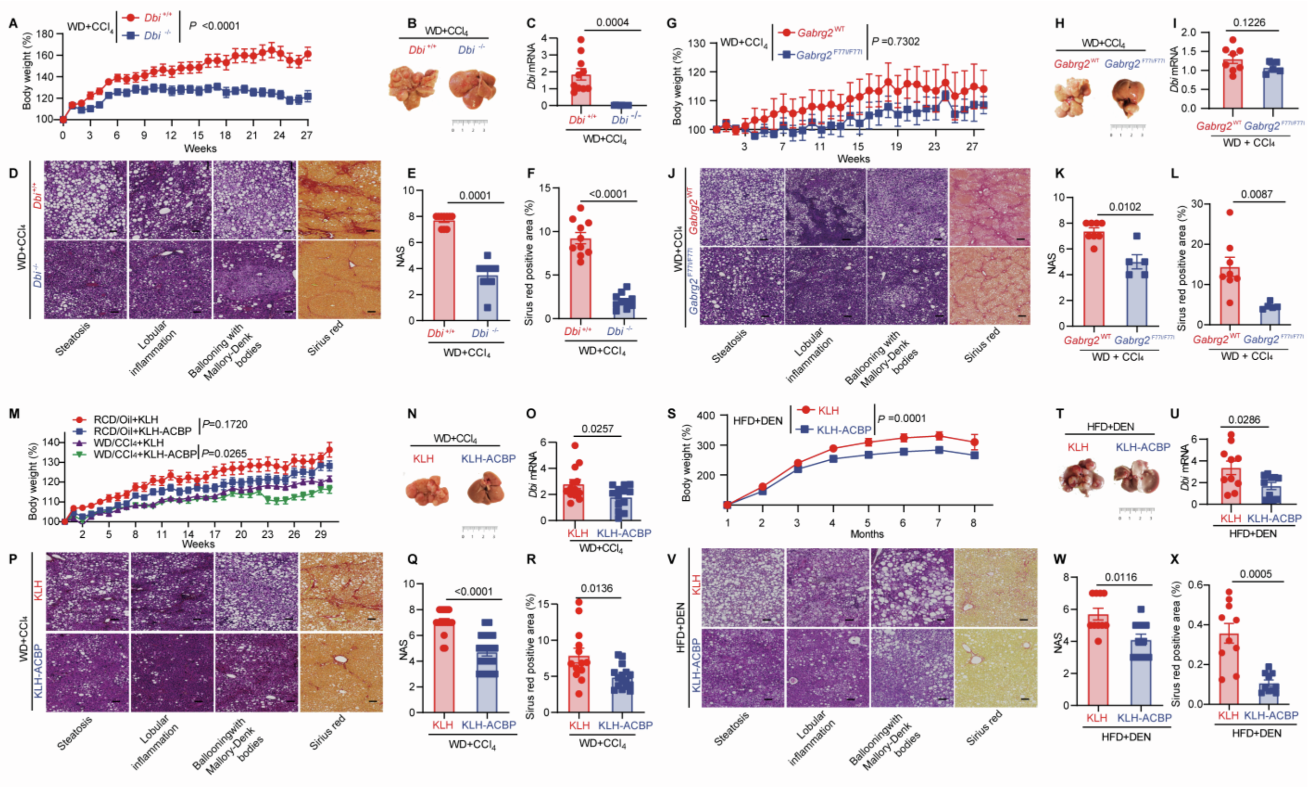

Figure S7 (related to Figure 4). Intra- and extracellular ACBP/DBI inhibition slows MASH/MASH-induced fibrosis. (A, G, M, S) Body weight gain (%) from initial weight throughout the different *in vivo* models (n = 5-27/group). (B, H, N, T) Representative photos of representative livers from each treatment group. (C, I, O, U) *Dbi* mRNA levels in each treatment group (n = 5-13/group). (D, J, P, V) Representative pictures of H&E and Sirius Red staining in each study. Size bar: 200 µm. (E, K, Q, W) NAFLD activity score (NAS) was assessed as the sum of steatosis, lobular inflammation, and ballooning scores for each mouse (n = 5-13/group). (F, L, R, X) Quantification of Sirius Red-positive areas (%) of liver sections in randomly selected liver samples for each treatment group (n = 5-13/group). Error bars represent means ± SEM. *P* values were calculated by two-way ANOVA (A, G, M, S), Welch's t test (C, F, I, R, U, X), T test (L, O), and Mann-Whitney U test (E, K, Q, W).

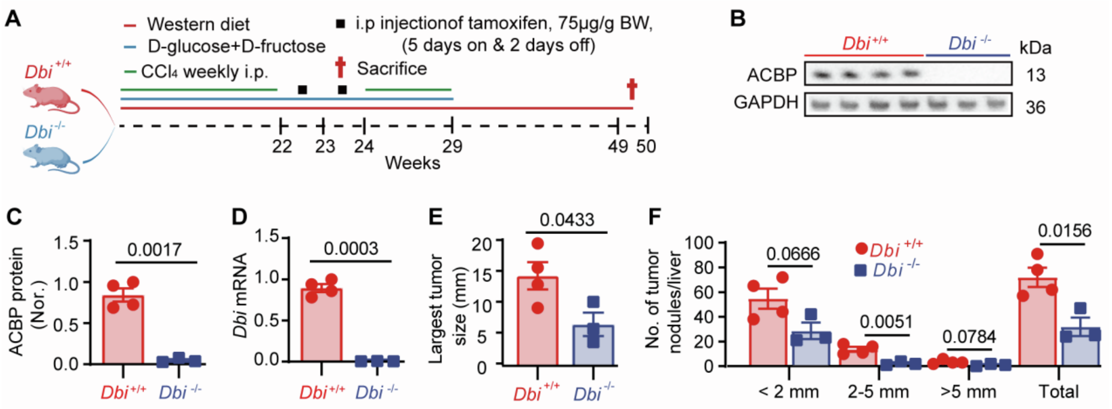

Figure S8 (related to Figure 4). Liver-specific conditional ACBP/DBI knockout suppressed MASH-driven hepatocarcinogenesis. (A) Schematic diagram of hepatic carcinogenesis induced by Western-style diet (WD) plus CCl_4 in tamoxifen-induced liver-specific conditional *Dbi* knockout mice (*Dbi*$^{-/-}$) and control mice (*Dbi*$^{+/+}$). (B) Representative immunoblots detecting ACBP protein in the liver. GAPDH was used as a loading control (n = 3-4/group). (C) Quantification of Western blots (n = 3-4/group). Densitometric ratios of ACBP/GAPDH were normalized to control groups, shown as arbitrary units (Nor.). (D) *Dbi* mRNA levels in each treatment group (n = 3-4/group). (E) Quantification of the largest tumor size of each treatment group (n = 3-4/group). (F) Quantification of the number of tumors with different size (n = 3-4/group). Error bars represent mean ± SEM. *P* values were calculated by Welch's t test (C-E) and multiple t test with Holm-Sidak multiple test correction (F).

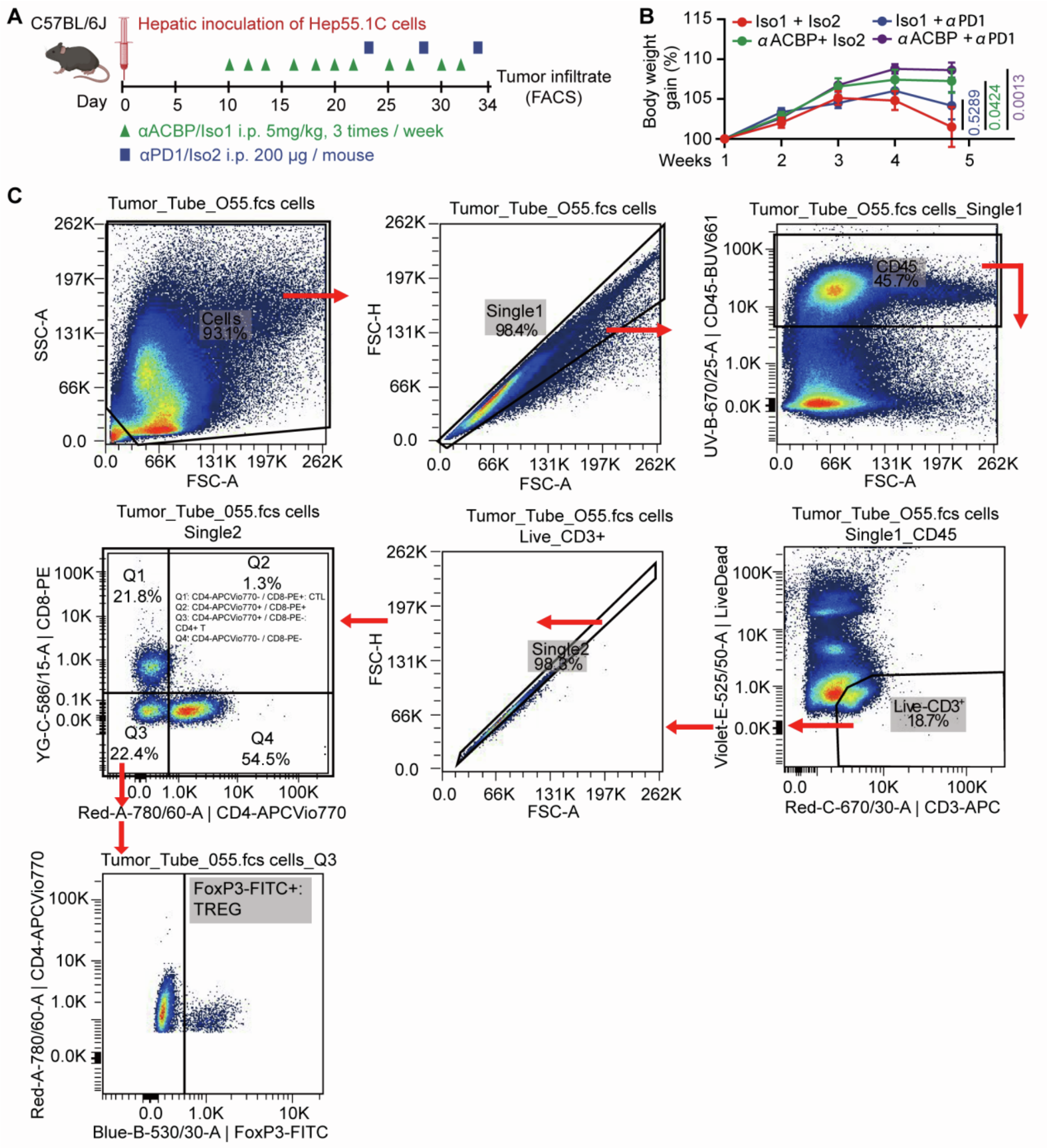

Figure S9 (related to Figure 5). Analysis of the T lymphocyte infiltration in the orthotopic Hep55.1C HCC model treated with anti-ACBP plus anti-PD1. (A) Schematic diagram of experimental flow. (B) Body weight gain (%) from initial weight of the treated mice (n = 9-13/group). *P* values were calculated by two-way ANOVA. (C) Representative immunofluorescence cytometry pictures and gating strategies of T cell infiltrating analyses.

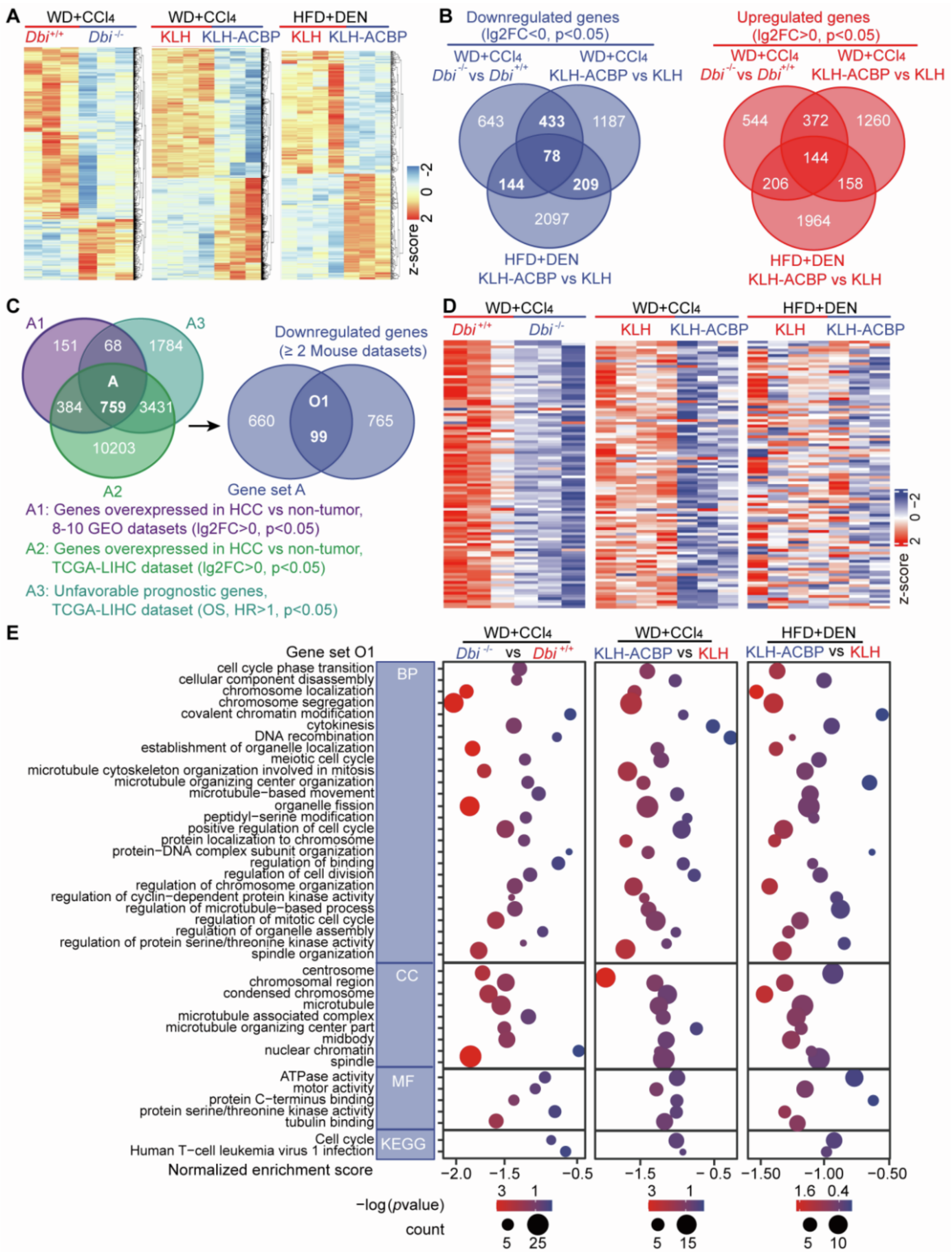

Figure S10 (related to Figure 5). Transcriptomic signatures of ACBP/DBI inhibition in MASH-driven HCC models. (A) Heatmap of the gene expression profiles in three NASH-driven HCC mouse models (n = 3-4/group). (B) Commonly up- or downregulated genes in three mouse models. (C) The flow diagram summarizes the strategy to identify gene set O1. Gene set O1 was defined as genes downregulated by ACBP/DBI inhibition in ≥2 mouse NASH models that are also overexpressed in human HCC, as well as associated with poor prognosis in TCGA-LIHC. (D) Heatmap of gene set O1 in (C) (n = 3-4/group). (E) Gene ontology (GO) and KEGG pathway analysis of gene set O1. Enriched GO-terms for biological process (BP), molecular function (MF), and cellular component (CC) were shown. The pathways were sorted alphabetically in each class. *P* values were calculated using T test (E).

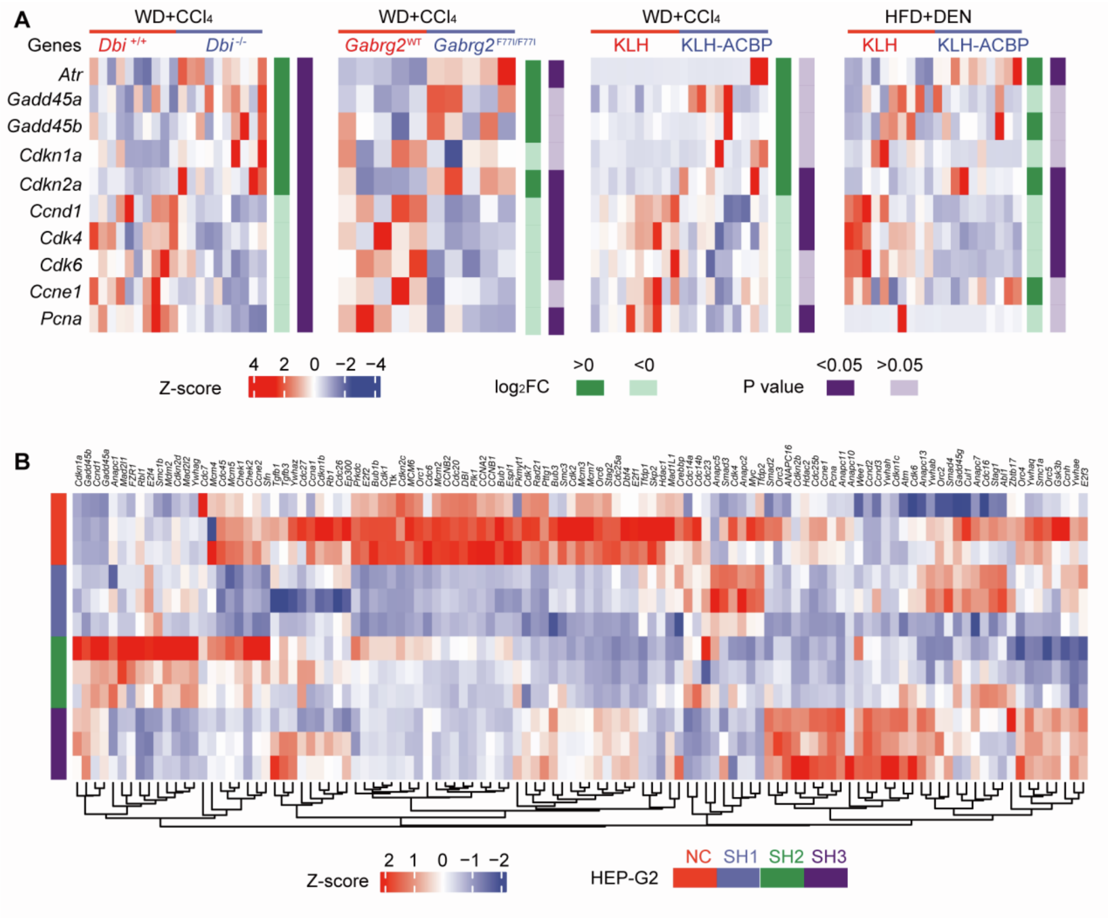

Figure S11 (related to Figure 5). ACBP/DBI inhibition regulates cell cycle-relevant genes. (A) ACBP/DBI inhibition downregulated genes that were positive regulators of cell cycle (*Ccnd1*, *Cdk4*, *Cdk6*, *Ccne1* or *Pcna*) and upregulated genes that inhibit cell cycle progression (*Atr*, *Gadd45a*, *Gadd45b*, *Cdkn1a* or *Cdkn2a*) in four NASH-driven HCC models (n = 5-10/group). *P* values were calculated by T test, Welch's t test, or Mann-Whitney U test, as applicable. (B) Heatmap showing the expression profile of cell cycle-related genes in HEP-G2-derived *DBI* knockdown cell lines (n = 3/group).

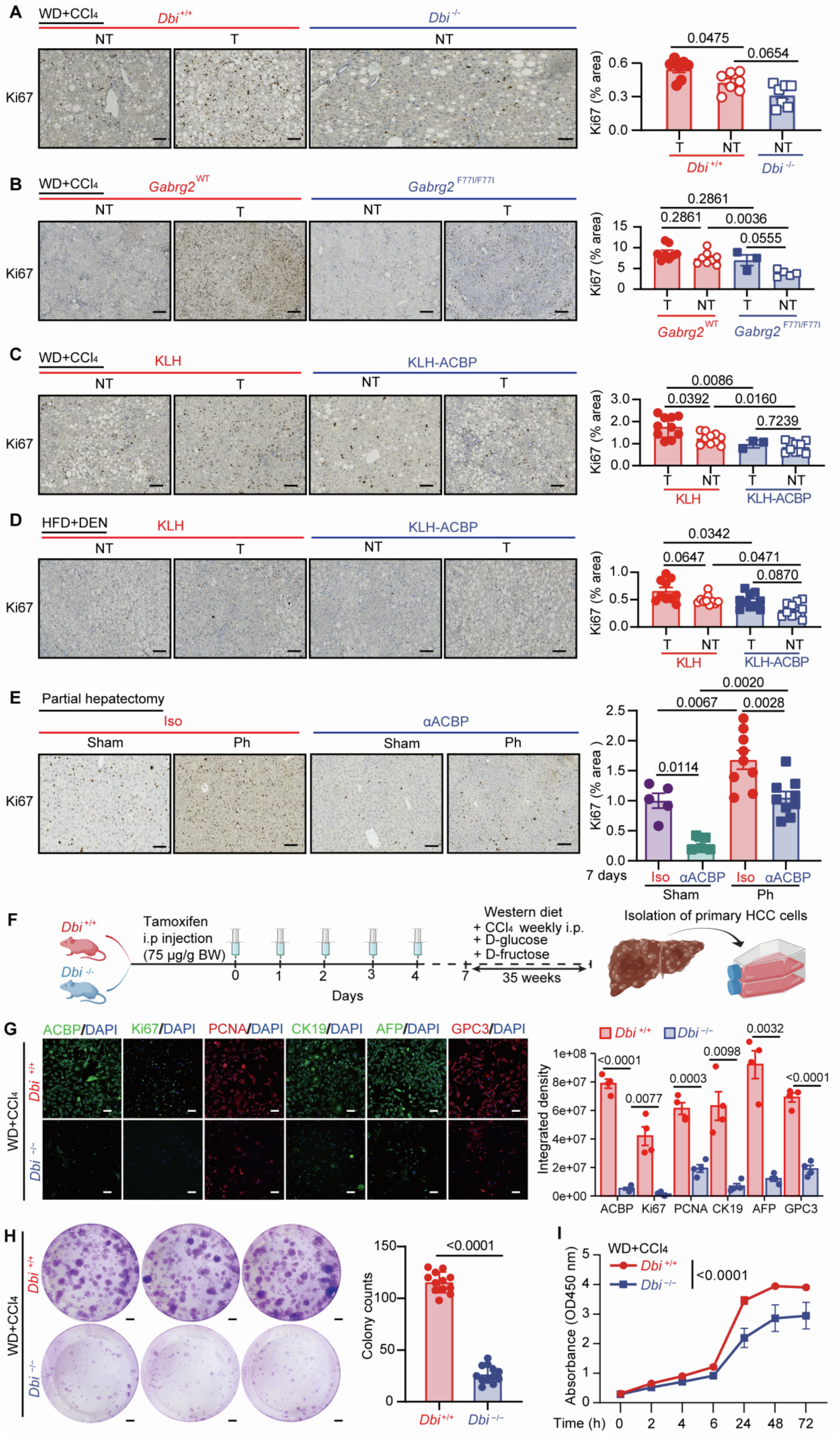
A
WD+CCl4
Dbi +/+
Dbi -/-
NT
T
NT
Ki67
0.0475
0.0654
Ki67 (% area)
0.6
0.3
0.0
T
NT
NT
B
Gabrg2 WT
Gabrg2 F77I/F77I
0.2861
0.2861
0.0036
0.0555
15
10
5
0
C
KLH
KLH-ACBP
0.0086
0.0392
0.0160
0.7239
3.0
2.0
1.0
0.0
D
HFD+DEN
0.0342
0.0647
0.0471
0.0870
1.0
0.5
0.0
E
Partial hepatectomy
Iso
αACBP
Sham
Ph
0.0020
0.0067
0.0028
0.0114
2.5
2.0
1.5
1.0
0.5
0.0
7 days
F
Tamoxifen
i.p injection
(75 μg/g BW)
0
1
2
3
4
7
Days
Western diet
+ CCl4 weekly i.p.
+ D-glucose
+ D-fructose
35 weeks
Isolation of primary HCC cells
G
ACBP/DAPI
Ki67/DAPI
PCNA/DAPI
CK19/DAPI
AFP/DAPI
GPC3/DAPI
Integrated density
1e+08
8e+07
6e+07
4e+07
2e+07
0e+00
<0.0001
0.0077
0.0003
0.0098
0.0032
<0.0001
ACBP
Ki67
PCNA
CK19
AFP
GPC3
H
Colony counts
150
100
50
0
<0.0001
I
Absorbance (OD450 nm)
5
4
3
2
1
0
Time (h)
0
2
4
6
24
48
72

Figure S12 (related to Figure 5). ACBP/DBI inhibition decreased cell proliferation. (A-E) Representative images and quantification of Ki67 IHC staining in liver sections from distinct mouse models (n = 5-10/group). T/NT represents Tumor/Non-tumor. Scale bars represent 200 μm. (F) Schematic diagram of the strategy to generate primary *Dbi*$^{+/+}$ and *Dbi*$^{-/-}$ HCC cells in the WD+CCl_4-induced mouse model. (G) Representative images of ACBP, Ki67, PCNA, CK19, AFP and GPC3 immunofluorescence staining in primary *Dbi*$^{+/+}$ and *Dbi*$^{-/-}$ HCC cells and quantification (n = 4/group). Scale bars represent 100 μm. (H) Representative images and quantification of colony formation of primary *Dbi*$^{+/+}$ and *Dbi*$^{-/-}$ HCC cells (n = 12/group). Scale bar represents 3.5 mm. (I) CCK-8 proliferation assay in HCC primary cells. *In vitro* experiments were repeated at least three times (n = 6/group). Error bars represent means ± SEM. *P* values were calculated by one-way ANOVA (A, B, D, E), Welch's one-way ANOVA with Games-Howell test (C), Welch's t test (G), T test (H), and two-way ANOVA (I).

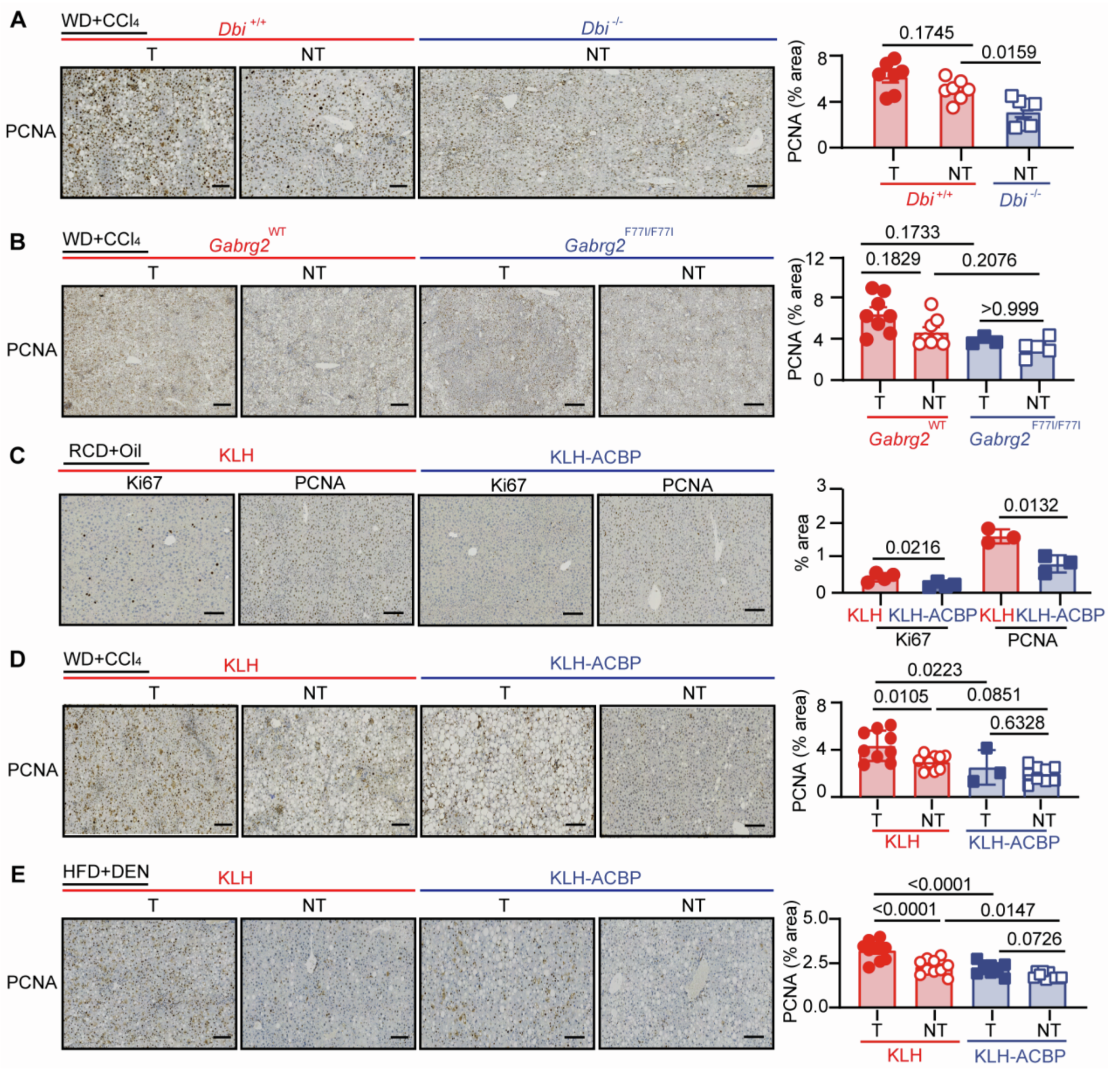

Figure S13 (related to Figures 4 & 5). ACBP/DBI inhibition attenuated cell proliferation. (A-E) Representative images and quantification of PCNA/Ki67 immuno-staining in liver sections from distinct mouse models (n = 3-10/group). Scale bars represent 200 µm. Error bars represent means ± SEM. *P* values were calculated by one-way ANOVA post hoc Tukey HSD test (A), Kruskal-Wallis test with Dunn's multiple comparison (B), T test (C), and One-way ANOVA with Dunnett's multiple comparison test (D, E).

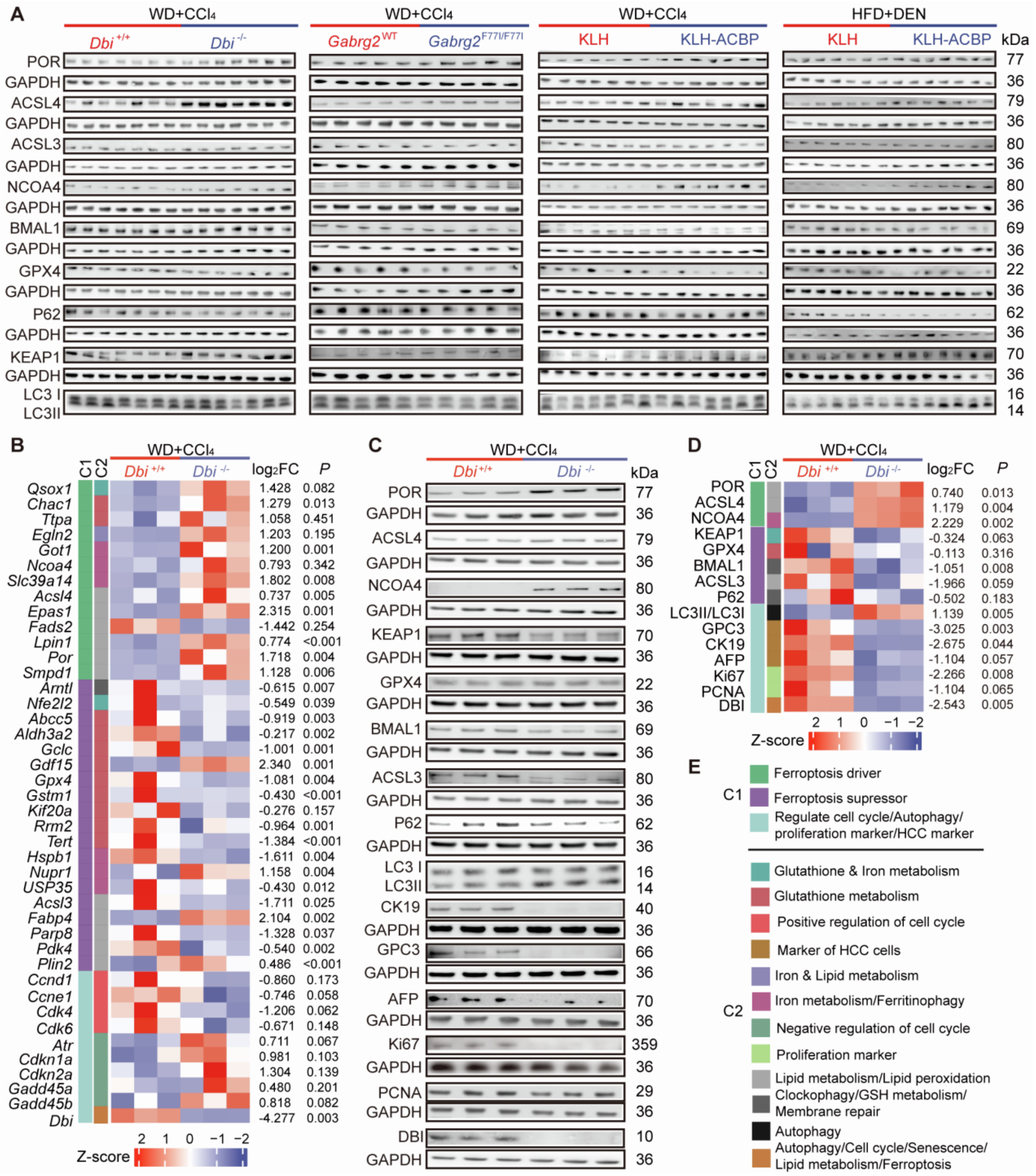

Figure S14 (related to Figures 4, 5 & 6). ACBP/DBI knockout regulate ferroptosis gene/protein signature in primary HCC cells. (A) Western blots of ferroptosis protein signature in liver tissues from different NASH-HCC mouse models (n = 5-7/group). (B) qRT-PCR analysis (n = 3/group). Ferroptosis and cell cycle-related gene expression profiles in primary *Dbi*$^{+/+}$ and *Dbi*$^{-/-}$ HCC cells were shown in a heatmap. (C-D) Immunoblotting and densitometric analysis of the blots (n = 3/group). Expression of ferroptosis-related proteins, malignancy-linked markers and proliferation markers was determined by immunoblotting in primary *Dbi*$^{+/+}$ and *Dbi*$^{-/-}$ HCC cells. The normalized values were shown in a heatmap. (E) Functional annotations of genes/proteins in (B-D). *P* values were calculated by T test, Welch's t test, or Mann-Whitney U test (B, D).

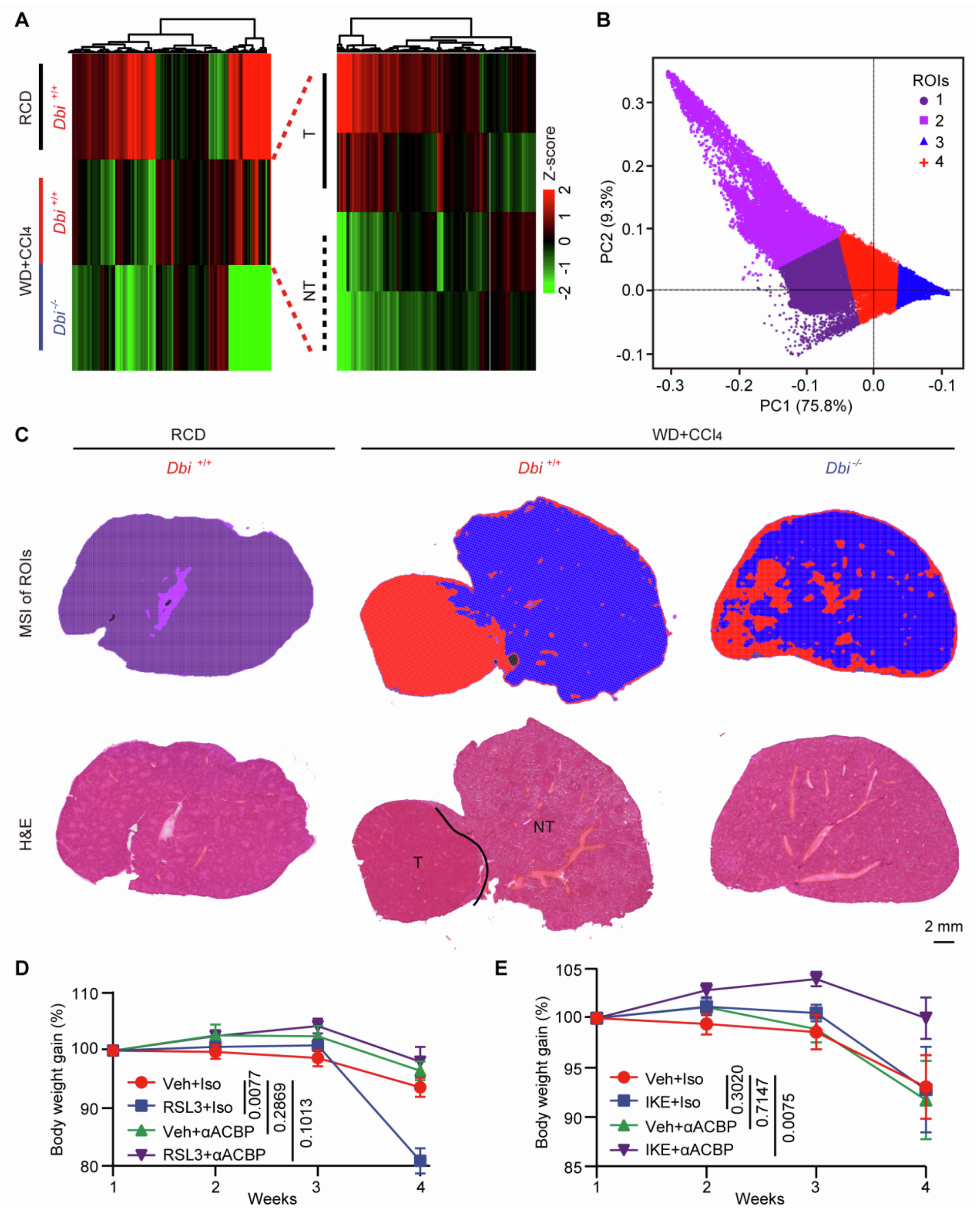

Figure S15 (related to Figures 6 & 7). Spatial metabolomic landscape of liver sections from WD/CCl4-induced HCC models. (A) Heatmap depicting the average metabolite intensities across three representative liver lobes. The left part of the heatmap displays metabolite signatures in liver sections from *Dbi*$^{+/+}$-RCD, *Dbi*$^{+/+}$-WD/CCl4, and *Dbi*$^{-/-}$-WD/CCl4 mice (n = 1/group). Rows are mean of the replicates per condition. The right part of the heatmap represents the metabolite signatures of tumor (T) and non-tumor (NT) liver sections of *Dbi*$^{+/+}$-WD/CCl4 mice. Rows indicate means of the replicates per condition (left) after integration of all data points and columns are putative metabolites. Hierarchal clustering of the metabolite intensities was performed based on Euclidean distance. Color scale (red/green) represents normalized metabolite intensities (log2), and the missing values are shown in grey. (B) Principal component analysis (PCA) of the mass spectrometry imaging

(MSI) data from regions of interest (ROI) of the aforementioned three liver lobes (as defined in C). All replicates from the representative liver lobes were used. Colors represent Kmeans clustering of pixels (metabolites intensities) in liver section. (C) Representative pictures of negative polarity MSI and H&E staining of livers. The color code corresponds to that in (B). (D-E) Body weight gain (%) from initial weight of the treated mice as seen from Figure 7E and Figure 7K (n = 9/group). *P* values were calculated by two-way ANOVA.

Supplemental Table

Table S1. Genes used for calculating Immunosuppression and Immunostimulation scores (related to Figure 6E).

Immunosuppression score	Immunostimulation score
Ptprc	Cd27
Cd3g	Cd276
CD8a	Cd28
Cd4	Cd40
PDCD1	Cd40lg
Lag3	Cd48
Tigit	Cd70
Tnfrsf18	Cd80
Vsir	Cd86
Havcr2	Cxcl12
Foxp3	Cxcr4
	Entpd1
	Icos
	Icosl
	Il2ra
	Il6
	Il6ra
	Klrc1
	Lta
	Mill2
	Nt5e
	Tmem173
	Tnfrsf13b
	Tnfrsf13c
	Tnfrsf14
	Tnfrsf17
	Tnfrsf18
	Tnfrsf25
	Tnfrsf4
	Tnfrsf8
	Tnfrsf9
	Tnfsf13
	Tnfsf13b
	Tnfsf14
	Tnfsf15
	Tnfsf18
	Tnfsf4
	Ulbp1
